# Genome Sequencing and Analysis of the Peanut B-Genome Progenitor (*Arachis ipaensis*)

**DOI:** 10.3389/fpls.2018.00604

**Published:** 2018-05-03

**Authors:** Qing Lu, Haifen Li, Yanbin Hong, Guoqiang Zhang, Shijie Wen, Xingyu Li, Guiyuan Zhou, Shaoxiong Li, Hao Liu, Haiyan Liu, Zhongjian Liu, Rajeev K. Varshney, Xiaoping Chen, Xuanqiang Liang

**Affiliations:** ^1^South China Peanut Sub-Center of National Center of Oilseed Crops Improvement, Guangdong Provincial Key Laboratory of Crop Genetic Improvement, Crops Research Institute, Guangdong Academy of Agricultural Sciences, Guangzhou, China; ^2^Shenzhen Key Laboratory for Orchid Conservation and Utilization, National Orchid Conservation Center of China and Orchid Conservation and Research Center of Shenzhen, Shenzhen, China; ^3^International Crops Research Institute for the Semi-Arid Tropics, Hyderabad, India; ^4^School of Plant Biology, The Institute of Agriculture, University of Western Australia, University of Western Australia, Crawley, WA, Australia

**Keywords:** *Arachis ipaensis*, genome sequence, genome evolution, polyploidizations, whole genome duplication

## Abstract

Peanut (*Arachis hypogaea* L.), an important leguminous crop, is widely cultivated in tropical and subtropical regions. Peanut is an allotetraploid, having A and B subgenomes that maybe have originated in its diploid progenitors *Arachis duranensis* (A-genome) and *Arachis ipaensis* (B-genome), respectively. We previously sequenced the former and here present the draft genome of the latter, expanding our knowledge of the unique biology of *Arachis*. The assembled genome of *A. ipaensis* is ~1.39 Gb with 39,704 predicted protein-encoding genes. A gene family analysis revealed that the FAR1 family may be involved in regulating peanut special fruit development. Genomic evolutionary analyses estimated that the two progenitors diverged ~3.3 million years ago and suggested that *A. ipaensis* experienced a whole-genome duplication event after the divergence of *Glycine max*. We identified a set of disease resistance-related genes and candidate genes for biological nitrogen fixation. In particular, two and four homologous genes that may be involved in the regulation of nodule development were obtained from *A. ipaensis* and *A. duranensis*, respectively. We outline a comprehensive network involved in drought adaptation. Additionally, we analyzed the metabolic pathways involved in oil biosynthesis and found genes related to fatty acid and triacylglycerol synthesis. Importantly, three new *FAD2* homologous genes were identified from *A. ipaensis* and one was completely homologous at the amino acid level with FAD2 from *A. hypogaea*. The availability of the *A. ipaensis* and *A. duranensis* genomic assemblies will advance our knowledge of the peanut genome.

## Inroduction

Peanut (*Arachis hypogaea* L.) is a grain legume and oilseed crop that is an important source of vegetable oil and protein. It is widely cultivated in tropical and subtropical regions. In Africa and some Asia countries, peanut is more prevalent than any other leguminous crop, including soybean. With an annual production of ~46 million tons and a remarkable 45–56% oil content, it plays a key role in daily human nutrition. Moreover, peanut oil is important to human health owing to its rich nutritional elements, such as oleic acid, linoleic acid, resveratrol, fiber, and vitamins (Parthasarathy et al., 1990).

The *Arachis* genus originated in South America and is composed of about 80 diploid species that have been divided taxonomically into nine sections (Krapovickas and Gregory, 1994). *Arachis* species have an unusual reproductive biology in that all members have a geocarpic reproductive habit, with unique growth characteristics of aerial flowers and subterranean fruit (Smith, 1950), that allows them to adapt to particularly harsh environments (Tan et al., 2010). *A. hypogaea*, cultivated peanut or groundnut, is an allotetraploid (2n = 4x = 40), with an AABB genomic constitution (Temsch and Greilhuber, 2000), which was probably derived from a single recent hybridization of two diploid progenitors (Kochert et al., 1991, 1996; Moretzsohn et al., 2013). Molecular evidence indicates that *Arachis duranensis* and *Arachis ipaensis* are the two most likely progenitors that donated the A and B subgenomes, respectively (Kochert et al., 1996; Ramos et al., 2006; Grabiele et al., 2012; Moretzsohn et al., 2013). The genome sizes of the two species are ~1.25 and ~1.56 Gb, respectively (Samoluk et al., 2015), and their sum is close to the total genome size of *A. hypogaea* (~2.8 Gb) (Temsch and Greilhuber, 2000), indicating that no large changes that affected genome size have taken place since polyploidy. Moreover, researches indicated that the genomes of *A. duranensis* and *A. ipaensis* are similar to cultivated peanut's A and B subgenomes (Kochert et al., 1996; Seijo et al., 2007; Robledo et al., 2009; Robledo and Seijo, 2010; Moretzsohn et al., 2013). The high-DNA identity between the *A. ipaensis* genome and the B subgenome of cultivated peanut, along with biogeographic evidence, indicates that *A. ipaensis* may be the direct descendant of *A. hypogaea* that contributed the B subgenome (Bertioli et al., 2016).

The large genome size of *A. hypogaea* (~2.8 Gb) and highly repetitive content (64%) makes the assembly of the peanut genome sequence very challenging (Dhillon et al., 1980; Temsch and Greilhuber, 2000; Bertioli et al., 2016). Therefore, sequencing and analyzing the genomes of the two diploid ancestors to uncover the genome of cultivated peanut was considered a sensible initial strategy. Our previous sequencing of the peanut A-genome progenitor, *A. duranensis*, provided new insights into *Arachis* biology, evolution and genomic changes (Chen et al., 2016). To gain insights into the genomic evolution, as well as the divergence, of the peanut B subgenome and to provide candidate genes to enable a better understanding of the biology of leguminous species, we sequenced the suspected peanut B-genome progenitor, *A. ipaensis*, and re-sequenced two A-genome and three B-genome genotypes (Chen et al., 2016). The *A. ipaensis* genome sequencing will facilitate future research on the genome assembly of cultivated peanut and, has the potential to accelerate the molecular breeding of peanut varieties.

## Results and discussion

### Genome sequencing, assembly, and annotation

The genome of the peanut B-genome progenitor, *A. ipaensis* (ICG_8206), was sequenced using a shotgun approach on the Illumina HiSeq2500 platform (Supplementary File 1: Figure S1). We generated 250.40 Gb of high-quality reads, representing 149.53 × genome coverage, with fragment lengths ranging from 250 to 20 Kb (Supplementary File 1: Table S1). A total of ~1,391.70 Mb of the *A. ipaensis* genome sequence was assembled using SOAPdenovo2 (Luo et al., 2012) with a contig N50 of 8,067 bp and a scaffold N50 of 170,050 bp (Table 1; Supplementary File 1: Tables S2, S3). An assessment of the draft genome assembly using the core eukaryotic gene mapping approach method (Parra et al., 2007) revealed that >98% of conserved genes were present in the assembly (Supplementary File 1: Table S4). Over 98% of transcript sequences (>500 bp) were mapped to the assembled genome (Supplementary File 1: Table S5). Based on *k*-mer statistics, the *A. ipaensis* genome is estimated to be ~1,475.83 Mb, which is consistent with the total scaffold length (Supplementary File 1: Table S6 and Figure S2). The average GC content is 36.70% (Table 1; Supplementary File 1: Figure S3), which is equivalent to that of the *A. duranensis* genome (Chen et al., 2016), and its distribution is highly similar to previously reported *Arachis* genomes (Bertioli et al., 2016; Chen et al., 2016) but different from those of *Glycine max, Arabidopsis thaliana*, and *Oryza sativa* (Supplementary File 1: Figure S4).

**Table 1:** Genome assembly and annotation of the *A. ipaensis*.

**Genome features**	**Measures**
**ASSEMBLY FEATURES**	
Number of scaffolds	79,408
Total span	1,391,700,926 bp (~1.39 G)
N50 (scaffolds)	170,050 bp
Longest scaffold	1,172,168 bp
Number of contigs	1,008,989
N50 (contigs)	8,067 bp
Longest contig	81,804 bp
GC content	36.70%
**GENE MODELS**	
Number of gene models	39,704
Mean transcript length	3,741 bp
Mean coding sequence length	1,246 bp
Mean number of exons per gene	4.99
Mean exon length	250 bp
Mean intron length	625 bp
Mean gene density	35.05 Kb
Number of genes annotated	39,645
Number of genes unannotated	59
**NON-PROTEIN CODING GENES/ELEMENTS**	
Number of pre-miRNA genes	71
Mean length of pre-miRNA genes	123 bp
Pre-miRNA genes share in genome	0.000590%
Number of pre-rRNA fragments	313
Mean length of pre-rRNA fragments	186 bp
Pre-rRNA fragments share in genome	0.003928%
Number of pre-tRNA genes	2,914
Mean length of pre-tRNA genes	75 bp
Pre-tRNA genes share in genome	0.014836%
Number of pre-snRNA genes	152
Mean length of pre-snRNA genes	111 bp
Pre-snRNA genes share in genome	0.001139%
Total transposable elements, bp (TEs)	1,125,924,736
Transposable element percent in genome	75.97%

**Figure 1:**
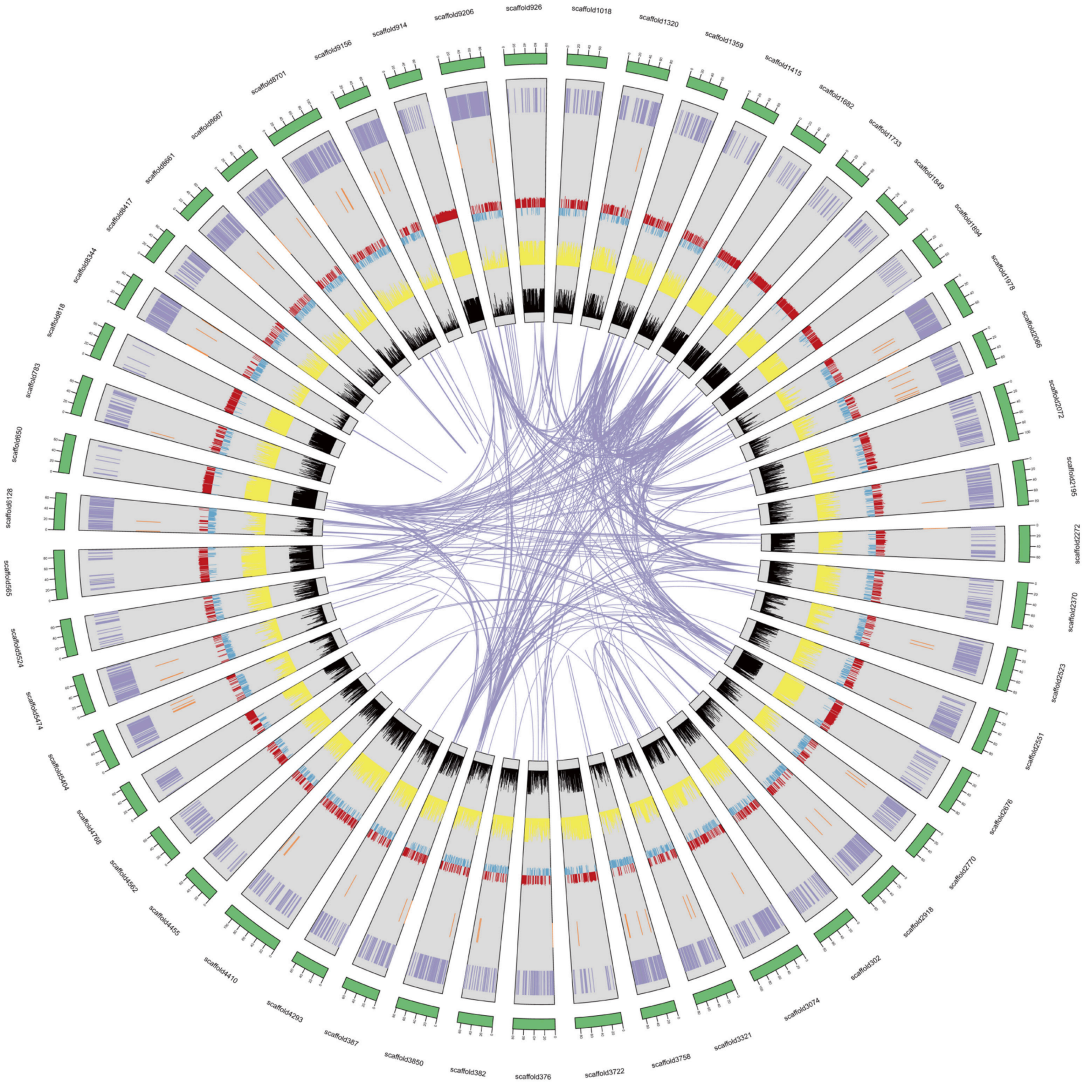
*A. ipaensis* genome overview. From the outer edge inward, circles represent the 50 largest DNA sequence scaffolds (green), the genes on each scaffold (purple), the non-coding RNA on each scaffolds (brown), GC content (red and blue), repeat density at 10 Kb (yellow), and transposable element density at 10 Kb (black).

We predicted 39,704 genes with average transcript and coding sequence lengths of 3,741 and 1,246 bp, respectively (Table 1). The whole-genome's gene density is one gene per 35.05 Kb (Figure 1 and Table 1), and the mean exon and intron lengths per gene are 250 and 625 bp (Table 1), respectively, which were relatively longer than those in other leguminous species, such as *Cicerarietinum* (Varshney et al., 2013) and *G. max* (Schmutz et al., 2010). Compared with the gene sets of legumes, oilseeds, and other plant species (Supplementary File 1: Table S7), the distribution of the *A. ipaensis* gene features is most similar to those of *A. duranensis* and legumes, such as *C. arietinum* and *G. max*, but different from those of non-leguminous species, such as *A. thaliana* and *O. sativa* (Supplementary File 1: Table S8 and Figure S5). Moreover, the *A. ipaensis* gene number is comparable to those of *Lotus japonicus* (39,366) and *Zea mays* (39,498), greater than that of *C. arietinum* (24,819), and substantially lower than those of *G. max* (54,174) and *Medicago truncatula* (50,444) (Supplementary File 1: Table S9). Functions were tentatively assigned to 39,645 genes but not to 59 genes that may be peanut-specific (Table 1). Most of the *A. ipaensis* genes have homologous gene models in the TrEMBL (99.82%) and Interpro (71.29%) databases (Bairoch and Apweiler, 2000; Zdobnov and Apweiler, 2001), and ~99.85% of the gene models matched entries in publically available databases (Supplementary File 1: Table S10). Conservative analyses indicated that the predicted proteins of *A. ipaensis* were most similar to those of *A. duranensis* (88.10%), followed by *Cajanus cajan* (67.4%), and least similar to those of gramineous crops, such as *Sorghum italica* (33.53%) and *S. bicolor* (34.51%) (Supplementary File 1: Table S8).

**Figure 2:**
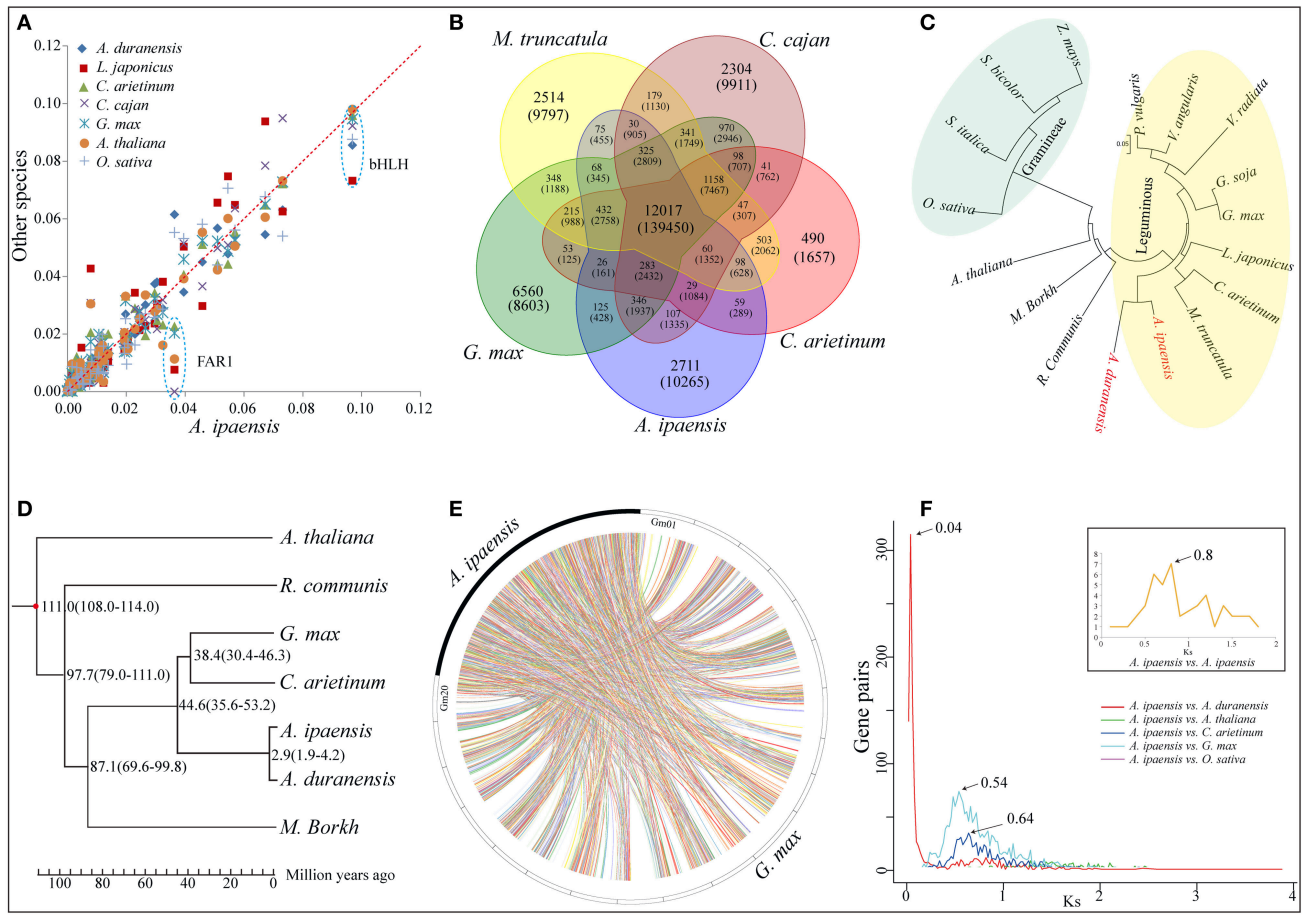
Comparative genomic and evolutionary analysis. **(A)** Scatter plot of percentage of *A. ipaensis* transcription factors in relation to *L. japonicas, C. arietinum, C. cajan, G. max, A. thaliana* and *O. sativa*. **(B)** Venn diagram showing distribution of gene families among *A. ipaensis, G. max, M. truncatula, C. cajan* and *C. arietinum*. **(C)** Cluster tree for 17 plant species including common leguminous and gramineous crops based on single copy orthologous genes. **(D)** Phylogenetic tree for 7 representative plant species. The numerical on each node represents the estimated differentiation time using the evolutionary time between *A. thaliana* and *G. max* (~108–114 Mya) as a correction. **(E)** Syntenic relationship between *A. ipaensis* scaffolds and *G. max* chromosomes. **(F)** Synonymous substitution rate (*Ks*) dating of duplication blocks in *A. ipaensis* and different combinations of orthologs of *A. duranensis, A. thaliana, C. arietinum, G. max*, and *O. sativa*. Different colored lines represent the distribution of *Ks* against orthologs gene pairs among different plant species. Inset shows the distribution of *Ks* between the gene pairs present in the duplicated blocks within the *A. ipaensis* genome.

A total of 2,530 putative *A. ipaensis* transcription factor (TF) genes were identified in 58 families, which was equal to or slightly higher than of the numbers found in *O. sativa* and *A. thaliana*, much higher than in *L. japonicus* but lower than in *G. max* and *Glycine soja* (Supplementary File 1: Figure S6). The distribution of the *A. ipaensis* TF genes among the families was highly similar to those of *A. duranensis* and *G. max* (Supplementary File 1: Figure S7). FAR1 was dominant in *A. ipaensis* (Figure 2A), as in the A-genome progenitor, *A. duranensis* (Chen et al., 2016). More importantly, the FAR1 TF families play pivotal roles in modulating phyA-signaling homeostasis (Lin et al., 2007), and phyA, together with phyB, regulate skotomorphogenesis and photomorphogenesis in higher plants (Medzihradszky et al., 2013). The FAR1 TF families identified in *A. thaliana* contained several conservative motifs (Supplementary File 1: Figure S8), and *phyA* and *phyB* were highly expressed in different tissues (shoot, seed, leaf, flower, and root) at different growth stages in *A. thaliana* (Supplementary File 1: Figure S9). In addition, previous non-synonymous substitutions per non-synonymous site (*Ka*)/synonymous substitutions per synonymous site (*Ks*) analyses of *phyB* in *A. duranensis* and *G. max* showed evidence of positive selection (Chen et al., 2016). These findings may enhance our understanding of peanut's unique fructification, having aerial flowers but subterranean fruit, as well as providing evidence for different regulators of biological functions in *Arachis* and other plants.

We identified 71 *Arachis* pre-microRNAs (pre-miRNAs) (Supplementary File 2: Data S1) with an average length of 123 bp, 2,914 pre-transfer RNAs (pre-tRNAs) with an average length of 75 bp, 313 pre-ribosomal RNAs (pre-rRNAs) with an average length of 186 bp including 5S (108), 5.8S (55), 18S (82), and 28S (68), and 152 pre-small nuclear RNAs (pre-snRNAs) with an average length of 111 bp. These genes represent 0.000590, 0.014836, 0.003928, and 0.001139% of the *A. ipaensis* genome, respectively (Table 1; Supplementary File 1: Table S11).

Approximately 75.97% of the *A. ipaensis* genome is composed of transposable elements (Figure 1; Tables 1, 2), which was higher than other legumes, such as *G. max* (59.00%) (Schmutz et al., 2010), *C. cajan* (51.60%) (Varshney et al., 2011) and *M. truncatula* (30.50%) (Young et al., 2011). Long-terminal repeat (LTR) retrotransposons are the dominant transposable elements, covering 64.15% of the nuclear genome (Table 2). Sequence divergence analyses indicated that most of *A. ipaensis* transposable elements had a ~30% divergence rate (Supplementary File 1: Figure S10).

**Table 2:** Organization of repetitive sequences in *A. ipaensis* genome.

**Repetitive elements**	**Repeat number**	**Length (bp)**	**In total repeat (%)**	**In genome (%)**
Total retrotransposons	2,444,183	9,88,193,900	87.77	66.68
LINE retrotransposons	163,947	43,942,874	3.9	2.97
SINE retrotransposons	2,859	726,676	0.06	0.05
LTR retrotransposons	2,277,377	950,690,158	84.44	64.15
Gypsy	1,727,232	796,763,491	70.77	53.76
Copia	343,066	91,500,532	8.13	6.17
LTR	23,529	1,543,961	0.14	0.10
Other	183,550	98,476,493	8.75	6.64
Other retrotransposons	668	47,680	0	0.00
Total DNA transposons	364,250	98,441,246	8.74	6.64
Total unclassified elements	311,209	84,709,729	7.52	5.72
Total transposable elements	3,120,310	1,125,924,736	–	75.97
Redundant		1,171,344,875		
Nonredundant		1,125,924,736		

The *A. ipaensis* genome contains 188,075 simple sequence repeats (SSRs), for which 80,218 SSR primers were designed (Supplementary File 1: Table S12; Supplementary File 3: Data S2). Of these SSRs, the di-nucleotide repeats are the most abundant, accounting for 48.38% of the total SSRs, followed by tri- nucleotide repeats (28.06%) (Supplementary File 1: Table S12). Among the di-nucleotide type, the AT/AT motif type had the greatest frequency (~21.9%). Among the tri-nucleotide type, the AAT/ATT is dominant (~11.4%) (Supplementary File 1: Figure S11). Using two A-genome genotypes (ICG_8123 and ICG_8138) and three B-genome genotypes (ICG_8960, ICG_8209, and ICG_13160) that were re-sequenced in our earlier study (Chen et al., 2016), we identified 26,050,150 variations, including 24,688,277 single nucleotide polymorphisms (SNPs) and 1,361,873 insertion-deletions (InDels) (Supplementary File 1: Table S13 and Figure S12). Among these variations, ~4 million SNPs were present in the two diploid A species (ICG_8123 and ICG_8138). By contrast, ~5 million SNPs were identified in the comparison of the three diploid B species (ICG_8960, ICG_8209, and ICG_13160) (Supplementary File 1: Table S13 and Figure S12). Thus, the diploid B species *Arachis magna* and *Arachis batizocoi* may have more abundant genetic diversity than the diploid A species *A. duranensis* when compared with the reference *A. ipaensis* (ICG_8206) genome assembly. The geographical origin of *Arachis* indicated that the distribution of *A. duranensis* is more extensive and also closer to that of *A. ipaensis* which has only one known location of origin, than *A. magna* (Bertioli et al., 2016). Another source of confusion among the variations may result from the two A-genome genotypes having fewer mapped reads than the three B-genome genotypes.

### Gene family and phylogenetic analysis

A total of 16,791 orthologous gene groups were identified among 18 species using OrthoMCL (Li et al., 2003; Supplementary File 4: Data S3), including 959 *A. ipaensis*-specific families containing 6,443 genes (Supplementary File 1: Table S14). A gene ontology (GO) annotation suggested differentially enriched functional categories in the peanut-specific families, indicating that these gene families may be closely related to the unique *Arachis* growth characteristics, such as aerial flowers but subterranean fruit, and lipid biosynthesis (Supplementary File 1: Figures S13–S15). Moreover, 1,624 of these orthologous groups were single-copy orthologs (Supplementary File 1: Table S15 and Figure S16). In addition, 6,443 unique paralogs of *A. ipaensis* genes occurred in species-specific homolog groups, indicating that these groups could arise from genomic structural rearrangements that obscured simple orthology, such as nonallelic recombination or gene conversion, followed by duplication (Supplementary File 1: Table S15 and Figure S16; Varshney et al., 2013). We identified 12,017 orthologous groups common to all five leguminous species (Figure 2B), 11,985 groups between *A. ipaensis* and *Ricinus communis* (oilseed crop) (Supplementary File 1: Figure S17), 9,099 groups between *A. ipaensis* and Gramineae/Poaceae crops (Supplementary File 1: Figure S18), and 10,501 orthologous groups are common to *A. ipaensis* and other distantly related plant species (Supplementary File 1: Figure S19). These results provide an important molecular foundation for comparative biology and for functional mechanistic inferences in *A. ipaensis*, as well as other species, because simple orthologous family genes often exhibit conserved molecular functions that were maintained during evolution process.

A polygenetic tree based on single-copy orthologous genes showed *A. ipaensis* and *A. duranensis* in the same clade, which did not include any other leguminous species, indicating their closer genetic distance and divergence time (Figure 2C). Furthermore, a special phylogenetic tree estimated that the divergence of the two species occurred ~2.9 million years ago (Mya) (Figure 2D), which was fairly consistent with a previous report (~2.16 Mya) (Bertioli et al., 2016). Syntenic blocks identified between *A. ipaensis* and other species was found to be extensively conserved (Supplementary File 1: Table S16). The largest number of syntenic blocks was identified between *A. ipaensis* and *G. max* (Figure 2E). The longest syntenic block (>10 Kb) was observed between *A. ipaensis* and *A. duranensis* (Supplementary File 1: Table S16). The numbers of syntenic blocks identified within the respective *A. ipaensis* and *A. duranensis* genomes were extremely lower than the number between the two genomes (Supplementary File 1: Figure S20) as well as the number between the *A. ipaensis* and *G. max* genomes (Supplementary File 1: Figure S21; Bertioli et al., 2016), indicating that few large-scale genome duplication events occurred in the *A. ipaensis* genome's evolution or that syntenic blocks were lost after genome duplication events.

The *Ks* values between paralogous or orthologous genes reveals a mechanism of molecular evolution (Lna, 1996). Distributions of *Ks* distances between paralogs within *A. ipaensis* and orthologs among *A. ipaensis*, leguminous crops and other species were plotted (Figure 2F). The *A. ipaensis* paralogs showed a peak at ~ 0.80, which is similar to those of *M. truncatula* (~0.80) and *L. japonicus* (~0.73) (Cannon et al., 2006) but lower than those of *A. duranensis* (~0.9) and *A. ipaensis* (~0.95) (Chen et al., 2016). Thus, the whole-genome duplication events of *A. duranensis* and *A. ipaensis* occurred around the time that corresponds to a *Ks* value range of 0.8–0.95. In addition, *A. duranensis* and *A. ipaensis* orthologs showed a prominent peak at ~0.04, which is consistent with a previous study (Bertioli et al., 2016). Assuming a synonymous substitution rate per synonymous site of 6.1 × 10^−9^ per year for eudicots (Lynch and Conery, 2000), the two species were estimated to have diverged ~3.28 Mya, which is close to the estimation based on the phylogenetic tree (Figure 2D). Furthermore, *Ks* dating suggested the divergence of *A. ipaensis* and *G. max* (*Ks* = ~0.54) at 44.3 Mya and that of *A. ipaensis* and *C. arietinum* (*Ks* = ~0.64) at 52.5 Mya.

The graphic trend of the *Ka*/*Ks* (ω) and *Ks* between the orthologs of *A. duranensis* and *A. ipaensis* formed three clusters, such as *Ks* = 0–0.3, 0.5–1.5, and >1.5, and the ω values decreased as the *Ks* values increased (Supplementary File 1: Figure S22). The genes with *Ks* ≥ 1.5 are attributed to pan-eudicot palaeoploidization, and the genes with lower ω ratios are considered to be under neutral selection. Here, the 45 *A. ipaensis* genes with ω ratios > 1 may be under positive selection pressure (Supplementary File 1: Figure S23).

Peanut is an allotetraploid species that may have originated from a single resent hybridization event between two diploid species, followed by polyploidization. Cytogenetic, phylogeographic and molecular evidence indicates that *A. duranensis* and *A. ipaensis* are the most likely donors of the A and B subgenomes, respectively (Kochert et al., 1996; Seijo et al., 2007; Robledo et al., 2009; Robledo and Seijo, 2010; Moretzsohn et al., 2013). A previous study estimated the divergence of the two species at ~2.88 Mya (Moretzsohn et al., 2013). The estimation using a comparative genomics analyses between them was ~2.9 Mya, which was fairly consistent with our report. Moreover, sequence comparisons with tetraploid cultivated peanut estimated the divergence times of *A. duranensis* and *A. ipaensis* from the A and B subgenomes of *A. hypogaea* as ~247,000 and ~9,400 years, respectively (Bertioli et al., 2016).

Comparative genomics analyses of chromosomal structure and synteny between *A. duranensis* and *A. ipaensis* indicated that some chromosomes shared a conservative linear structure that was partially in accordance with our other analyses (Supplementary File 1: Figure S20). Other analyses showed a large inversion in one or both arms of a chromosome (Bertioli et al., 2016). In contrast, chromosomes 07 and 08 have undergone complex rearrangements that were consistent with cytogenetic observations (Seijo et al., 2007; Nielen et al., 2010). Importantly, a genomic comparison showed a fundamentally one-to-one correspondence between the diploid chromosomes and cultivated peanut linkage groups. However, the *A. duranensis* chromosomes were less similar to *A. hypogaea* sequences than those of *A. ipaensis* (Bertioli et al., 2016). These results may help to uncover potential mechanisms of hybridization events in the future.

### Disease resistances and nucleotide-binding site (NBS)-leucine-rich repeat (LRR) encoding genes

Plant NBS-LRR proteins encoded by resistance genes (*R* genes) play key roles in the responses of plants to various pathogens. The *R* genes can be classified into various subfamilies based on the present of different domain, such as CC-NB-LRR, TIR-NB-LRR, ser/thr-LRR, Kin-LRR, and others (e.g., *Mol* and *Asc-1*; Sanseverino et al., 2010). The *A. ipaensis* genomic assembly contains 1,437 putative disease *R* genes as assessed by a screening of the PRG database (Supplementary File 1: Table S17; Supplementary File 5: Data S4; Sanseverino et al., 2010). Compared with other legumes, the *A. ipaensis* genome possesses more *R* genes than the *G. max* and *M. truncatula* genomes but less than the *A. duranensis* and *C. cajan* genomes. Moreover, these *R* genes tend to cluster on different scaffolds. For example, several large clusters containing 6–10 *R* genes are located on six different scaffolds (Supplementary File 1: Figure S24). The NL subfamily of genes, which confers resistance against pests and diseases, is the second largest *R* gene-containing family, and these genes can be clustered reasonably into different individual clades in *A. ipaensis, A. duranensis*, and *A. thaliana*, indicating that gene divergence occurred during the evolution of the three species (Supplementary File 1: Figure S25). In addition, we analyzed protein motifs in the most homology of the top 20 *R* genes found in PRG database using MEME (Bailey et al., 2009), and the results showed highly conserved LRR motifs (Supplementary File 1: Figure S26). However, further investigation is required to determine the biological functions of these *R* genes.

### Identification of genes related to biological nitrogen fixation

**Figure 3:**
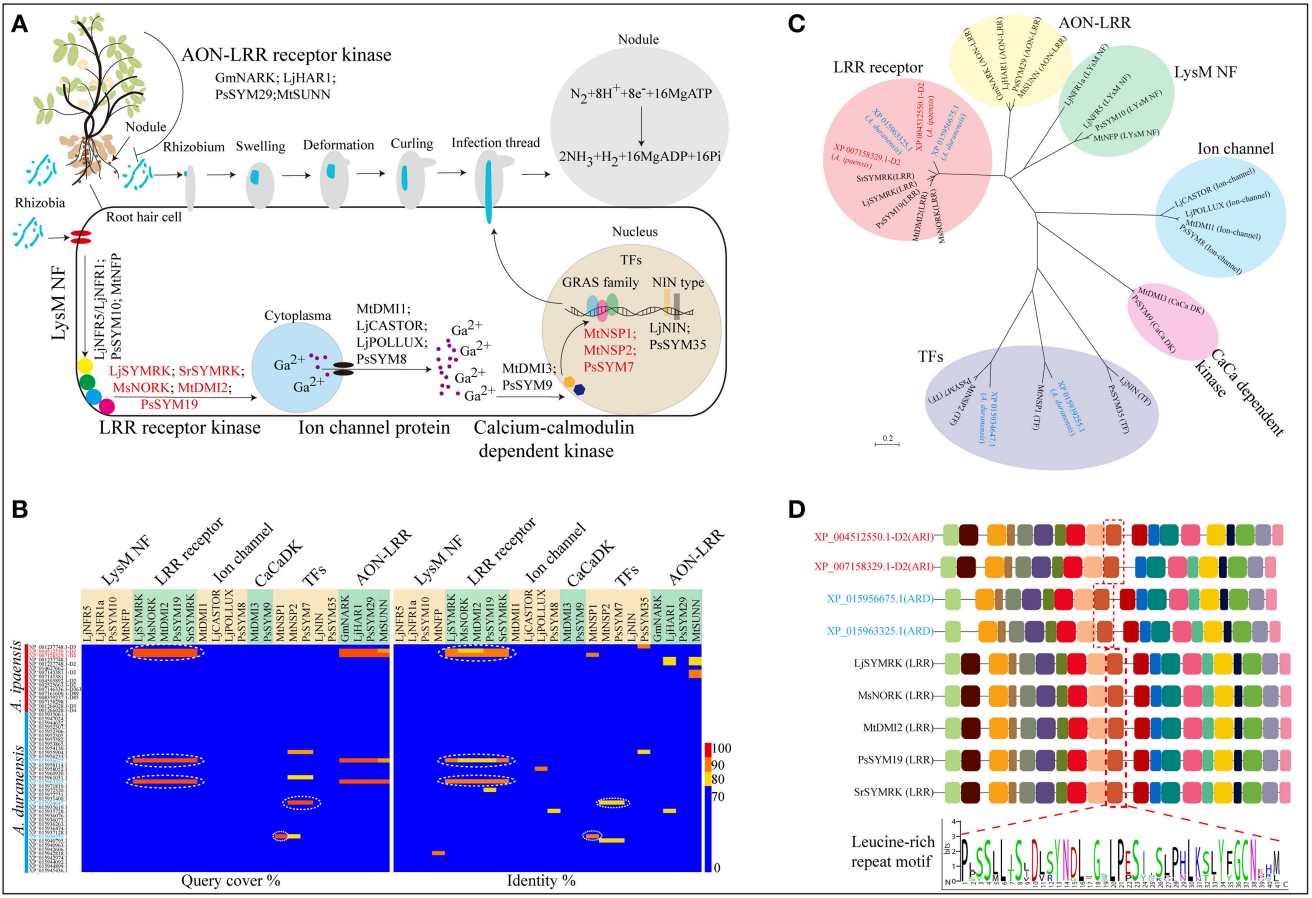
Biological nitrogen fixation in leguminous plants. **(A)** Genes involved in nodule initiation, development and signal recognition pathway. **(B)** Protein sequence alignment of Nod related genes identified in *A. ipaensis* and *A. duranensis*. **(C)** Phylogenetic tree of nodule development genes and their homologs from *A. ipaensis* and *A. duranensis*. **(D)** Identification of high conserved domains of leucine-rich repeat (LRR) receptor kinases. Red dashed boxes represent LRR conserved motif. **(A)** The rhizobium (blue) attach to the surface of root hair cell. After swelling, deformation, curling and infection thread, the bacteria are released into cells via endocytosis then a vacuole-like structures (symbiosomes), in which the bacteria convert N_2_ to NH_3_, formed. But how is the Nod signal transmitted? Initially, the rhizobia-derived signal is perceived by LysM-type protein receptor kinases, such as NRF1 and 5 (Radutoiu et al., 2003) and SYM10 (Schneider et al., 2002) identified in *L. japonicus* and *P. sativuml*, followed by a downstream leucine-rich receptor kinase, for example SYMRK (Stracke et al., 2002 and Capoen et al., 2005), NORK (Endre et al., 2002), DMI2 (Catoira et al., 2000), and SYM19 (Stracke et al., 2004) from *L. japonicus, Sesbania rostrata, M. sativa, M. truncatula*, and *P. sativuml*, respectively. Then, the Nod factor (NF) signal is processed through a signal transduction cascade involving proteins including ion channels [MDI1(Ané et al., 2004), CASTOR (Imaizumi-Anraku et al., 2005), POLLUX (Imaizumi-Anraku et al., 2005), and SYM8 (Edwards et al., 2007)], calcium-calmodulin-dependent kinase (CaCaDK) (MDI3 and SYM9) (Lévy et al., 2004) and transcription factors [NSP1 (Smit et al., 2005), NSP2 (Kaló et al., 2005), SYM7 (Kaló et al., 2005), NIN (Schauser et al., 1999), and SYM35 (Borisov et al., 2003)]. Finally, rhizobia infection occurred primarily through uncharacterized target genes that may be activated by these TFs.

Nitrogen is one of the most important plants require nutrients, and in agriculture nitrogen availability influences both crop yield and quality. Leguminous plants, such as peanut, soybean, and *Medicago*, can transform molecular nitrogen into available ammonia nitrogen through the leguminous-root-nodule bacteria nitrogen-fixing system that results from the symbiotic interactions between leguminous plants and rhizobia (Figure 3A). In the *A. ipaensis* and *A. duranensis* genomic assemblies, 16 and 38 root-nodule developmental genes respectively, have been identified (Supplementary File 1: Table S18; Supplementary File 6: Data S5). As expected, there are greater numbers of nodulation-related genes present in leguminous plants than in non-leguminous plants, such as *A. thaliana, O. sativa*, and *Z. mays* (Supplementary File 1: Figure S27).

Nitrogen-fixing root nodules are important symbiotic organs that provide an epiphytic site for rhizobia to convert atmospheric nitrogen to ammonia, and supply its host plant with fixed nitrogen. In return, the rhizobia gain photosynthates from the plant (Figure 3A). In leguminous plants, multiple genes are involved in the formation and development of root nodules, as well as in the autoregulation of the nodulation (AON) process, which is a systemic feedback loop used to avoid an excessive energy expenditure from “unwanted” nodulation (Figure 3A; Supplementary File 7: Data S6). Here, four homologous LRR receptor kinase genes were identified in *A. ipaensis* (XP_004512550.1-D2 and XP_007158329.1-D2) and *A. duranensis* (XP_015956675.1 and XP_015963325.1) (Figure 3B; Supplementary File 1: Figure S28; Supplementary File 7: Data S6). A phylogenetic tree showed that the four homologous genes were clustered into an independent clade, together with other LRR receptor kinase genes (Figure 3C). Interestingly, these four genes contain multiple common motifs, including a conserved LRR motif, indicating a similar biological function (Figure 3D). The GO analyses suggested that the four homologous genes are involved in ion binding and signal transducer activity (Supplementary File 1: Figures S29–S32). More importantly, the proteins encoded by the four genes showed similar three-dimensional structures and localized on the cell membrane (Supplementary File 1: Figures S29–S32).

We also identified two other nodule development-related genes (XP_015934647.1 and XP_015939255.1) that are homologous to the TF genes of the GRAS family in *A. duranensis*. One gene is homologous with *MtNSP2* and *PsSYM7* (Kaló et al., 2005), while the other is an ortholog of *MtNSP1* (Imaizumi-Anraku et al., 2005) (Figures 3A,B; Supplementary File 1: Figure S33; Supplementary File 7: Data S6). The phylogenetic tree indicated that the two homologs were classified into the TF category but appeared in different branches (Figure 3C). In addition, the GO enrichment indicated that the two genes participate in the regulation of multiple biological processes, such as nucleic acid-binding TF and signal transducer activities (Supplementary File 1: Figures S34, S35). The three-dimensional structures of the two proteins were completely dissimilarity, and the two proteins localized in the nucleus (Supplementary File 1: Figures S34, S35). These results could provide candidate genes and basic bioinformation for further functional studies of nodule formation in leguminous crops.

### Genetic mechanism of drought adaptation

Peanut (*A. hypogaea* L.) is a typical upland crop in tropical, subtropical, and warm temperate climates. Drought adaptation plays a central role in their growth and development. During drought stress, TFs, such as MYB, MYB-related, NAC, WRKY, bZIP, and ERF, are involved in numerous physiological responses (Shinozaki and Yamaguchi-Shinozaki, 2000) (Supplementary File 1: Figure S36). Here, the total number of TF genes identified in upland crops was greater than that found in hygrophilous plants (Supplementary File 1: Table S19 and Figure S37). Notably, in *A. ipaensis* we identified 185 MYB and 129 MYB-related TFs (Supplementary File 1: Table S19), most of which contain a highly conserved DNA-binding domain, and they are key factors in regulatory networks controlling development, metabolism and responses to biotic and abiotic stresses (Dubos et al., 2010). The second large number of drought tolerance-related TFs, with 170 members, is the ERF family (Supplementary File 1: Table S19). The ERF proteins, sharing a conserved 58–59 amino-acid domain, are key regulators linked to responses to plant stresses, such as cold, drought and pathogen attack (Supplementary File 1: Figure S38; Singh et al., 2002). In *A. duranensis, A. ipaensis* and *A. hypogaea* species, sets of 51, 57 and 53 ERF TF family proteins, respectively, were obtained from the Plant Transcription Factor Database (Jin et al., 2015, 2017; Supplementary File 1: Figures S39–S41). These TF proteins contained different DNA-binding domains and can be categorized into different branches based on different motif permutation structures, indicating the distinct functional and evolutionary features of ERF TFs in different *Arachis* species (Supplementary File 1: Figures S39–S41).

Heat-shock proteins (Hsps)/chaperones are important defense mechanism members against abiotic stresses, such as drought, salinity and extreme temperatures (Wang et al., 2004; Supplementary File 1: Figure S42). Drought stress is a common factors that induces Hsp expression (Kimpel et al., 1990; Sun et al., 2002). To elucidate the cause of drought tolerance, five major families of Hsps/chaperones were identified in upland crops and hygrophilous plants (Supplementary File 1: Table S20 and Figure S43). As expected, the total number of Hsps/chaperones obtained in upland crops was much great than in hygrophilous plants (Supplementary File 1: Figure S43).In particular, *A. ipaensis* and *G. soja* had 118 and 34 Hsp70 subfamilies, respectively, compared with only 1 in rice (Supplementary File 1: Table S20). The great number of Hsps/chaperones detected in *A. ipaensis* and *G. soja* indicates the nature of drought adaptation in upland crops.

The subtilisin-like protease (SDD1) gene family is involved in the regulation of stomatal density and distribution to adjust for drought stress by modulating the apertures of these pores flanked by two guard cells (Berger and Altmann, 2000). In the expanded gene families, 39 and 40 SDD1 genes were identified in *A. ipaensis* and *A. duranensis*, respectively (Supplementary File 8: Data S7). These gene families were divided into different clusters according to their related functions but showed a pattern of cross-distribution in each cluster based on their different genetic relationships (Supplementary File 1: Figure S44).

### Oil synthesis

Because peanut is an important oilseed crop, 1,613 *A. ipaensis* genes related to the biosynthesis of fatty acids and triacylglycerols were identified, which was more than were identified in the nonoilseed plant *Arabidopsis* (1,380) and rice (1,419) (Supplementary File 1: Table S21). In addition, fatty acids and triacylglycerols synthesis involves many key enzymes, such as ACCase (Slabas and Fawcett, 1992), acyl-ACP thioesterase (A and B) (Dörmann et al., 2000; Bonaventure et al., 2003; Serrano-Vega et al., 2005), LACS (Zhao et al., 2010), DGAT (Yen et al., 2008), and FAD (Pham et al., 2012) (Supplementary File 1: Figure S45). When we manually investigated the homologous genes in the storage lipid biosynthesis pathway using the Arabidopsis Lipid Gene Database (Mekhedov) (http://lipids.plantbiology.msu.edu/), 116 nonredundant homologs potentially involve in lipid biosynthesis were obtained in *A. ipaensis* (Supplementary File 9: Data S8). Consistent with the lipids produced in peanut seeds, one, and nine homologous genes encoding acyl-ACP thioesterase A and B (*FATA* and *FATB*), respectively, the two key enzymes leading to the synthesis of fatty acid, were identified. Moreover, multiple copies or isoforms of some key genes, such as *FAD2, LACS*, and *KAS*, involved in triacylglycerol synthesis were also detected in the *A. ipaensis* genome (Supplementary File 9: Data S8).

**Figure 4:**
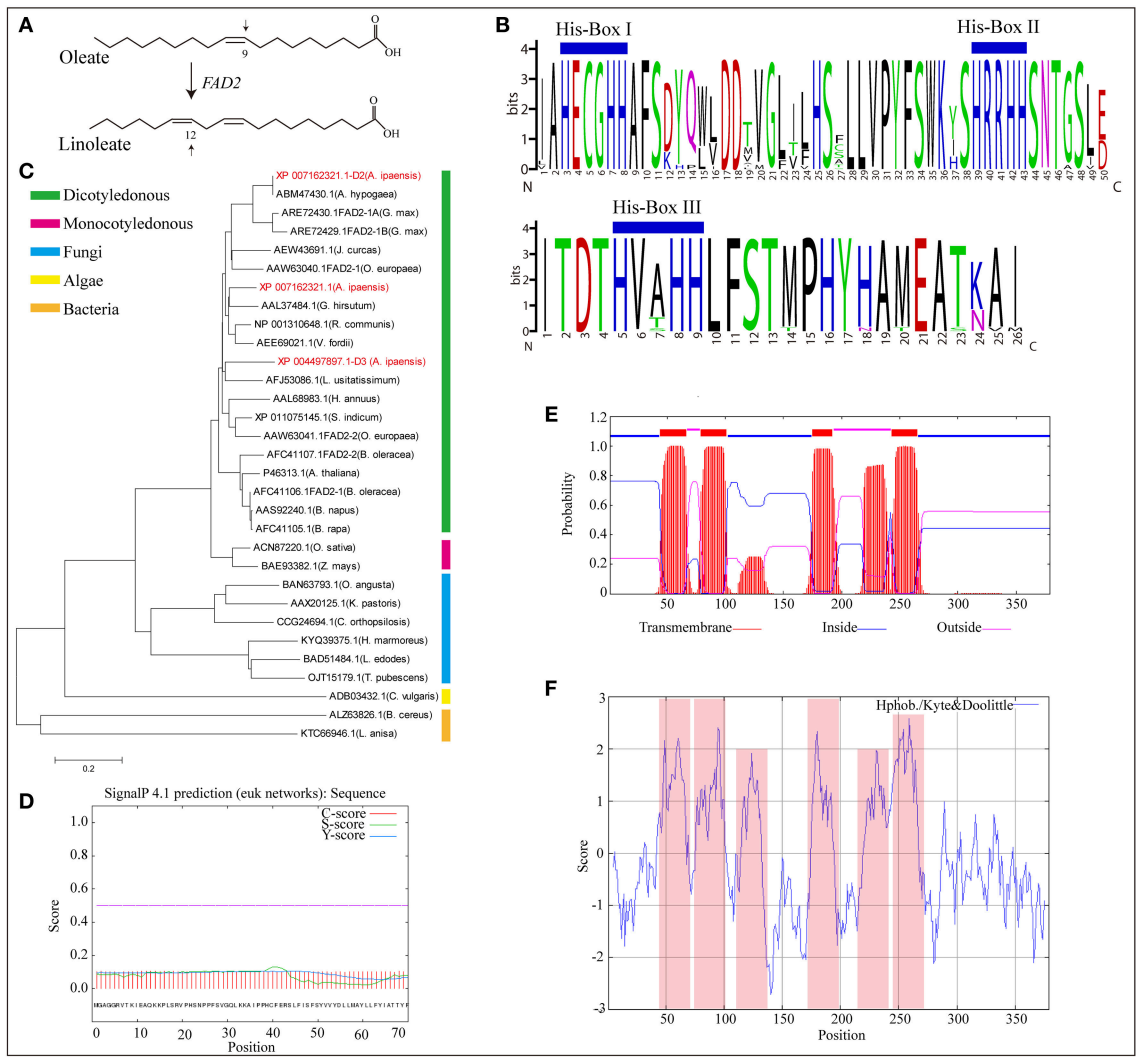
Homologous genes of δ-12 oleic acid desaturase (*FAD2*). **(A)** *FAD2* catalyze oleate into linoleate. **(B)** Multiple alignment of amino acid sequence of substrate binding motif of FAD2 in oil seed plants and its homologous genes in *A. ipaensis*. **(C)** Phylogenetic tree of FAD2 and its homologous genes from different species. **(D)** Signal peptides analysis of FAD2 homologous gene (XP_007162321.1-D2) from *A. ipaensis*. **(E)** Tansmembrane region prediction of FAD2 homologous gene, XP_007162321.1-D2. Red, blue, and pink boxes represent transmembrane, inside, and outside domains. **(F)** Hydrophobicity and hydrophilicity prediction for the homologous gene XP_007162321.1-D2. Pink box represent protein hydrophobic region.

*FAD2*, encoding δ-12 oleic acid desaturase, is the essential gene that controls linoleic acid biosynthesis (López et al., 2000). It converts oleic acid to linoleic acid by desaturating the δ-12 carbon and determines the multi-polyunsaturated fatty acid content and proportion in most oil seed plants (Figure 4A). In this study, three new *FAD2* homologous genes (XP_004497897.1-D3, XP_007162321.1, and XP_007162321.1-D2) were identified in *A. ipaensis* (Supplementary File 1: Figure S46). The proteins of *FAD2* and its homologs contain the highly conserved feature of three enzyme-specific histidine boxes (Figure 4B), which are considered to be essential for desaturase activity because they act as potential ligands for iron atoms (Sakai and Kajiwara, 2005). A phylogenetic tree showed that *FAD2* clustered into five groups based on its genus, and the three homologous genes were more closely related to the evolutionary kinship of oil seed plants, especially *A. hypogaea* (Figure 4C). This result indicated that *FAD2* is an extremely conserved gene in the fatty acid biosynthesis pathway. In addition, the GO terms revealed that the three homologous genes having δ-12 oleic acid dehydrogenase activities (ω-6 fatty acid desaturase activities) were involved in the fatty acid biosynthesis process and that the proteins encoded by the three genes were subcomponents of the endoplasmic reticulum membrane. They had similarity three-dimensional structures, which was supported by the predicted protein subcellular localization (Supplementary File 1: Figures S47–S49).

Pairwise comparisons of the amino acid sequences of XP_007162321.1-D2 from *A. ipaensis* with FAD2 from *A. hypogaea* revealed 100% sequence identities with no gaps (Supplementary File 1: Figure S50), which confirmed the ancestral origin of *FAD2* as being the *A. ipaensis* genome. A signal peptide analysis showed a low level S-score, indicating a typical non-secretory protein with no leading peptide (Figure 4D). This was supported by the predicted protein subcellular localization (Supplementary File 1: Figure S47E). Moreover, four transmembrane domains were predicted in their amino acid sequence (Figure 4E). Importantly, the protein hydrophobicity/hydrophilicity prediction revealed four strong hydrophobic regions, which completely overlapped with the transmembrane regions (Figure 4F). These results provide information for exploring the origin of *FAD2*, and the homologous gene will be of service to peanut improvement for high oleic acid.

Among the key enzyme-encoding genes, 82 nonredundant homologous genes had high distributions of non-synonymous substitutions (*Ka*/*Ks* > 1.0) between *A. ipaensis* and *A. thaliana* as assessed by the branch-site likelihood ratio test, indicating positive selection during domestication (Supplementary File 1: Figure S51; Supplementary File 10: Data S9). Coincidentally, 21 fatty acid biosynthesis genes located in multiple improvement-selective sweeps regions were obtained through combined genome selective sweeps and GWAS analyses in soybean (Zhou et al., 2015). Thus, we hypothesize that these 82 genes, including *FAD2* (2), *KASIII* (2), and *FATB* (6) homologs with high *Ka*/*Ks* values (Supplementary File 1: Figure S52) may also have undergone domestication.

TFs that regulate seed development play crucial roles in seed lipid accumulation. To date, the TFs regulating lipid metabolism mainly belong to the following 6 super gene families, AP2/EREBP, B3, NF-Y, Dof, MYB, and MYC (Song et al., 2016). The number of the TF families identified in oilseed crops is much great than in non-oilseed plants (Supplementary File 1: Figure S53). Information related to these genes involved in fatty acid and triacylglycerol metabolic pathways is important for modifying the oil quality of peanut as well as other oilseed crops.

## Conclusions

The draft genome sequence of *A. ipaensis*, together with those of *L. japonicus, M. truncatula, C. cajan, C. arietinum*, and *G. max*, will provide new biological information for an important branch of the legume clade. The *A. ipaensis* genome sequence presented here, combined with our previous sequence of *A. duranensis*, will shed light on the genomic evolution and polyploidization mechanisms of polyploid species. In addition, the biological information of the *A. ipaensis* genome provides a fundamental resource for understanding disease resistance, symbiotic nitrogen fixation, environmental adaptation and oil biosynthesis in peanut. Moreover, high-density molecular markers, such as SSRs and SNPs, identified in the *A. ipaensis* draft genome can be used to investigate the genetic diversity and make genetic changes to improve important agronomic traits in peanut.

## Materials and methods

### Plant material

The *Arachis* genus is composed mostly of diploid species (2n = 2x = 20). Peanut (*A. hypogaea* L.) is an allotetraploid (AABB-type genome; 2*n* = 4*x* = 40), probably derived from a single recent hybridization event between *A. duranensis* (AA subgenome, 2n = 2x = 20) and *A. ipaensis* (BB subgenome, 2n = 2x = 20) (Supplementary File 1: Figure S1; Koppolu et al., 2010; Chen et al., 2016). In 2016, an accession of *A. ipaensis* K30076 has already been sequenced (Bertioli et al., 2016). The accession collected by A. Krapovickas, W.C. Gregory, D.J. Banks, J.R. Pietrarelli, A. Schinini, and C.E. Simpson in 1977 was maintained at Embrapa Genetic Resources and Biotechnology (Brasília, Brazil), which probably originated form Villa Montes near Camatindi or Tigüipa, Bolivia (https://www.peanutbase.org/; Bertioli et al., 2016). In this study, the accession of *A. ipaensis* ICG_8206 maintained at International Centre for Research in the Semi-Arid Tropics (India) then introduced to Crops Research Institute-Guangdong Academy of Agricultural Sciences (China) was used. Although cytogenetic, phylogeographic and molecular evidence showed that the accession of *A. ipaensis* K30076 was the most probable B-genome donor for *A. hypogaea* (Seijo et al., 2007; Robledo and Seijo, 2010; Bertioli et al., 2016), genetic relationship analyses indicated that the B-genome accession ICG 8206 (*A. ipaensis*) was the most closely related to *A. hypogaea* (Koppolu et al., 2010).

Here, the *A. ipaensis* (ICG_8206) was sequenced by Illumina HiSeq2500 platform. Total genomic DNA was isolated from the etiolated unopened young leaves of 20-day-old plants cultivated in dark chamber according to a modified CTAB procedure (Doyle and Doyle, 1990). This work will also be of great importance to guide cultivated peanut's genome assembly as a necessary complement in future.

### Whole-genome shotgun sequencing and *de novo* assembly

Whole-genome shotgun sequencing was performed under the HiSeq2500 Sequencing System with 11 paired-end sequencing libraries, including 250, 500, 800 bp, 2, 5, 10, and 20 Kb using the standard protocol provided by Illumina (San Diego, USA).

SOAPdenovo2 (version 2.04.4) (Luo et al., 2012) was employed with optimized parameters to construct contigs and original scaffolds as previous described (Chen et al., 2016). Subsequently, SSPACE (version 2.0) (Boetzer et al., 2011) was used to link the scaffolds constructed by the SOAPdenovo2 as previous described (Chen et al., 2016).

The genome size was estimated based on the 17 *K*-mer distribution using the total length of sequence reads divided by sequencing depth, and the frequency of each 17-mer were calculated from the whole genome sequenced reads to evaluate the sequencing depth. Subsequently, the *A. ipaensis* genome size was calculated by following the algorithm: Genome size = *K*-mer number/Peak depth (Bertioli et al., 2016).

The gene coverage of the assembled genome was comprehensively evaluated using available public transcript sequence tags or expressed sequence tags. Core eukaryotic genes identified by CEGMA v.2.3 (Parra et al., 2007) were remapped to the *A. ipaensis* genome assembly by BLAT (Kent, 2002) to evaluate the quality of the assembly. CEGMA data were downloaded from the Korf Lab research group at the Genome Center, UC Davis (http://korflab.ucdavis.edu/datasets/cegma/#SCT6).

### Gene prediction and function annotation

To annotate the *A. ipaensis* genome, an automated genome annotation pipeline MAKER was performed to produce *de novo* gene prediction, infer 5′ and 3′ UTR, and integrate these data to generate final downstream gene models with quality control statistics (Cantarel et al., 2008). All predicted genes were functionally annotated as previous described (Chen et al., 2016). The annotation was conducted using the BLAST+ (version 2.2.27) with 1e-5 as the E-value threshold to against the SwissProt and TrEMBL databases (Bairoch and Apweiler, 2000). To infer functions for the predicted genes, InterProScan (version 4.7) (Zdobnov and Apweiler, 2001) was used to search the predicted genes against the protein signature from InterPro with default parameters. All genes were also aligned against to the Kyoto Encyclopedia of Genes and Genomes (KEGG) pathway (Kanehisa et al., 2004).

In order to evaluate the conservation of *A. ipaensis* ICG_8206 gene model, the BLASTP was used to query the *A. ipaensis* ICG_8206 proteome against the proteomes of other plant species (Supplementary File 1: Table S7) with an E value of 1e-10 as cut-off (Supplementary File 1: Table S8).

### Gene family analysis

All the predicted gene models were analyzed using OrthoMCL (Li et al., 2003) to identify shared and specific gene families among 17 species (Supplementary File 1: Table S7). In the first step, inter and intra species BLASTP with an E-value cutoff of 1e-10 was performed to detect reciprocal best hit pairs between species (putative orthologs), as well as sets of genes within species (putative co-orthologs or in-paralogs). The reciprocal best hit matrix served as the basis for ortholog definition using OrthoMCL. Subsequently, orthologous groups were organized into species-specific and higher taxonomic level groups by requiring that at least one sequence from each clade under comparison be present in the intersecting set. Finally, based on fourfold degenerate sites of single-copy ortholog genes in all species, a phylogenetic tree was constructed using MEGA v6.0 (Tamura et al., 2013) and PhyML v3.0 (Guindon et al., 2010).

To identify TFs in *A. ipaensis*, the PlantTFDB database was used to search TFs in other plant species (http://planttfdb.cbi.pku.edu.cn/). The predicted genes were used to BLAST search against the PlantTFDB (E-value: 1e-10). The FAR1 motif was predicted using the Multiple Expectation Maximization for Motif Elicitation (MEME)/Motif Alignment and Search Tool (MAST) system (http://meme-suite.org/) (Bailey et al., 2009) and visualized using the TBtools (version 0.4999) (https://github.com/CJ-Chen/TBtools).

### Non-coding RNAs and repetitive sequence annotation

Non-coding RNAs were predicted by aligned *A. ipaensis* genome assembly to against the Rfam databese (version 12.1) (Nawrocki et al., 2015). The pre-tRNAs were identified using tRNAscan-SE (Lowe and Eddy, 1997), pre-rRNAs were predicted using RNAmmer (Lagesen et al., 2007), pre-snRNAs were annotated using INFERNAL (Nawrocki et al., 2009) and others were also obtained by BLAST search against the Rfam database.

The RepeatMasker (Chen, 2004), RepeatProteinMask (http://repeatmasker.org/), Tendem Repeats Finder (TRF) (Benson, 1999) and RepeatModeler (Smith and Hubley, 2014) were performed to identify repetitive sequences through homolog and *de novo* prediction. The RepeatMasker and RepeatProteinMask were used to screen the *A. ipaensis* genome against the RepBase database (http://www.girinst.org/). The transposable elements (TEs) were classified as described without consideration of the gaps in the genome assembly (Wicker et al., 2007).

### Identification of SSRs and SNPs

MIcroSAtellite (http://pgrc.ipk-gatersleben.de/misa/) was used to mine SSRs in *A. ipaensis* genome, and primer 3 v3.0 was used for primer design (Thiel et al., 2003; Untergasser et al., 2012). A SSR was defined with at least 6 repeats for di-nucleotide motifs or 4 repeats for tri-, tetra-, penta-, and hexa-nucleotide motifs. The maximum number of interrupting nucleotides in a compound SSR was set as 100.

Reads from five re-sequenced genotypes including two A-genome genotypes (ICG_8123 and ICG_8138) and three B-genome genotypes (ICG_8960, ICG_8209, and ICG_13160) were used to identify genome SNP and InDel variations (Chen et al., 2016). Total of these sequenced reads were aligned to the reference genome (ICG_8026) using the Burrows Wheeler Aligner program (BWA) (Li and Durbin, 2009). Subsequently, SNPs and InDels were identified using GATK v3.5 (http://www.broadinstitute.org/gatk) with default parameters, respectively.

### Evolutionary and syntenic block analyses

The phylogenetic tree was constructed based on single-copy orthologous genes shared in *A. ipaensis* and other 17 plants (Supplementary File 1: Table S7) using MEGA v6.0 with the maximum-liklihood algorithm (Tamura et al., 2013).

Syntenic blocks between the genomes of *A. ipaensis* and other plants were identified using the MCScanX with default parameters (Wang et al., 2012) and visualized on the genome using Circos (Krzywinski et al., 2009). Genomic sequences were first aligned annotated genes based on amino acid sequence using Promer package of Mummer (version 3.22) (Delcher et al., 2002). Whole genome dot plots were generated using Mummerplot (Delcher et al., 2002) and Gunplot 5.0 (www.gnuplot.info/). *K*s values of the homologs within collinearity blocks were calculated using the perl script, add_ka_and_ks_to_collinearity.pl included in MCScanX package, and the median of *K*s values was considered to be a representative of the collinearity blocks.

### Genes involved in disease resistance, symbiotic nitrogen fixation, environmental adaptation, and oil synthesis

All the disease R genes were identified using the genome assembly of *A. ipaensis* and other plant species as a TBLASTN query to against the PRG datebase with an E-value of 1e-10 as cut-off. Amino acid sequences of all NBS-LRR genes from *A. ipaensis, A. duranensis*, and *A. thaliana* were aligned to construct phylogenetic tree using MEGA v6.0 with automatic bootstrap criteria (Maximum Likelihood) (Tamura et al., 2013). The conserved motifs of top 20 homologies NBS-LRR were identified using MEME suite (Bailey et al., 2009; Supplementary File 1: Figure S26).

Nodulation regulatory and nodulin genes were identified based on GO analyses. The GO IDs for each gene were obtained through BLAST search against KEGG proteins (E-value: 1e-5). Genes involved in symbiotic nitrogen fixation associated with nodule development and AON process were obtained by comparison with orthologous genes in other legumes using multiple protein sequence alignment in COBALT (https://www.ncbi.nlm.nih.gov/tools/cobalt/). The PredictProtein was used to perform GO terms, protein-protein and protein-DNA binding sites and sub-cellular localization (Yachdav et al., 2014). The SWISS-MODEL was used to predict protein tertiary structure (Biasini et al., 2014).

Genes involved in oil biosynthesis for *Arabidopsis* were obtained from the Arabidopsis Lipid Gene Database (Mekhedov) (http://lipids.plantbiology.msu.edu/). All the *Arabidopsis* lipid genes (81) in the database were used to TBLASTN search against the *A. ipaensis* genome with a cutoff E-value of 1e-50. Finally, a total of 116 non-redundant oil biosynthesis genes were obtained in *A. ipaensis*. Multiple amino acid sequence alignment of *FAD2* homologs was performed using the COBALT (https://www.ncbi.nlm.nih.gov/tools/cobalt/). The PredictProtein and SWISS-MODEL was used to integrate GO terms, protein binding sites, sub-cellular localization and protein tertiary structure, respectively (Biasini et al., 2014; Yachdav et al., 2014).

Signal peptide analysis of the XP_007162321.1-D2 was predicted using SignalP 4.1 Server with default parameter (Petersen et al., 2011). Prediction of transmembrane helices was performed using TMHMM Server v. 2.0 (http://www.cbs.dtu.dk/services/TMHMM/). Hydrophobicity and hydrophilicity regions were predicted using ProtScale (Gasteiger et al., 2005).

## Author contributions

XQL, and XC designed the experiments and managed the project. QL, HFL, and YH performed the research. QL, SW, XYL, GYZ, SL, HL, and HYL analyzed the data. QL wrote the manuscript with the help of GQZ, ZL, and RV. All authors read and approved the final manuscript.

### Conflict of interest statement

The authors declare that the research was conducted in the absence of any commercial or financial relationships that could be construed as a potential conflict of interest.

## Supplementary material

The genome assembly and annotation data were deposited in the DRYAD digital repository (https://datadryad.org/). All the data can be downloaded from DRYAD by searching for doi: 10.5061/dryad.hm5vs13 or by directly accessing the link: https://doi.org/10.5061/dryad.hm5vs13.

Supplementary File 1 Supplementary **Tables S1–S21** and **Figures S1–S53**.

Supplementary File 2: Data S1 Details on the 71 miRNA identified in *A. ipaensis*.

Supplementary File 3: Data S2 Summary of primer sequences for SSR markers.

Supplementary File 4: Data S3 Summary of orthologous gene groups.

Supplementary File 5: Data S4 Detils on the 1,437 putative disease resistance genes in *A. ipaensis*.

Supplementary File 6: Data S5 Summary of 16 nodulin and nodulation associated genes in *A. ipaensis*.

Supplementary File 7: Data S6 Summary of multiple genes associated with nodulation development and nodule autoregulation (AON) signal pathway.

Supplementary File 8: Data S7 Summary of 39 subtilisin-like protease (SDD1) genes in *A. ipaensis*.

Supplementary File 9: Data S8 List of *A. ipaensis* genes orthologous to encoding key enzymes in the lipid biosynthesis pathways.

Supplementary File 10: Data S9 Details on the 82 oil sythesis genes with high *Ka*/*Ks* values (>1) in *A. ipaensis*.
