# Corrigendum: Genome Sequencing and Analysis of the Peanut B-Genome Progenitor (*Arachis ipaensis*)

**DOI:** 10.3389/fpls.2018.01099

**Published:** 2018-07-31

**Authors:** Qing Lu, Haifen Li, Yanbin Hong, Guoqiang Zhang, Shijie Wen, Xingyu Li, Guiyuan Zhou, Shaoxiong Li, Hao Liu, Haiyan Liu, Zhongjian Liu, Rajeev K. Varshney, Xiaoping Chen, Xuanqiang Liang

**Affiliations:** ^1^South China Peanut Sub-Center of National Center of Oilseed Crops Improvement, Guangdong Provincial Key Laboratory of Crop Genetic Improvement, Crops Research Institute, Guangdong Academy of Agricultural Sciences, Guangzhou, China; ^2^Shenzhen Key Laboratory for Orchid Conservation and Utilization, National Orchid Conservation Center of China and Orchid Conservation and Research Center of Shenzhen, Shenzhen, China; ^3^International Crops Research Institute for the Semi-Arid Tropics, Hyderabad, India; ^4^School of Plant Biology, The Institute of Agriculture, University of Western Australia, University of Western Australia, Crawley, WA, Australia

**Keywords:** *Arachis ipaensis*, genome sequence, genome evolution, polyploidizations, whole genome duplication

In the original article, there was an error. The link of sequencing data was not attached in Supplementary Material section.

A correction has been made to the Supplementary Material section:

The genome assembly and annotation data were deposited in the DRYAD digital repository (https://datadryad.org/). All the data can be downloaded from DRYAD by searching for doi: 10.5061/dryad.hm5vs13 or by directly accessing the link: https://doi.org/10.5061/dryad.hm5vs13.

The authors apologize for this error and state that this does not change the scientific conclusions of the article in any way.

The original article has been updated.

