# Supplementary material for: Genome Sequencing and Analysis of the Peanut B-Genome Progenitor (Arachis ipaensis)
Source: Front Plant Sci. 2018 May 3;9:604. doi: 10.3389/fpls.2018.00604 (PMC5943715; doi:10.3389/fpls.2018.00604)
Supplement: Supplementary File 1 — Supplementary Tables S1–S21 and Figures S1–S53. [file Data_Sheet_1.PDF]

Supplementary Tables:

Table S1 Construction of libraries, generation and filtering of sequencing data used for *A. ipaensis* genome assembly

Library (bp)	Read Count	Read length (bp)	Raw data (Gb)	Sequence depth (x)	Q20 (%)	Q30 (%)	Clean data (Gb)	Effective sequencing depth (x)
250	271,385,710	138	75.07	50.66	94.71	89.20	72.57	42.92
450	158,728,098	150	47.62	32.13	92.66	85.98	46.43	26.33
500	120,588,377	125	30.15	20.34	91.54	83.79	30.15	18.04
800	122,861,513	125	30.72	20.73	89.35	76.64	30.72	18.15
2,000	65,436,373	125	16.36	11.39	96.50	93.39	16.36	10.23
5,000	67,341,830	125	16.84	11.36	95.66	91.87	16.83	10.52
10,000	68,055,845	125	17.01	11.48	83.24	72.15	17.00	10.63
20,000	81,380,937	125	20.35	13.73	81.75	96.55	20.34	12.71
Total			254.11	171.82			250.40	149.53

Table S2 Summary of the *A. ipaensis* genome assembly

	Contig		Scaffold	
	Length(bp)	Number	Length(bp)	Number
N90	736	230,117	39,638	8,567
N80	2,584	136,567	73,651	6,055
N70	4,350	95,534	105,499	4,487
N60	6,143	68,708	136,674	3,330
N50	8,067	48,937	170,050	2,417
N40	10,323	33,661	213,452	1,685
N30	13,010	21,628	262,466	1,095
N20	16,643	12,119	325,363	616
N10	22,462	4,834	438,816	244
Seq_max_length	81,804		1,172,168	
Total_size	1,392,302,041		1,391,700,926	
Total_seq_num	1,008,989		79,408	
>100 bp	1,008,395		79,408	
>2 Kb	156,603		15,778	

Table S3 Distribution of contig and scaffold length of *A. ipaensis* genome

Length (Kb)	Contig				Scaffold			
	Number	Average length (bp)	Total length (Mb)	Percentage (%)	Number	Average length (bp)	Total length (Mb)	Percentage (%)
≥100	0	0	0	0	4,727	211,321	998.91	67.40
≥50	71	56,494	4.01	0.29	7,654	158,327	1,211.83	81.78
≥30	1,476	36,462	53.82	3.87	9,591	134,306	1,288.13	86.92
≥20	7,129	26,337	187.76	13.49	11,166	118,707	1,325.48	89.44
≥10	35,531	16,209	575.92	41.36	12,722	106,025	1,348.85	91.02
≥2	156,603	7,404	1,159.48	83.28	15,778	86,412	1,363.41	92.00
≥1	208,289	5,926	1,234.23	88.65	20,680	66,648	1,370.01	92.44

Table S4 Evaluation of completeness of the genome assembly using core eukaryotic gene mapping approach (CEGMA)

Parameter		Number	Percentage (%)
Total KOGs		458	
One KOG align one gene		106	23.14
One KOG align one gene	overlap>0.8	78	17.03
	overlap >0.5	93	20.31
One KOG align several genes		348	75.98
One KOG align no gene		4	0.87

KOG: Eukaryotic orthologous gene sequences

Table S5 Coverage of transcripts in the *A. ipaensis De Novo* assembly

Dataset	Number	Total length (bp)	Covered by assembly	With >90% sequence in one scaffold		With >50% sequence in one scaffold	
				Number	Percentage(%)	Number	Percentage(%)
>0bp	32,787	14,420,631	95.91	29,797	90.88	31,198	95.15
>200bp	30,692	14,098,162	97.04	28,487	92.82	29,561	96.32
>500bp	12,810	7,368,591	98.88	12,410	96.88	12,601	98.37

Table S6 Estimation of *A. ipaensis* genome size using *K*-mer statistics

K-mer value	*K*-mer number	Depth	Genome size (bp)	Used bases	Used reads	Depth (x)
17	39,847,371,414	27	1,475,828,570	46,793,833,250	374,350,666	30.96

Table S 7 Gene sets used in this study

Species	Code	Database	Version
A. duranensis	ARD	NCBI	Aradu1.0
M. truncatula	MET	Phytozomev	Mt3.5v4 on assembly MedtrA17_3.5 from the Medicago Genome Sequence Consortium
C. arietinum	CIA	NCBI	v1.0
L. japonicus	LOJ	Kazusa.or.jp	lotus_r3.0
C. cajan	CAC	NCBI	C.cajan_V1.0
G. max	GLM	Ensemble	JGI Glyma1.1 annotation of the chromosome-based Glyma1 assembly
G. soja	GLS	NCBI	W05v1.0
P. vulgaris	PHV	NCBI	PhaVulg1_0
V. radiata	VIR	NCBI	Vradiata_ver6
V. angularis	VIA	NCBI	Vigan1.1
M. Borkh	MAB	NCBI	MalDomGD1.0
R. Communis	RIC	NCBI	JCVI_RCG_1.1
A. thaliana	ART	Ensemble	TAIR release 10 acquired from TAIR
O. sativa	ORS	Ensemble	IRGSP-1.0
S. italica	SEI	Ensemble	JGIv2.0
Z. mays	ZEM	Ensemble	AGPv4
S. bicolor	SOB	Ensemble	Sorbi1

Table S8 The statistics of aligned genes between *A. ipaensis* and other plant species with an E value of 1e-10 as cut-off

Species	*A. ipaensis*		Aligned species	
A. ipaensis **vs.**	Matched genes	Percentage (%)	Matched genes	Percentage (%)
A. duranensis	34,981	88.10	30,832	93.08
M. truncatula	23,507	59.21	23,612	46.81
C. arietinum	24,640	62.06	19,583	78.90
L. japonicus	23,263	58.59	23,732	60.29
C. cajan	26,760	67.40	26,329	54.48
G. max	24,513	61.74	39,974	73.79
G. soja	24,064	60.61	36,301	72.03
P. vulgaris	23,280	58.63	21,010	74.68
V. radiata	25,122	63.27	20,256	76.05
V. angularis	25,178	63.41	20,509	76.68
M. Borkh	21,178	53.34	28,988	60.55
R. Communis	20,065	50.54	13,078	63.67
A. thaliana	16,127	40.62	13,170	47.67
O. sativa	13,834	34.84	10,950	30.69
S. italica	13,313	33.53	10,935	30.83
Z. mays	15,047	37.90	17,233	43.63
S. bicolor	13,700	34.51	11,174	32.39

Table S9 General statistics of predicted protein-coding genes in *A. ipaensis* and comparison with other plant species

Gene set	Species	Number of genes	Average gene length (bp)	Average CDS length (bp)	Average exon per gene	Average intron length (bp)
Reference	*A. ipaensis*	39,704	3,741.00	249.60	3.90	625.00
Homology*	*A. duranensis*	33,124	3,622.35	259.25	5.07	494.37
	M. truncatula	50,444	2,621.04	243.28	4.04	439.11
	C. arietinum	24,819	4,371.35	236.82	5.84	541.11
	L. japonicus	39,366	3,031.76	246.78	4.1	508.97
	C. cajan	48,331	2,361.96	266.96	3.61	536.92
	G. max	54,174	3,943.88	228.7	5.45	499.64
	G. soja	50,399	3,475.09	222.72	4.94	507.06
	P. vulgaris	28,134	3,610.95	240.26	5.35	463.72
	V. radiata	26,636	4,233.83	249.28	5.66	544.97
	V. angularis	26,746	4,350.91	246.52	5.65	565.26
	M. Borkh	47,875	3,825.65	247.67	5.12	544.28
	R. Communis	20,540	3,887.04	238.11	5.83	439.83
	A. thaliana	27,628	2,372.75	237.35	5.27	165.57
	O. sativa	35,679	3,090.07	262.97	4.29	440.53
	S. italica	35,471	2,545.79	254.57	4.51	333.14
	Z. mays	39,498	4,150.93	241.64	5.07	590.78
	S. bicolor	34,496	2,857.51	269.92	4.38	450.25

*Based on the 17 sequenced plant species, the protein sequences were used to perform gene prediction, taking one species each time. These sequences were mapped to the genome assembly using TblastN with an E-value of 1e-10 as cutoff. Then homologous genome sequences were aligned against the matching proteins for accurate spliced alignments.

Table S10 Functional annotation of predicted genes in *A. ipaensis*

	Database	Number	Percentage (%)
Annotated	InterPro	28,304	71.29
	GO	17,458	43.97
	KEGG	28,636	72.12
	Swissprot	27,460	69.16
	TrEMBL	39,631	99.82
Un-annotated		59	0.15

Table S11 Summary of predicted non-protein coding genes in *A. ipaensis* genome

Type	Sub-type	Number	Average length (bp)	Total length (bp)	Percentage (%)
Pre-miRNA		71	123	8,749	0.000590
Pre-tRNA		2,914	75	219,871	0.014836
Pre-rRNA	5S rRNA	108	106	11,451	0.000773
	5.8S rRNA	55	133	7,334	0.000495
	18S rRNA	82	364	29,823	0.002012
	28S rRNA	68	141	9,599	0.000648
	Total rRNA	313	186	58,207	0.003928
Pre-snRNA	CD- box snRNA	111	102	11,333	0.000765
	Splicing snRNA	4	122	487	0.000033
	HACA-box	36	132	4,734	0.000319
	Total snRNA	152	111	16,886	0.001139

Table S12 Summary of simple sequence repeats and primer designed in *A. ipaensis*

SSR statistics	Number	Percentage (%)
Total number of identified SSRs (Excluding compound repeats)	188,075	
Total numbers of primer pairs designed	80,218	42.65
Number of di-nucleotide repeats	90,993	48.38
Number of tri-nucleotide repeats	52,771	28.06
Number of tetra-nucleotide repeats	28,314	15.05
Number of penta-nucleotide repeats	12,102	6.43
Number of hexa-nucleotide repeats	3,895	2.07
Number of compound repeats	26,531	14.11

Table S13 Identification of variations among the A genomes (two re-sequenced genotypes) and B genomes (three re-sequenced genotypes)

Sample ID	Ploidy (genome)	Species	Re-sequencing data	Number of SNP	Number of InDel	Total variations
ICG_8123	Diploid (A)	*A. duranensis*	50,951,850,164	4,133,748	252,538	4,386,286
ICG_8138	Diploid (A)	*A. duranensis*	50,887,480,036	4,169,753	254,733	4,424,486
ICG_8960	Diploid (B)	*A. magna*	46,660,603,170	5,874,227	291,244	6,165,471
ICG_8209	Diploid (B)	*A. batizocoi*	46,261,801,842	5,266,598	283,597	5,550,195
ICG_13160	Diploid (B)	*A. batizocoi*	49,197,182,820	5,243,951	279,761	5,523,712
			Total	24,688,277	1,361,873	26,050,150

Table S14 Details on gene family for *A. ipaensis* and other plant species

Species	Total predicted genes	Genes in orthologous groups	Genes not in orthologous groups [1]	Total orthologous groups[2]	Species-specific homolog groups[3]	Average genes group
A. ipaensis	39,704	34,632	5,072	16,791	959 (6,443)	2.06
A. duranensis	33,124	29,125	3,999	17,220	872 (2,047)	1.69
M. truncatula	50,444	37,890	12,554	18,410	1,930 (8,158)	2.06
C. arietinum	24,819	24,545	274	15,609	344 (943)	1.57
L. japonicus	39,366	29,818	9,548	17,647	2,574 (6,050)	1.69
C. cajan	48,331	40,646	7,685	18,335	1,661 (7,880)	2.22
G. max	54,174	47,003	7,171	23,365	713 (970)	2.01
G. soja	50,399	42,865	7,534	21,994	208 (755)	1.95
P. vulgaris	28,134	26,322	1,812	17,242	277 (519)	1.53
V. radiata	26,636	25,135	1,501	16,591	259 (528)	1.51
V. angularis	26,746	25,167	1,579	16,740	243 (437)	1.5
M. Borkh	47,875	42,885	4,990	16,055	2,009 (5,223)	2.67
R. Communis	20,540	18,848	1,692	14,281	388 (704)	1.32
A. thaliana	27,628	24,735	2,893	14,599	1,827 (4,079)	1.69
O. sativa	35,679	25,758	9,921	17,620	1,394 (3,273)	1.46
S. italica	35,471	27,494	7,977	18,163	615 (1,363)	1.51
Z. mays	39,498	33,755	5,743	22,996	5,330 (8,057)	1.47
S. bicolor	34,496	29,185	5,311	18,364	522 (2,030)	1.59

[1]Predicted genes that were not organized into groups using OrthoMCL. [2]Orthologous groups containing at least one gene from the indicated species. [3]Groups containing putative paralogs from the indicated species, but lacking genes from other species.

Table S15 Details on single copy orthologs and unique paralogs in *A. ipaensis* and other plant species

Species	Single-copy orthologs	Co-orthologs[1]	Unique paralogs	Other orthologs[2]	Unclustered genes
A. ipaensis	1,624	16,634	6,443	9,931	5,072
A. duranensis	1,658	14,907	2,047	10,513	3,999
M. truncatula	1,308	18,220	8,158	10,204	12,554
C. arietinum	828	12,413	943	10,361	274
L. japonicus	1,106	13,575	6,050	9,087	9,548
C. cajan	1,367	20,431	7,880	10,968	7,685
G. max	6,172	35,389	970	4,472	7,171
G. soja	6,008	29,830	755	6,272	7,534
P. vulgaris	1,526	13,205	519	11,072	1,812
V. radiata	1,246	12,269	528	11,092	1,501
V. angularis	1,308	12,319	437	11,103	1,579
M. Borkh	594	33,005	5,223	4,063	4,990
R. Communis	460	6,392	704	11,292	1,692
A. thaliana	355	11,964	4,079	8,337	2,893
O. sativa	2,810	9,813	3,273	9,862	9,921
S. italica	3,575	12,703	1,363	9,853	7,977
Z. mays	3,515	17,380	8,057	4,803	5,743
S. bicolor	3,689	13,177	2,030	10,289	5,311

[1]Co-orthologous genes, also known as “in-paralogs”, are derived from duplication in the indicated genome. [2]Other orthologs represent gene duplication events internal to the overall set, but basal more than two of the compared species.

Table S16 Summary of the syntenic blocks between *A. ipaensis* and other genomes

A. ipaensis **vs.**	Syntenic blocks	Average syntenic gene pairs / block	Syntenic gene pairs	Mean block length (bp)
A. ipaensis	46	7.9	363	195,541
A. duranensis	1,175	10.9	12,753	284511/205781
M. truncatula	871	10.4	9,055	230736/223466
C. arietinum	872	10.1	8,778	215330/223961
L. japonicus	644	9.9	6,395	174275/211984
C. cajan	845	9.4	7,917	195650/188250
G. max	1,910	10.3	19,699	235765/224689
G. soja	1,628	9.7	15,756	169979/211159
P. vulgaris	979	10.3	10,097	292270/223043
V. radiata	925	10.2	9,409	236091/219208
V. angularis	934	9.9	9,271	238467/213351
M. Borkh	667	8.2	5,447	382673/235617
R. Communis	576	9.2	5,311	163132/179241
A. thaliana	369	8.2	3,031	65496/268048
O. sativa	73	7.7	559	181971/265335
S. italica	118	7.2	850	290195/283029
Z. mays	42	7.6	319	853618/277730
S. bicolor	138	7.3	1,002	336686/286015

Table S17 Comparison of putative disease resistance gene families in *A. ipaensis* with other sequenced plant species

Class	Subfamily	*A. ipaensis*	*A. duranensis*	*C. cajan*	*G. max*	*M. truncatula*	*O. sativa*	*A. thaliana*
CC-NBS	CN	51	39	69	22	15	14	10
CC-NBS-LRR	CNL	100	102	148	100	78	74	50
LRR	L	5	9	6	0	0	0	0
NBS	N	166	175	286	126	70	86	60
NBS-LRR	NL	200	164	420	168	265	487	25
Kin-LRR	RLK	64	69	79	6	3	0	2
Kin-LRR-GNK2	RLK-GNK2	43	93	88	99	54	60	47
ser/thr-LRR	RLP	543	671	516	504	218	409	257
ser/thr-LRR-Malection	RLP-Malectin	2	1	1	0	0	0	0
RPW8-NBS-LRR	RPW8-NL	5	6	8	4	2	1	4
TIR	T	29	33	29	31	64	2	35
TIR-NBS	TN	0	0	0	0	0	0	5
TIR-NBS-LRR	TNL	158	173	467	143	210	0	125
TIR-NBS-LRR-OT	TNL-OT	3	5	4	0	0	0	0
Other	Mlo-like	34	26	27	40	19	14	22
	Pto-like	14	18	14	0	0	0	0
	Unclassified	20	24	17	0	0	1	7
Total		1,437	1,608	2,179	1,243	998	1,148	649

Table S18 Summary of nodulation associated genes in *A. ipaensis* and other plant species

	Nodulation regulatory	Nodulin	Total
A. ipaensis	4	12	16
A. duranensis	8	30	38
M. truncatula	13	43	56
C. arietinum	7	32	39
C. cajan	11	35	46
G. max	16	54	70
G. soja	15	60	75
A. thaliana	0	2	2
O. sativa	0	1	1
S. italica	0	7	7
Z. mays	0	0	0
S. bicolor	0	1	1

Table S19 Summary of drought tolerance related TFs in upland crops and hygrophilous plants

	Transcription factors						
Species	ERF	MYB	MYB_related	NAC	WRKY	bZIP	Total
A. ipaensis	170	185	129	138	100	116	838
A. duranensis	172	199	179	151	109	142	952
M. truncatula	106	101	84	75	81	66	513
G. max	330	369	265	247	233	266	1,710
G. soja	262	359	285	272	212	200	1,590
A. thaliana	139	168	97	138	90	127	759
O. sativa	163	130	106	170	128	140	837
Z. mays	205	203	169	190	163	218	1,148

Table S20 Summary of heat-shock proteins (Hsps)/chaperones in upland crops and hygrophilous plants

Species	Heat-shock proteins (Hsps)/chaperones				Total
	Hsp60	Hsp70	Hsp90	small Hsps (20+40)	
A. ipaensis	0	118	6	23	147
A. duranensis	0	23	5	32	60
C. arietinum	0	15	2	23	40
C. cajan	0	8	5	28	41
G. max	0	8	15	41	64
G. soja	0	34	13	62	109
A. thaliana	3	29	0	3	35
O. sativa	0	1	1	21	23

Table S21 Summary of putative oil biosynthesis genes in *A. duranensis* and other plants

Class	*A. ipaensis*	*A. duranensis*	*G. max*	*A. thaliana*	*O. sativa*	*Z. mays*
Arachidonic acid metabolism	39	52	89	53	35	46
Biosynthesis of unsaturated fatty acids	59	119	155	112	136	191
Cutin, suberine and wax biosynthesis	167	56	64	36	27	36
Ether lipid metabolism	67	118	150	87	81	112
Fatty acid biosynthesis	85	181	285	151	146	227
Fatty acid degradation	122	390	569	240	261	389
Fatty acid elongation	73	57	78	56	51	69
Glycerolipid metabolism	199	311	484	186	204	305
Glycerophospholipid metabolism	211	243	359	186	173	258
Linoleic acid metabolism	232	59	103	29	40	39
Sphingolipid metabolism	137	46	83	48	48	74
Steroid biosynthesis	82	54	92	54	46	63
Steroid hormone biosynthesis	0	14	17	3	6	15
Synthesis and degradation of ketone bodies	16	64	88	32	48	72
α-Linolenic acid metabolism	124	165	255	107	117	154
total	1,613	1,929	2,871	1,380	1,419	2,050

Supplementary Figures:

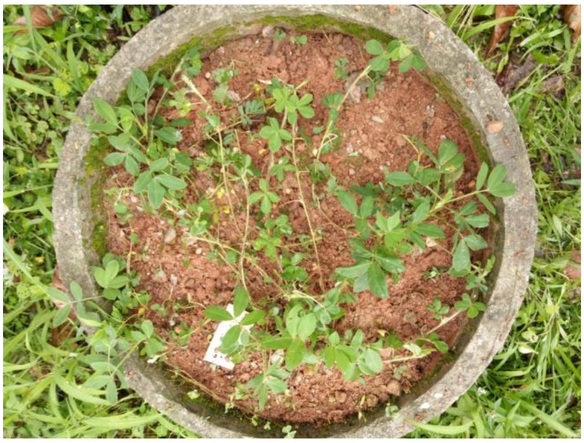

Figure S1 The reference genome accession, *A. ipaensis* (ICG_8206)

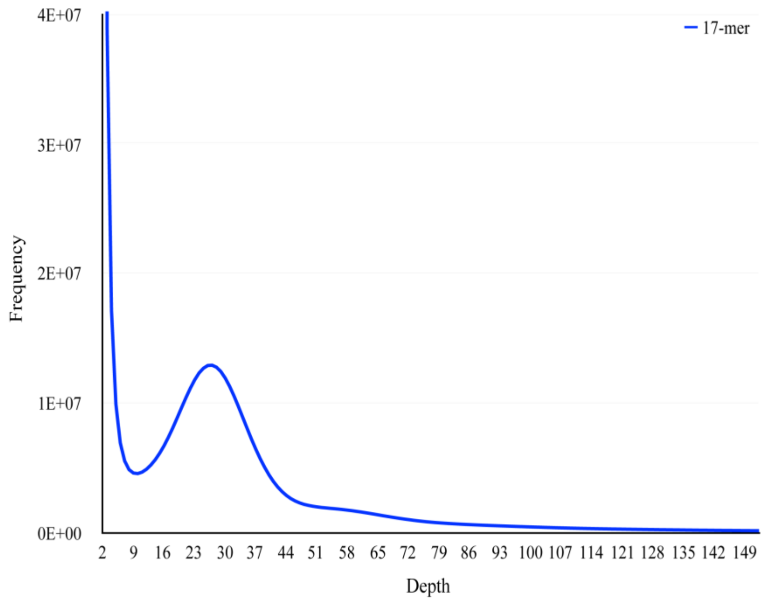

Figure S2 Distribution of sequence depth across the assembled genome. In the *K*-mer distribution, the main peak was estimated to be 27. Total number of 39,847,371,414 *K*-mers was resulted using 374,350,666 reads. Genome size was calculated by following the formula: Genome size = *K*-mer number/Peak depth. Consequently, the genome size of *A. ipaensis* was estimated to be ~1,475.83 Mb.

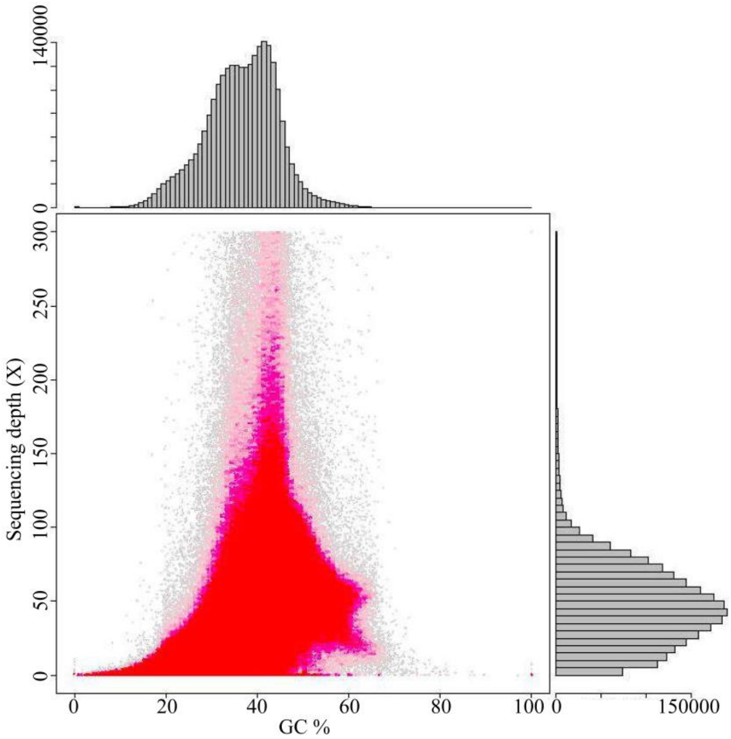

Figure S3 Distribution of GC content and sequence depth. X and Y-axis represent GC content and average sequence depth, respectively. The GC content and average sequence depth were calculated by using 500 bp windows without any sequence overlap.

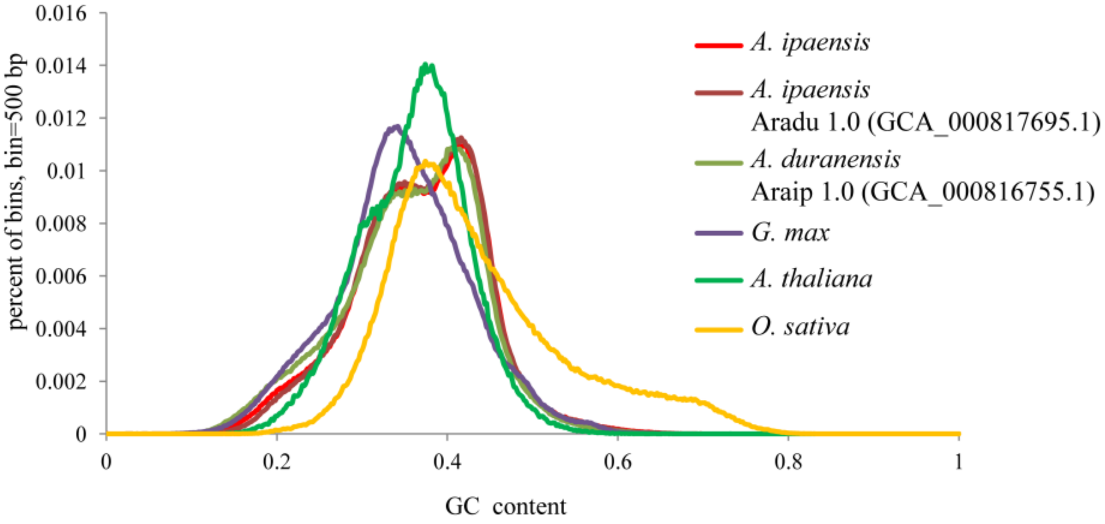

Figure S4 Comparison of GC content distribution among different plant species. The Aradu 1.0 (GCA_000817695.1) and Araip 1.0 (GCA_000816755.1) were used as the two *Archis* reference genomes, respectively.

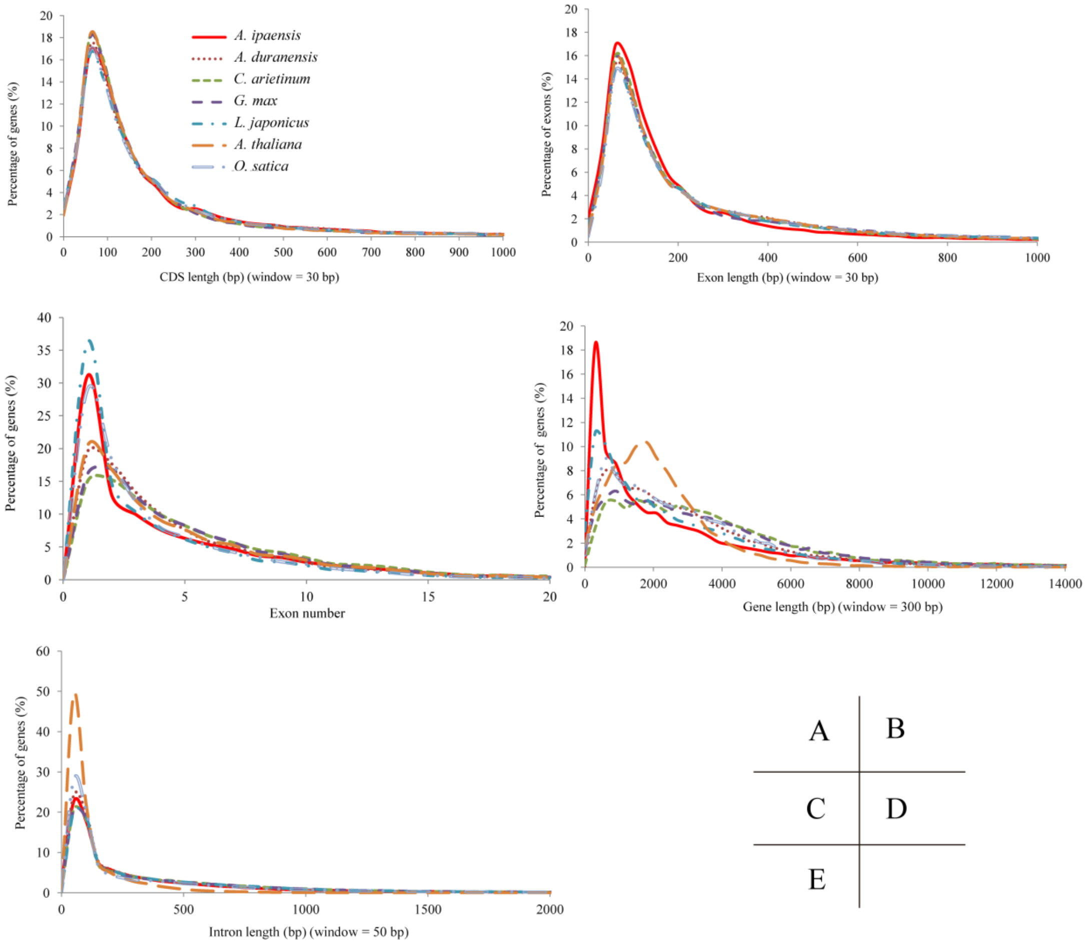

Figure S5 Comparison of CDS length (A), exon length (B), exon number (C), gene length (D) and intron length (E) among *A. ipaensis* and other plant species.

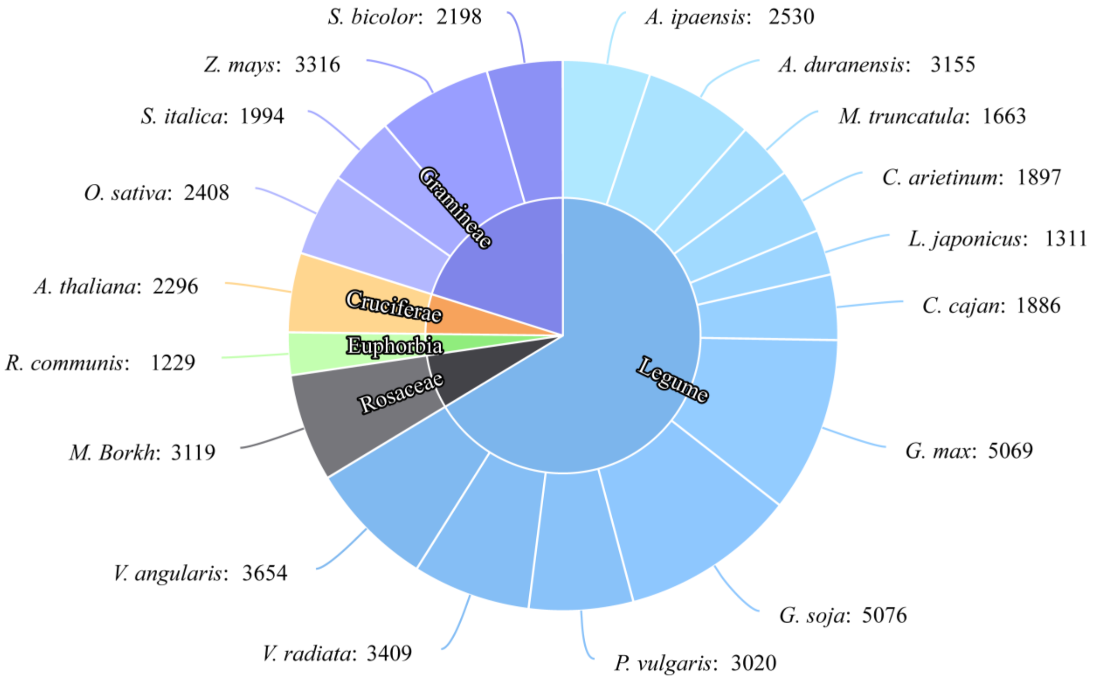

Figure S6 Transcription factors identified in *A. ipaensis* and other plant species

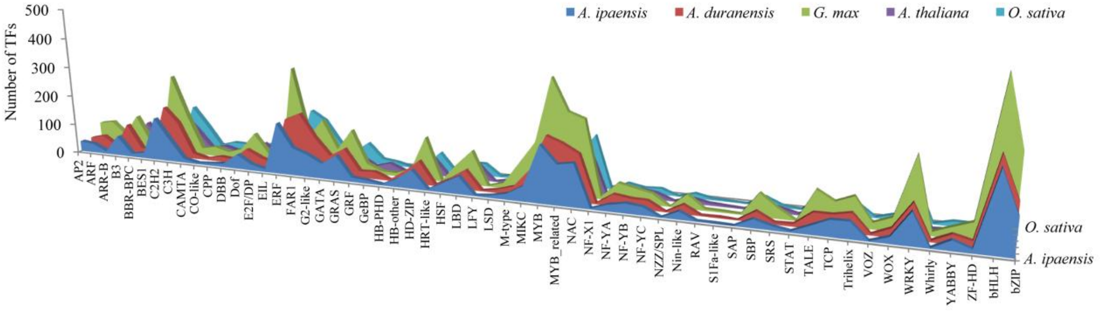

Figure S7 Distribution of TF genes identified in different TF families in five plant species

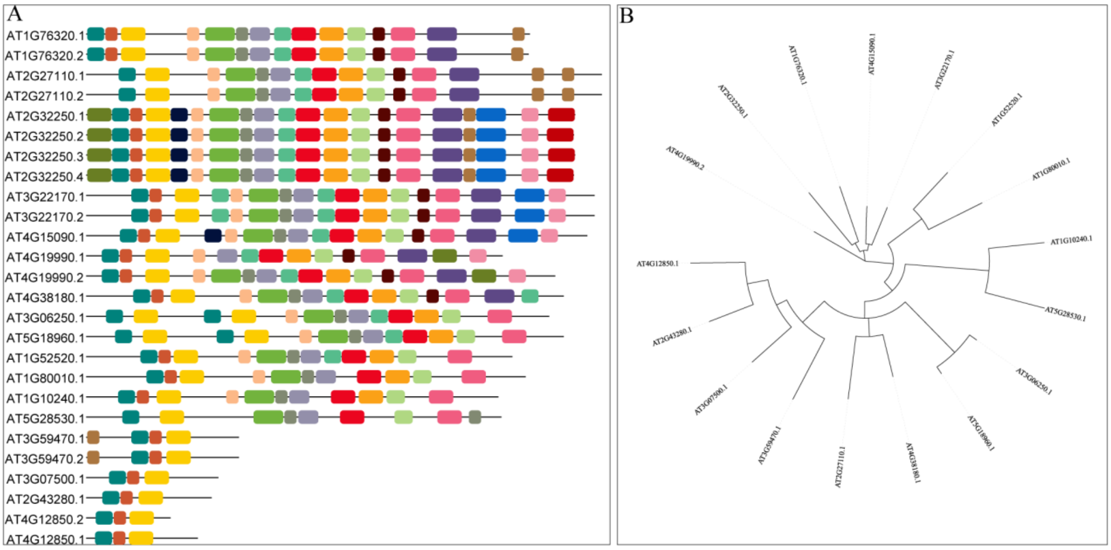

Figure S8 FAR1 transcription factor families in *A. thalianal*. A: Identification of conserved motifs of each FAR1 member. B: Phylogenetic tree of FAR1 members.

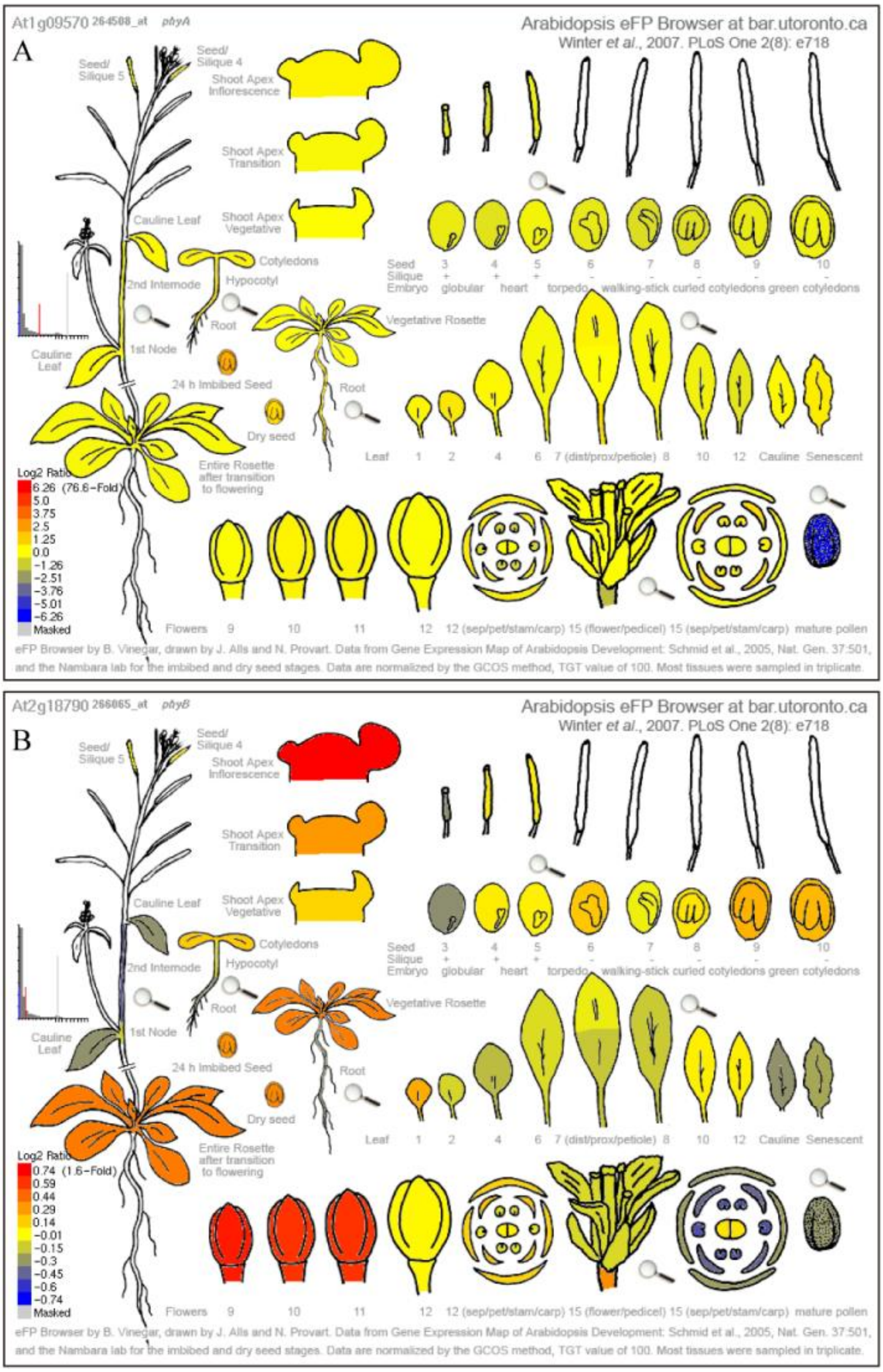

Figure S9 Expression pattern of *phyA* (A) and *phyB* (B) in *A. thalianal*. The expression levels were retrieved from the Arabidopsis eFP Browser (http://bar.utoronto.ca/efp/cgi-bin/efpWeb.cgi). The color scale bar in each image shows the relative expression levels for the individual gene.

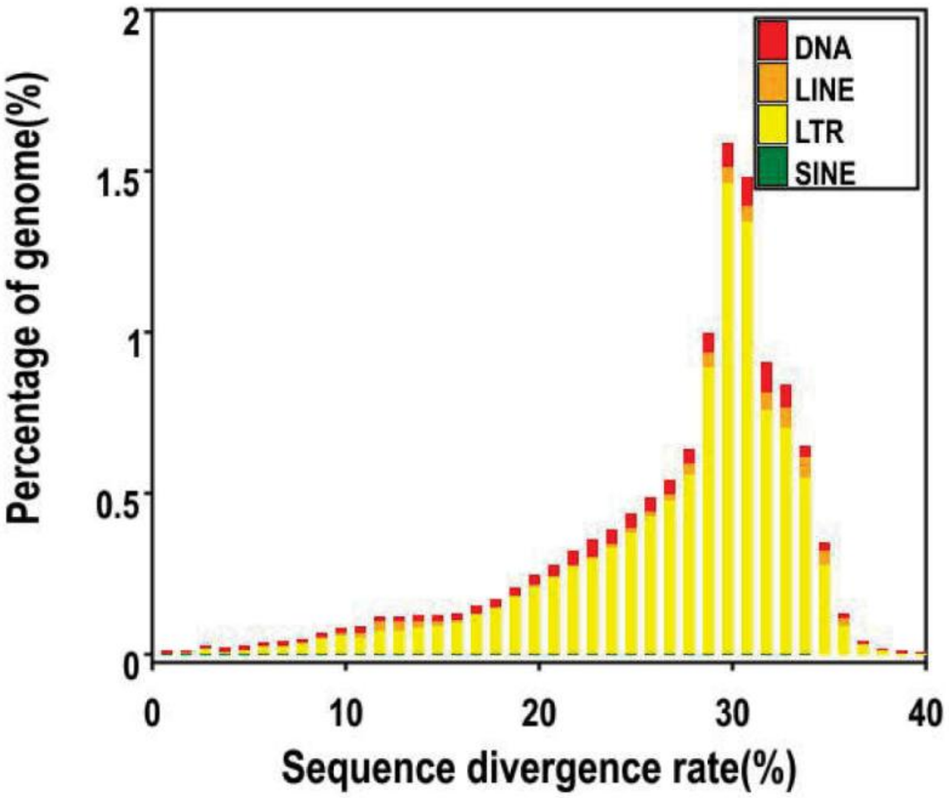

Figure S10 Distribution of divergence rate of different type of transposable elements (TEs) (DNA elements, LINE, LTR, SINE) in the *A. ipaensis* genome. Divergence rate was calculated between the identified TEs in the genome and the consensus sequence in the TE library (Repbase: http://www.girinst.org/repbase). DNA, DNA elements; LINE, long interspersed nuclear elements; LTR, long terminal repeat transposable element; SINE, short interspersed nuclear elements.

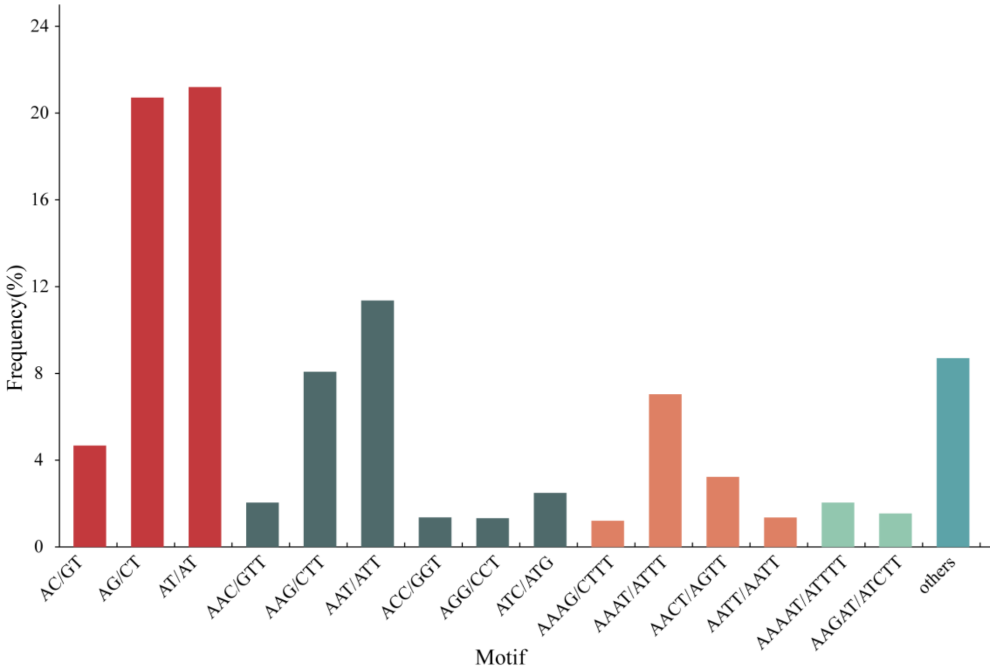

Figure S11 Proportion of different SSR motifs in *A. ipaensis* genome assembly

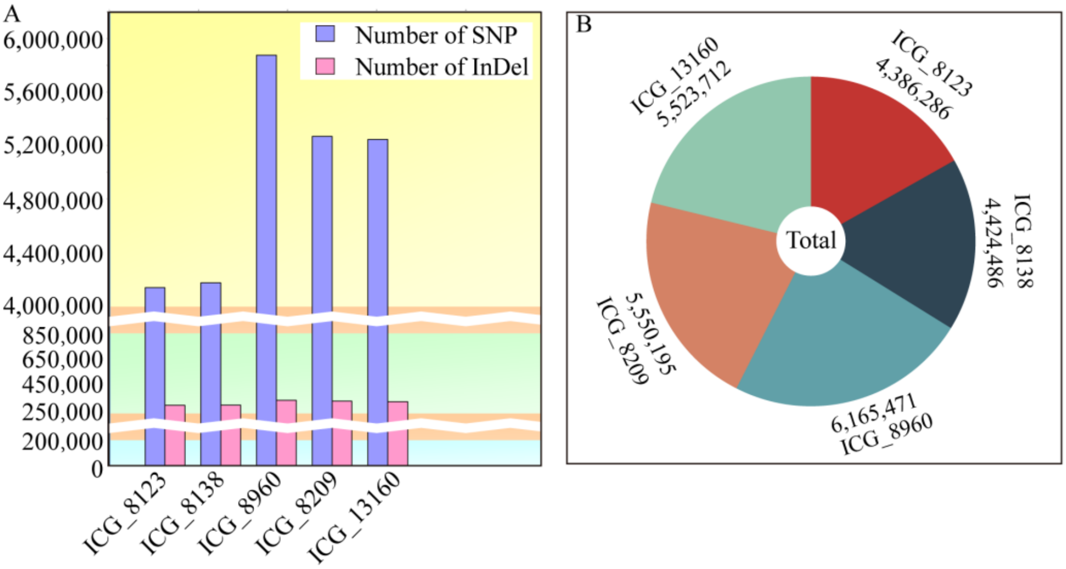

Figure S12 Distribution of SNPs and InDels (A) and total of variations (B) in different re-sequenced genome genotypes

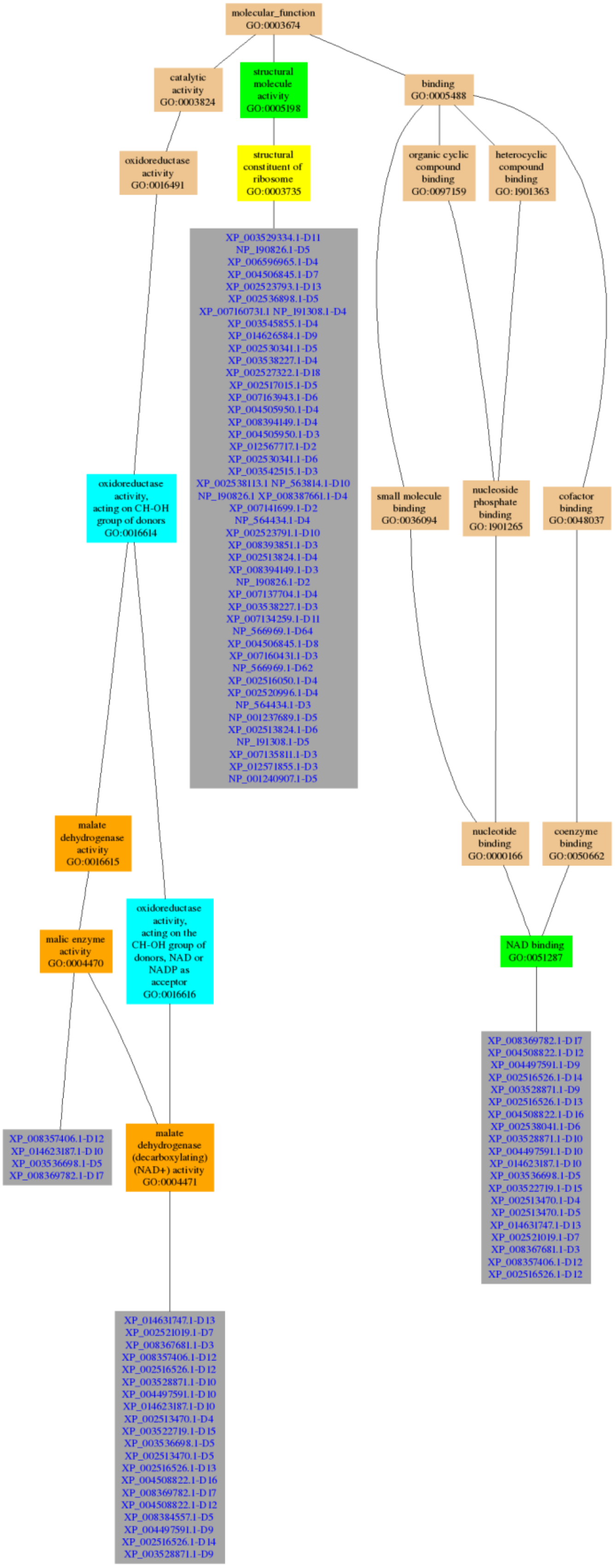

Figure S13 Go enrichment for molecular function

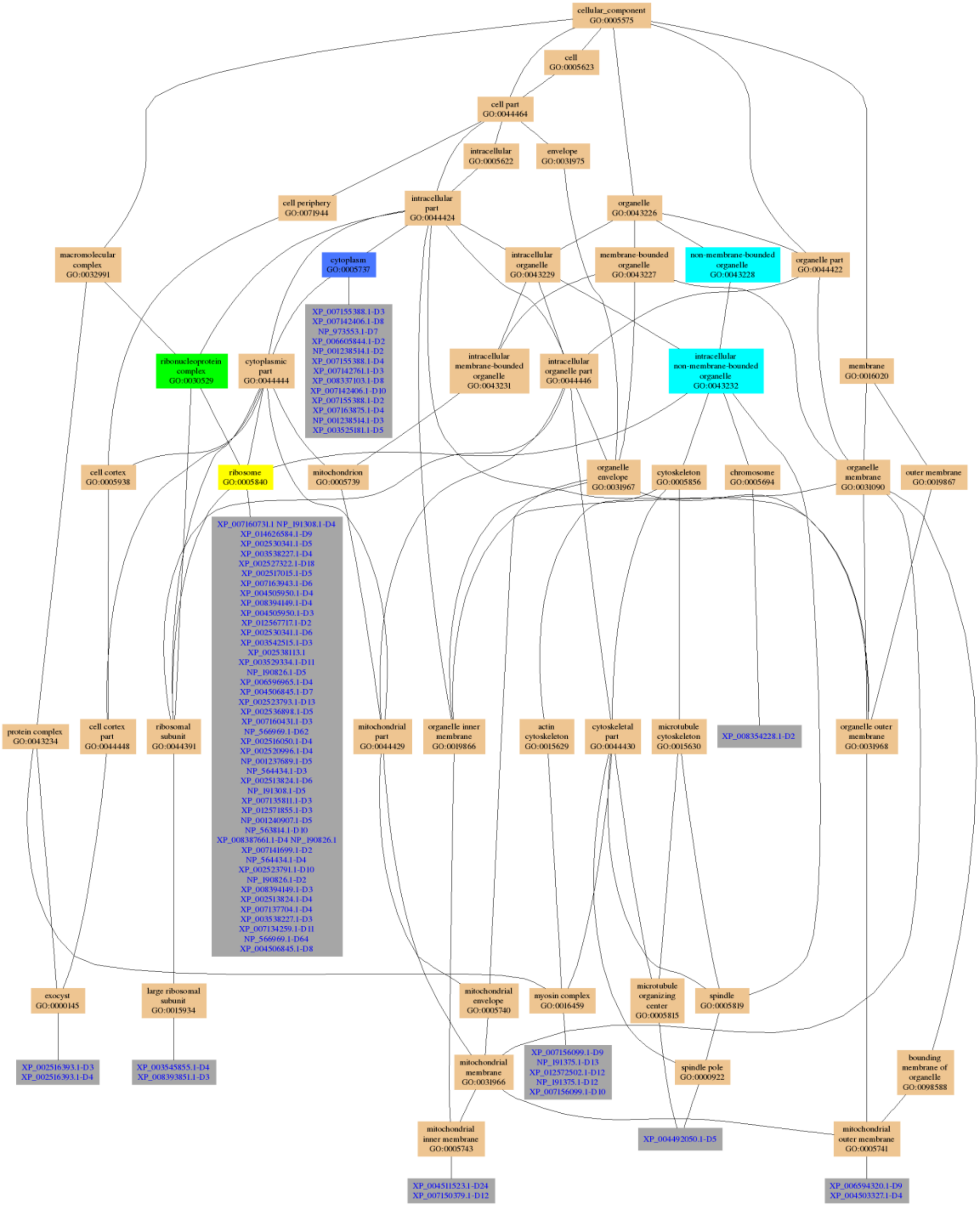

Figure S14 Go enrichment for cellular component

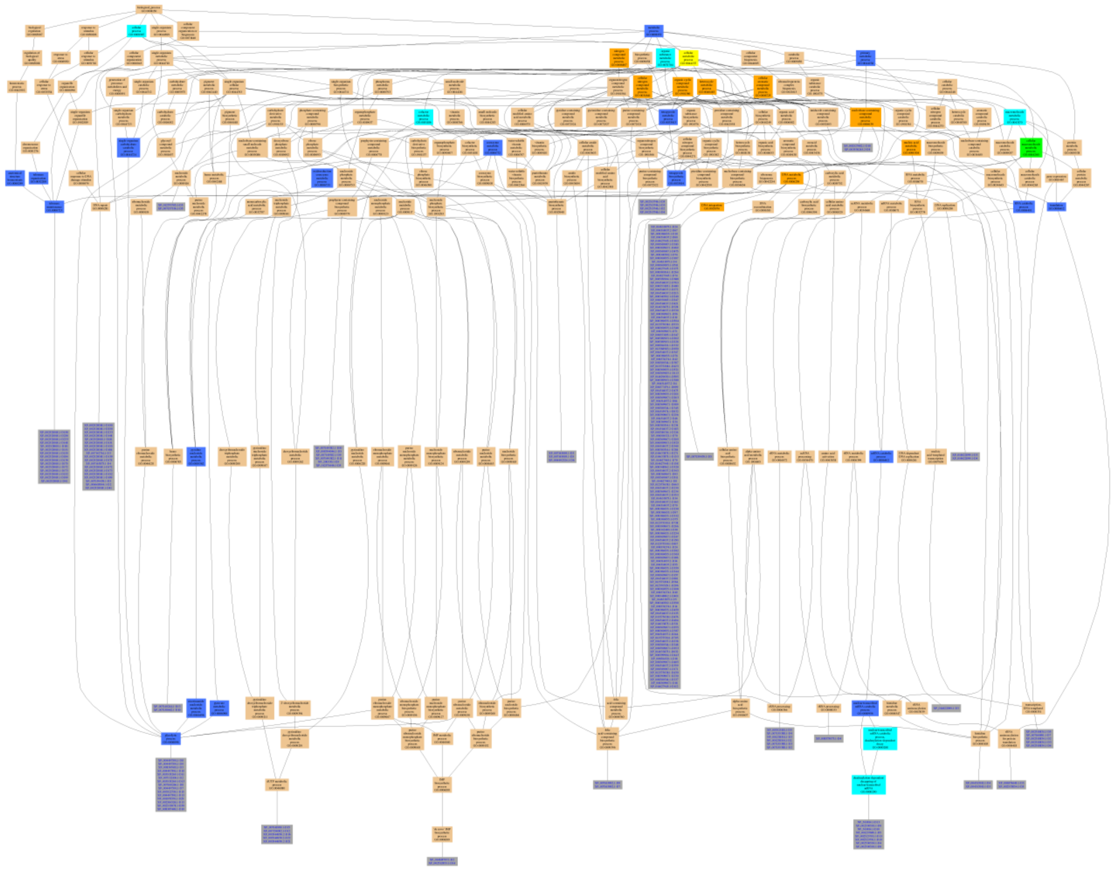

Figure S15 Go enrichment for biological process

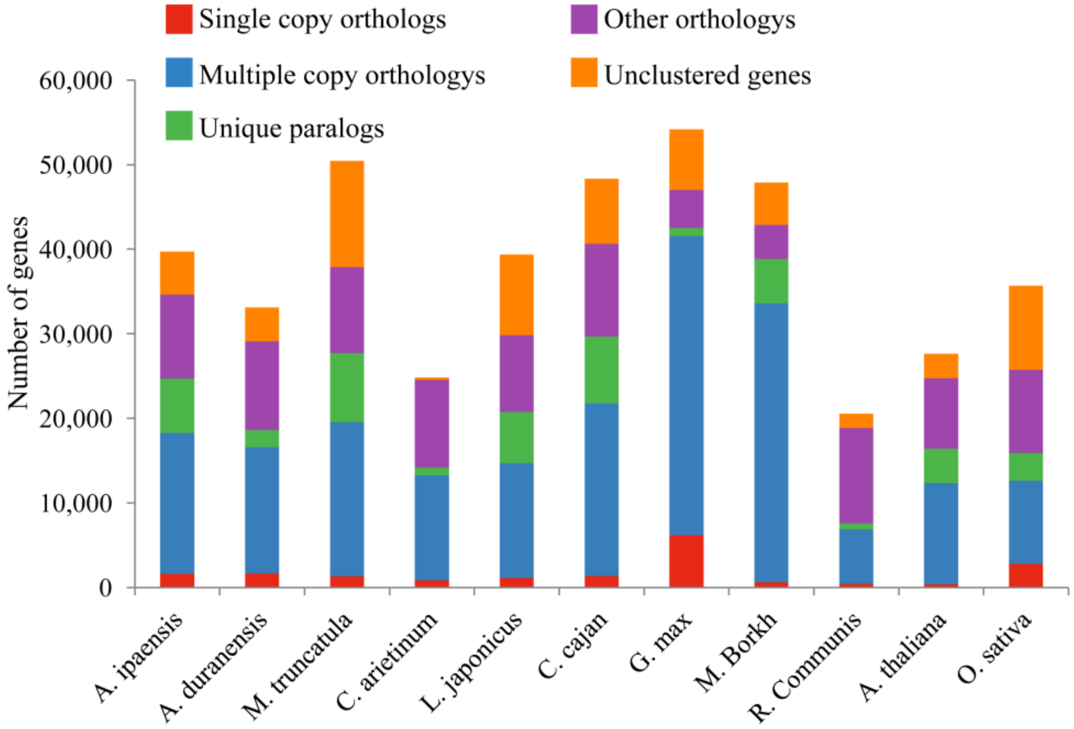

Figure S16 An overview on orthologs and paralogs genes among *A. ipaensis* and other plant species

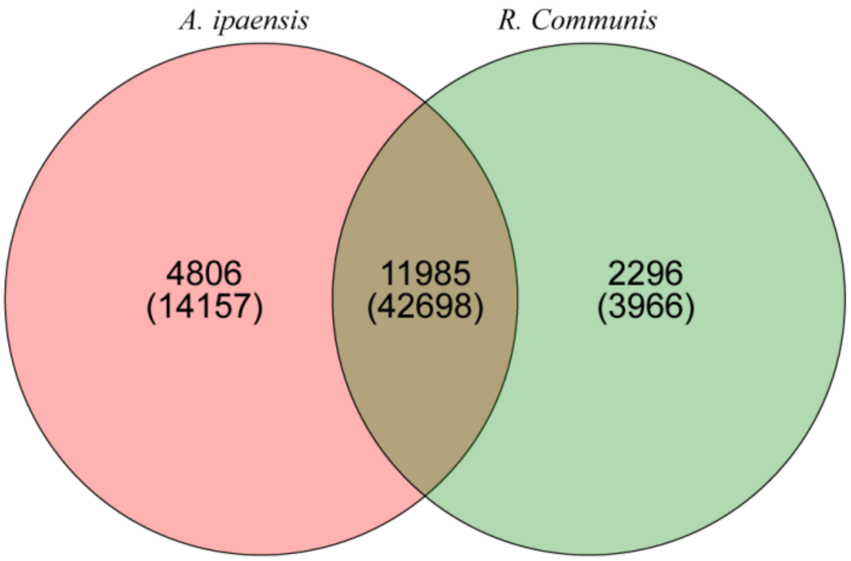

Figure S17 Venn diagram showing shared and unique gene families between *A. ipaensis* and *R. Communis*

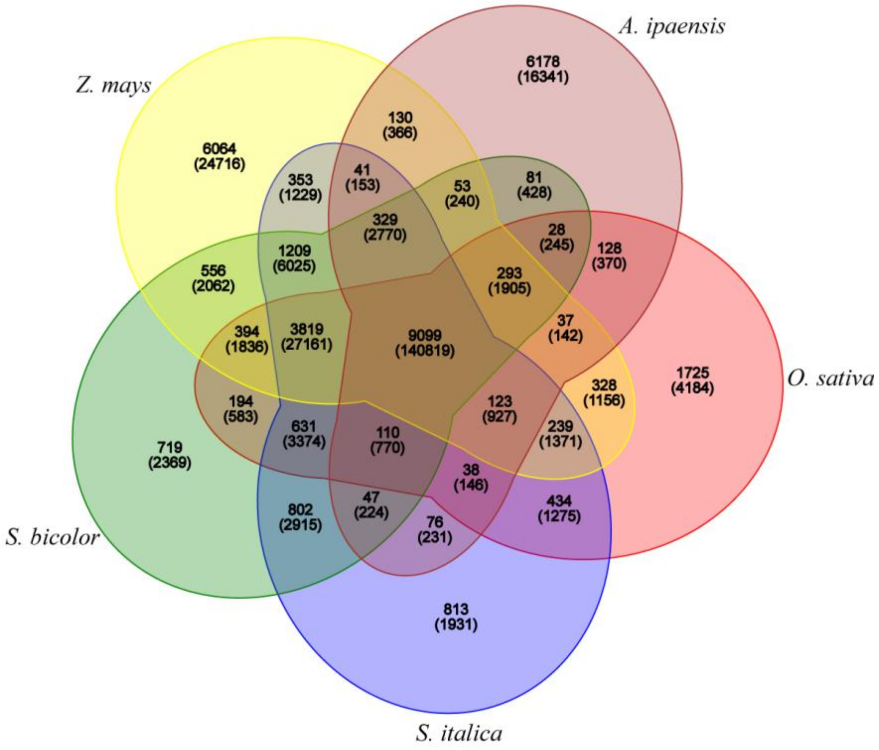

Figure S18 Venn diagram showing shared and unique gene families among *A. ipaensis* and gramineae crops

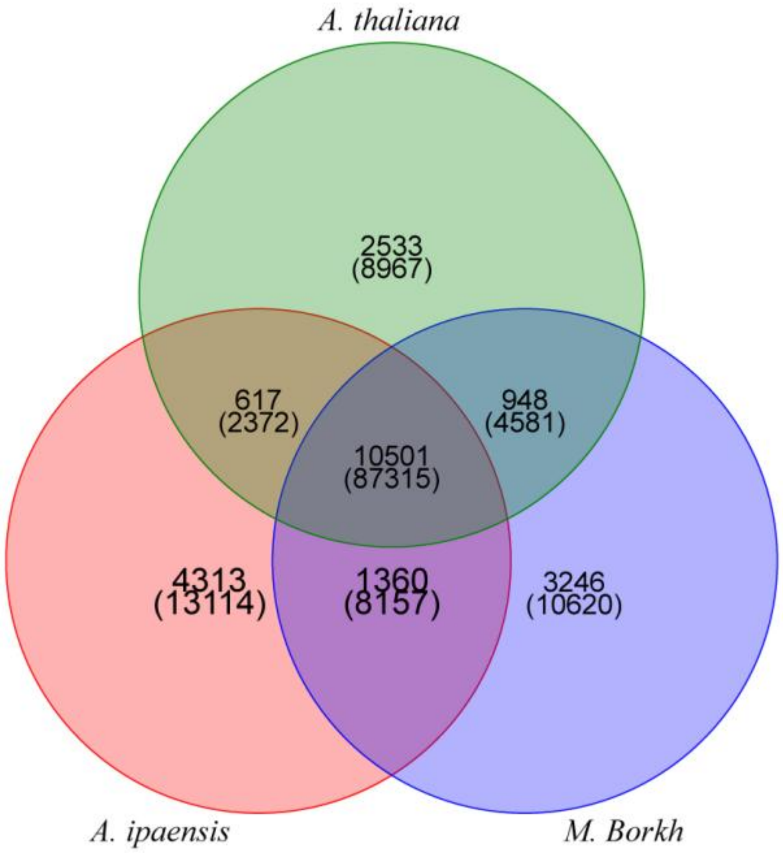

Figure S19 Venn diagram showing shared and unique gene families among distantly related plant species

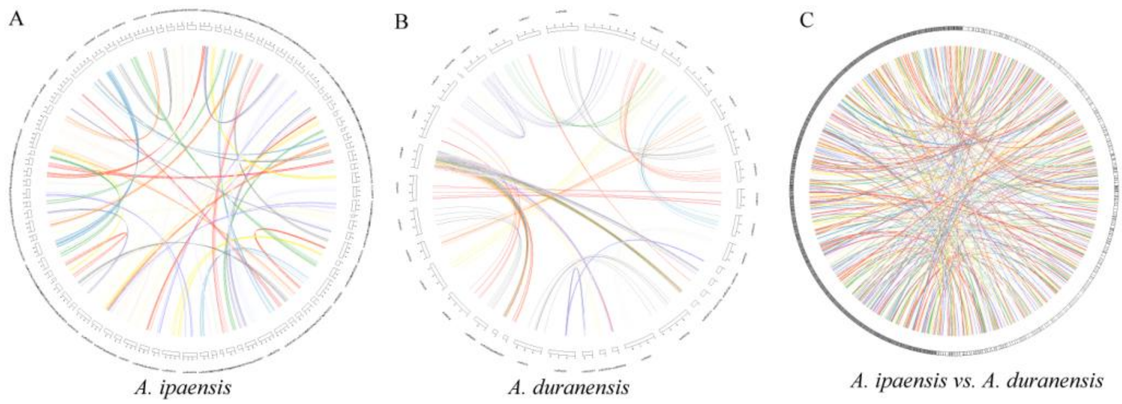

Figure S20 Syntenic blocks of *A. ipaensis*, *A. duranensis* and *A. ipaensis* vs. *A. duranensis* genomes. The Arachis_duranensis_Genome_V2.0 was used as *A. duranensis* reference genome (http://ceg.icrisat.org/dppga/Manuscript.html).

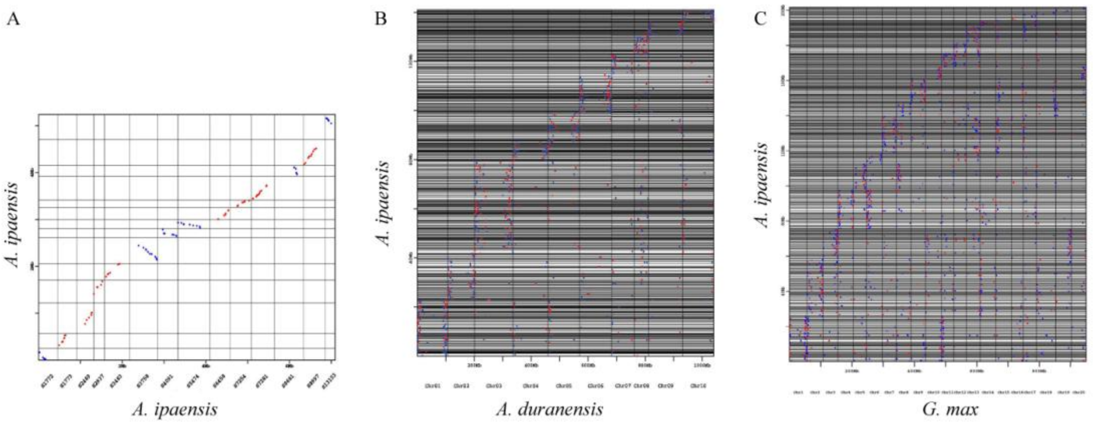

Figure S21 Syntenic blocks of *A. ipaensis* (A), *A. duranensis* (B) and *A. ipaensis* vs. *G. max* (C) genomes. The Arachis_duranensis_Genome_V2.0 was used as *A. duranensis* reference genome (http://ceg.icrisat.org/dppga/Manuscript.html).

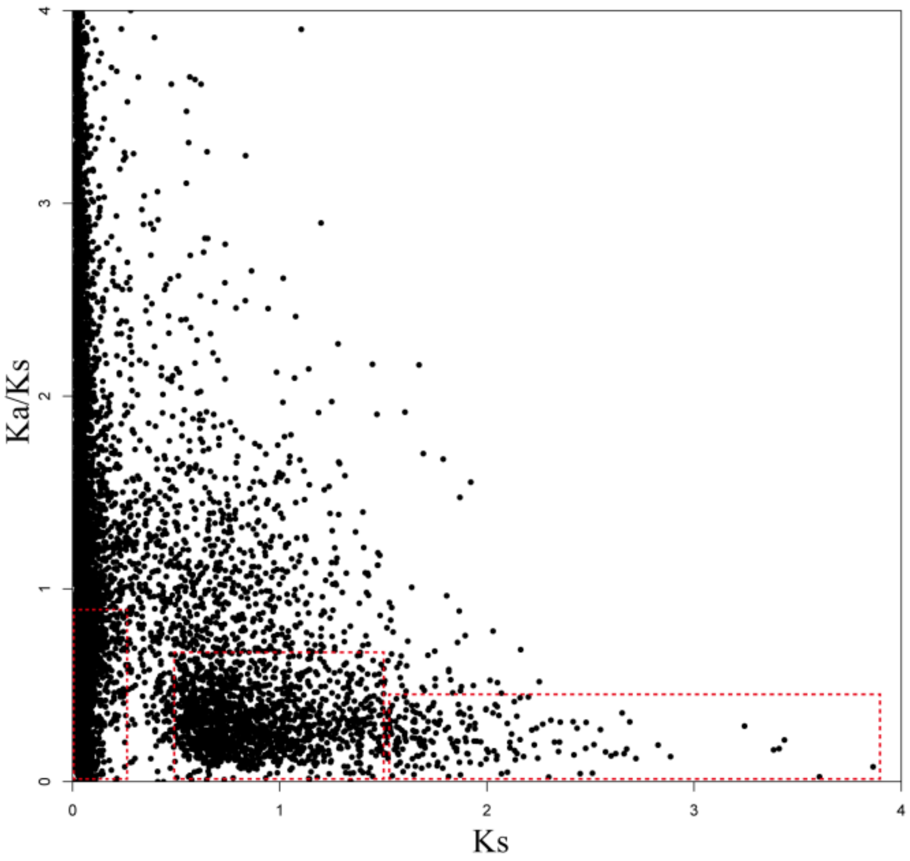

Figure S22 Scatter plot showing distribution of *Ka*/*Ks* (*ω*) with respect to *Ks* between gene pairs present in the collinear blocks of *A. ipaensis* vs *A. duranensis*

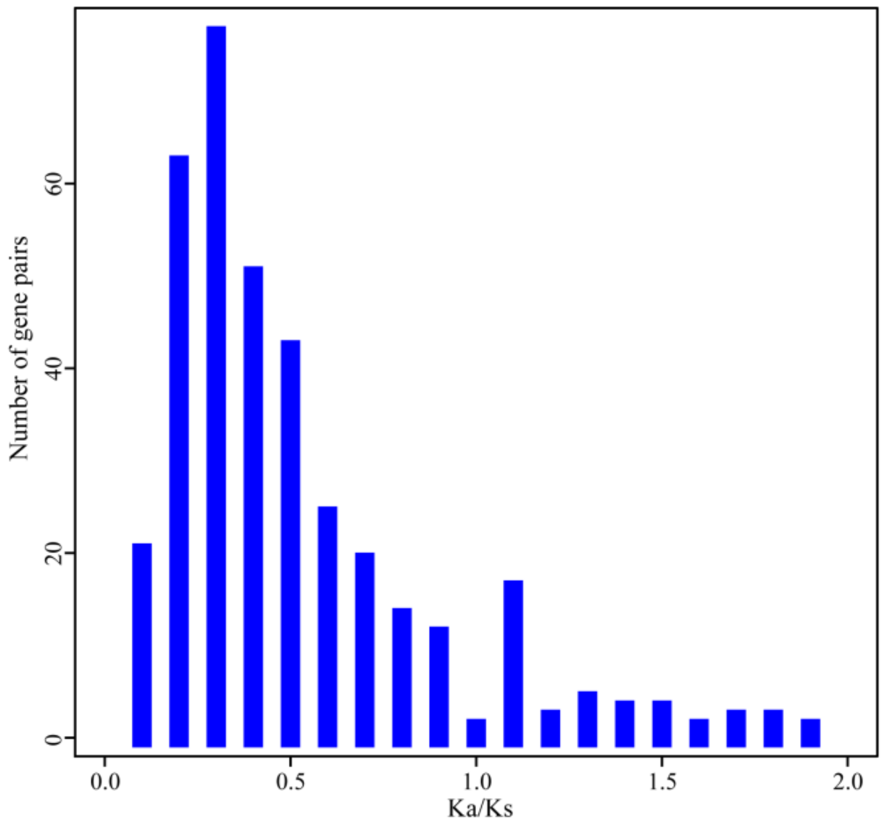

Figure S23 *Ka*/*Ks* distribution analysis of *A. ipaensis* gene pairs

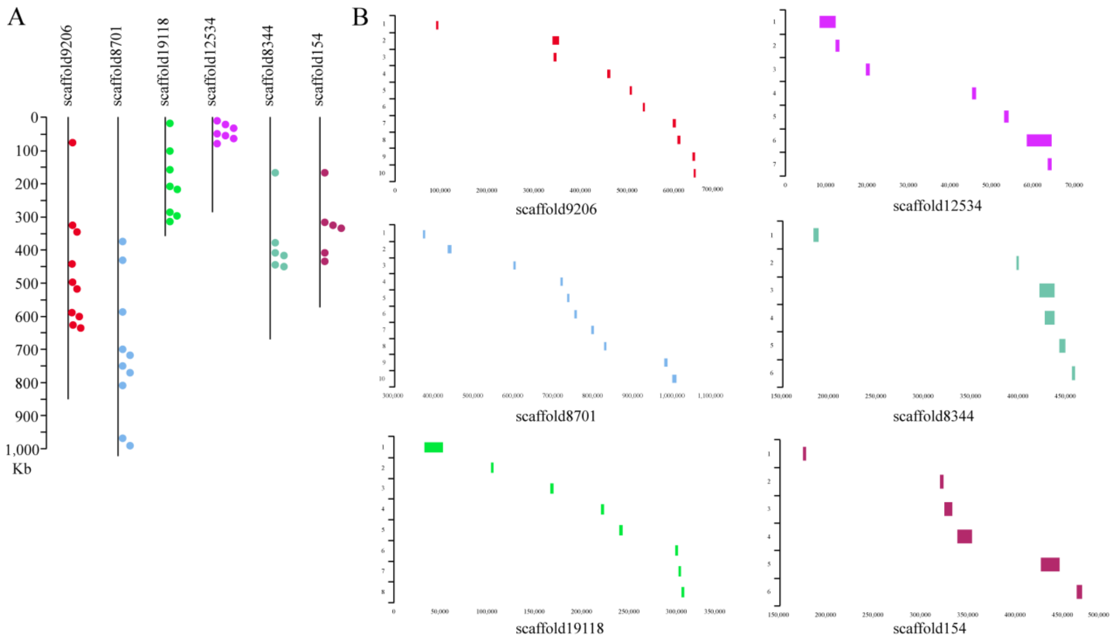

Figure S24 Distribution (A) and detail (B) of putative disease resistance gene clusters on six scaffolds in *A. ipaensis*

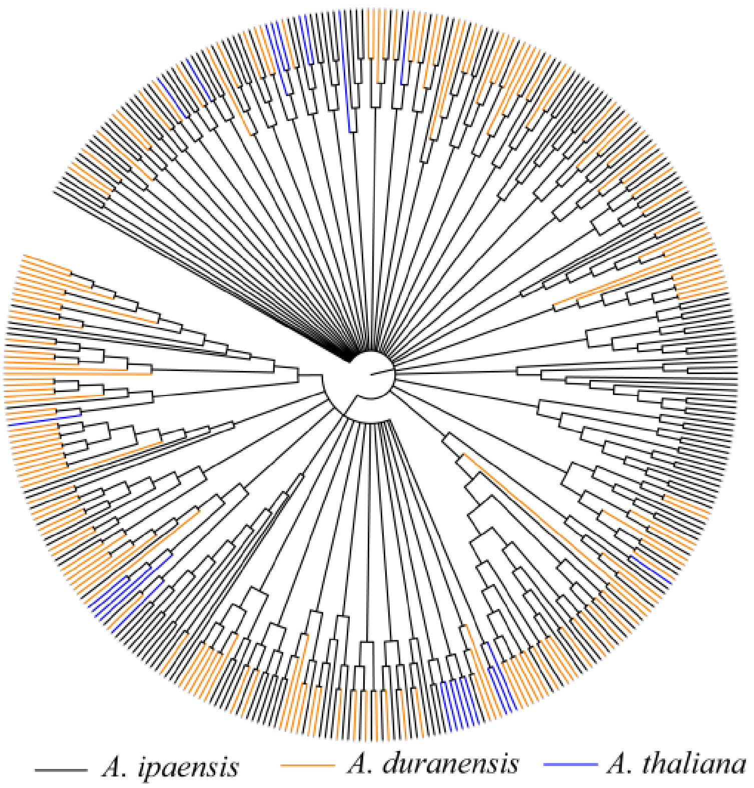

Figure S25 Phylogenetic tree of NBS-LRR genes identified in *A. ipaensis*, *A. duranensis*, and *A. thaliana*

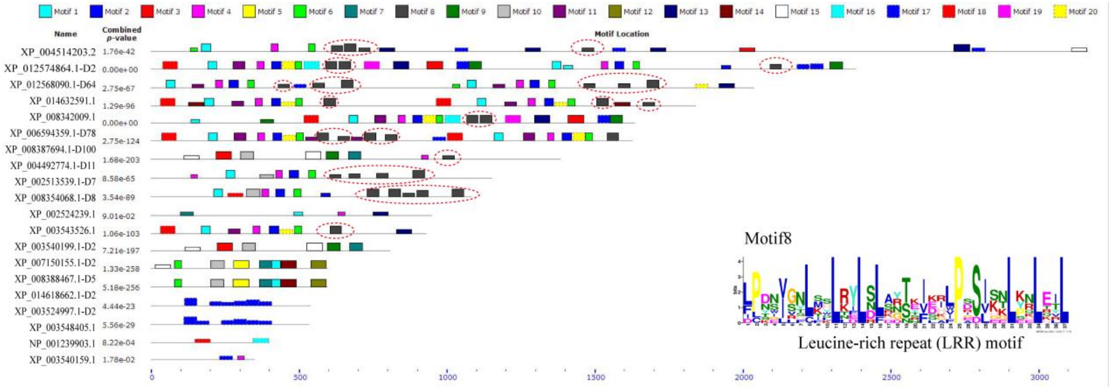

Figure S26 Distribution of conserved motifs of the top 20 homologous NBS-LRR genes in *A. ipaensis*. Red dashed circles represent leucine-rich repeat motifs (Motif 8).

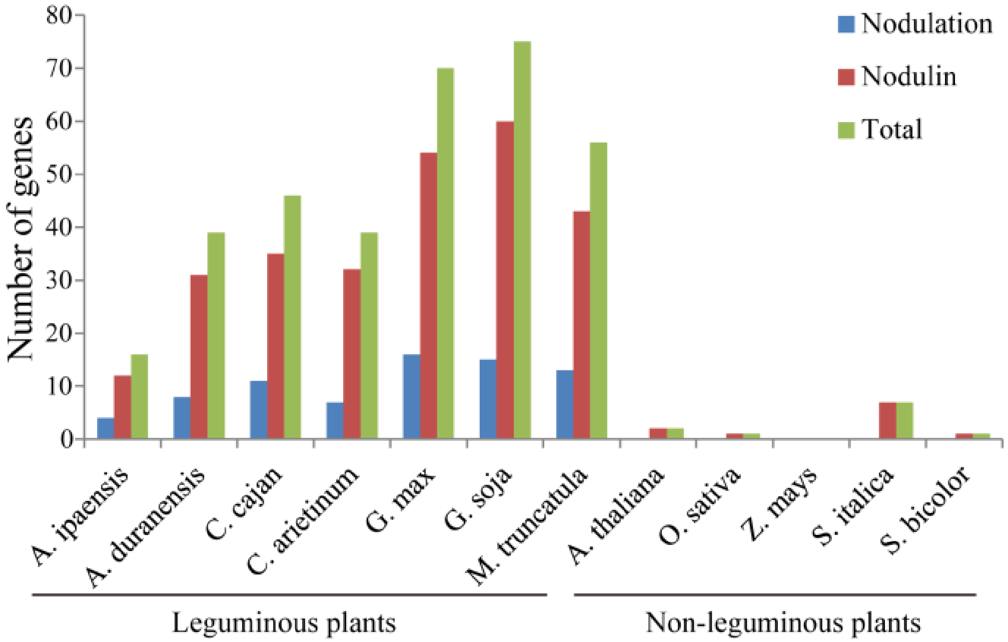

Figure S27 Number of nitrogen-fixation related genes in leguminous and non-leguminous plants

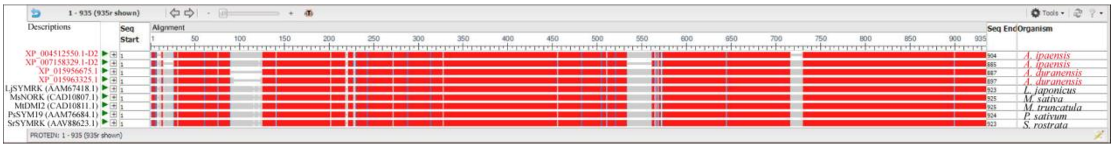

Figure S28 Amino acid alignment of the homologous genes related to nodule development in *A. ipaensis*, *A. duranensis* and other plant species. Red boxes represent completely matched protein sequences. Gray and blue boxes indicate unmatched protein sequences.

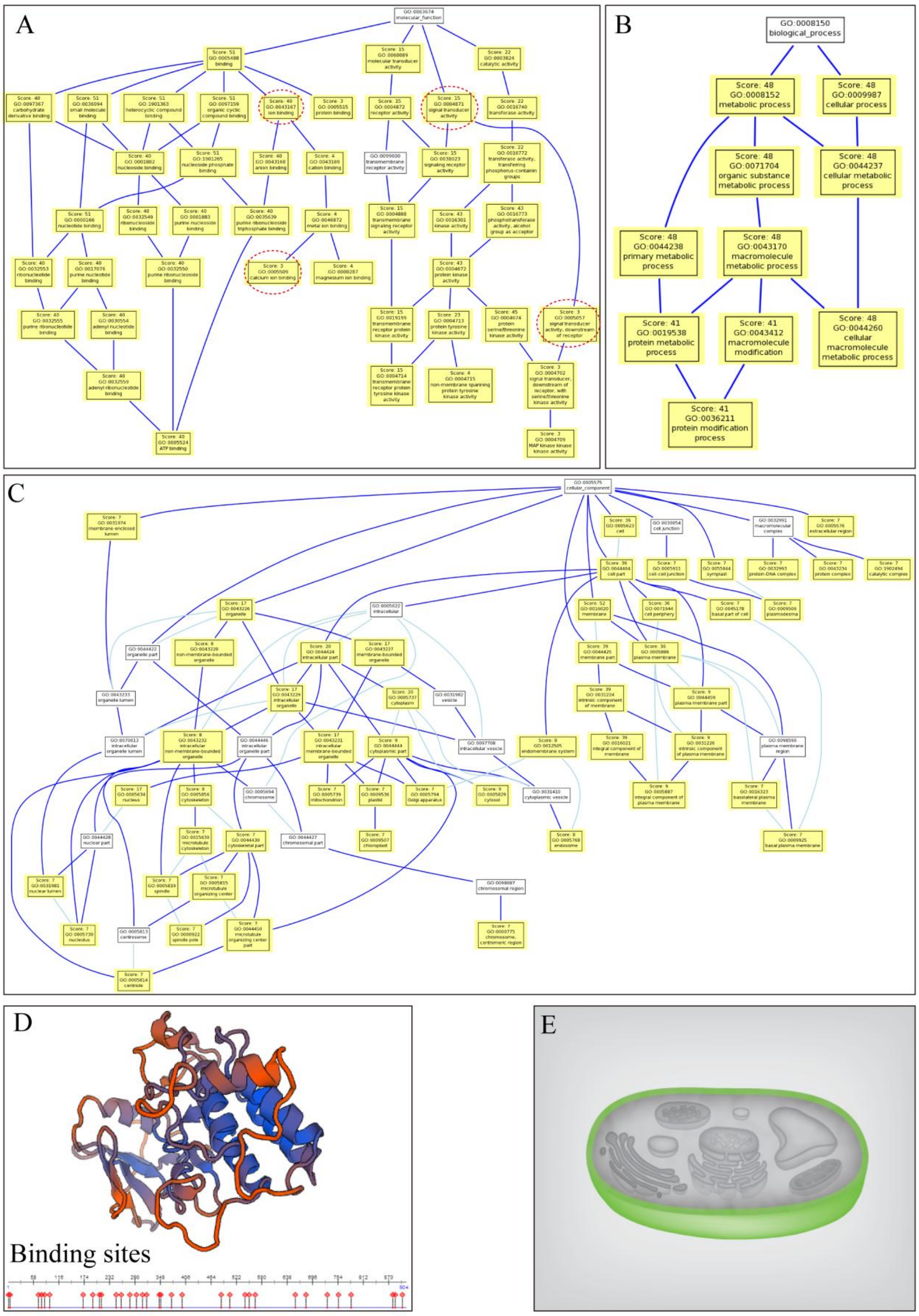

Figure S29 GO terms for nodule development homologous gene (XP_004512550.1-D2 (*A. ipaensis*)), a downstream lecine-rich repeat receptor (LRR) kinase, from *A. ipaensis* genome. A: Molecular function ontology. Red dashed circle represent the protein functions possible involving in nodule development signal reception and transmission. B: Biological process ontology. C: Cellular component ontology. D: Model of protein tertiary structure and binding sites. E: Protein subcellular location.

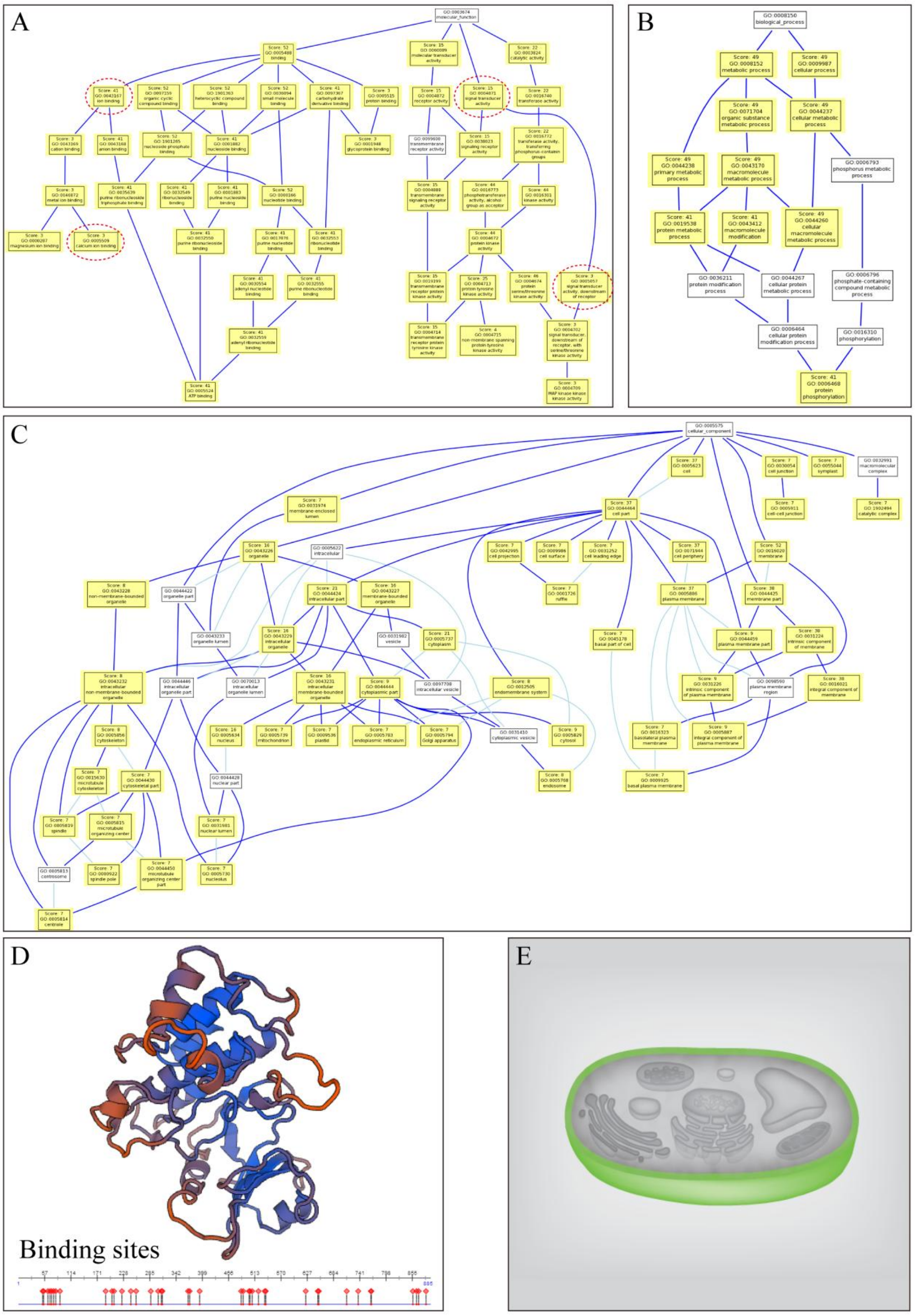

Figure S30 GO terms for nodule development homologous gene (XP_007158329.1-D2 (*A. ipaensis*)), a downstream lecine-rich repeat receptor (LRR) kinase, from *A. ipaensis* genome. A: Molecular function ontology. Red dashed circle represent the protein functions possible involving in nodule development signal reception and transmission. B: Biological process ontology. C: Cellular component ontology. D: Model of protein tertiary structure and binding sites. E: Protein subcellular location.

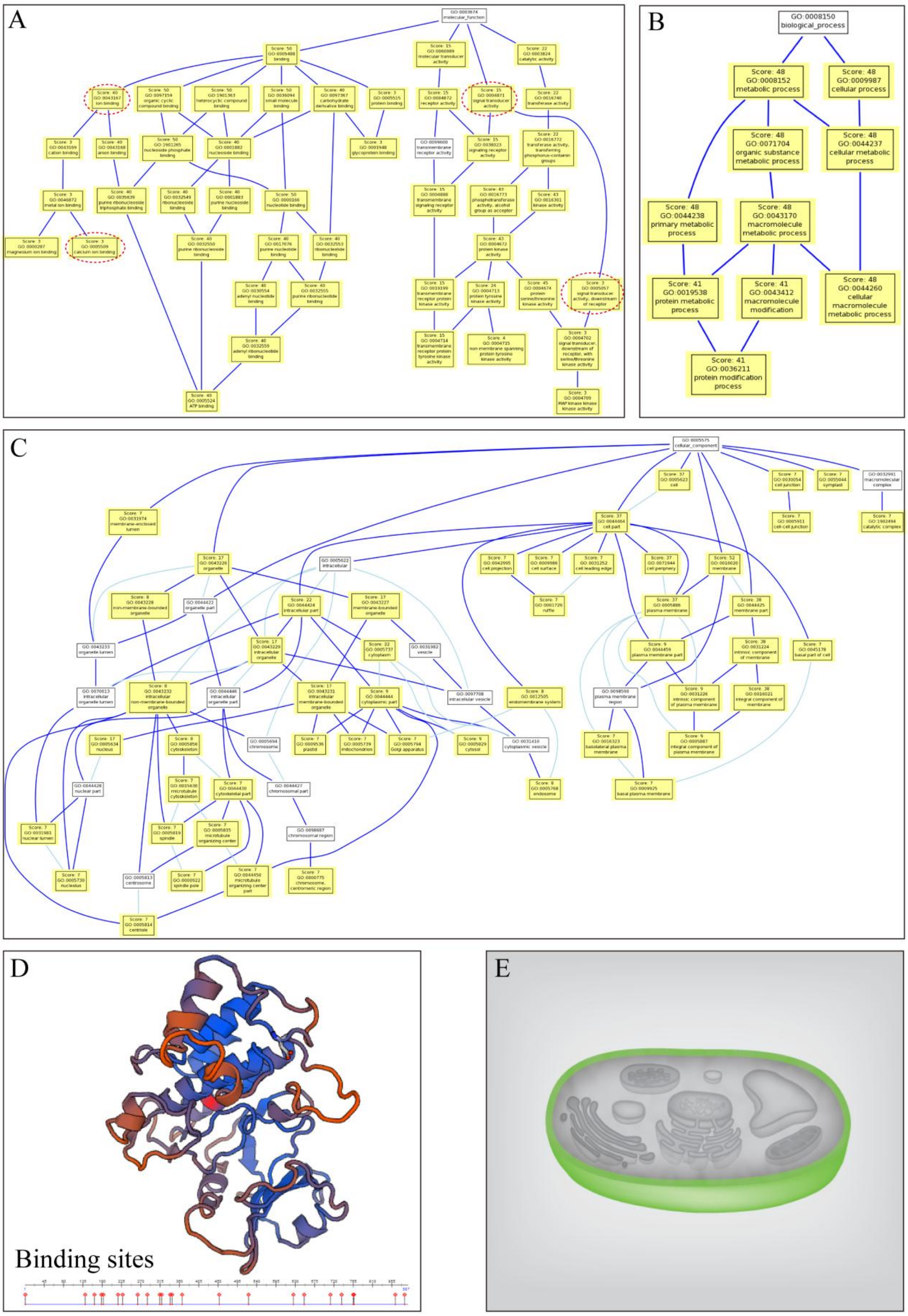

Figure S31 GO terms for nodule development homologous gene (XP_015956675.1(*A. duranensis*)), a downstream lecine-rich repeat receptor (LRR) kinase, from *A. duranensis* genome. A: Molecular function ontology. Red dashed circle represent the protein functions possible involving in nodule development signal reception and transmission. B: Biological process ontology. C: Cellular component ontology. D: Model of protein tertiary structure and binding sites. E: Protein subcellular location.

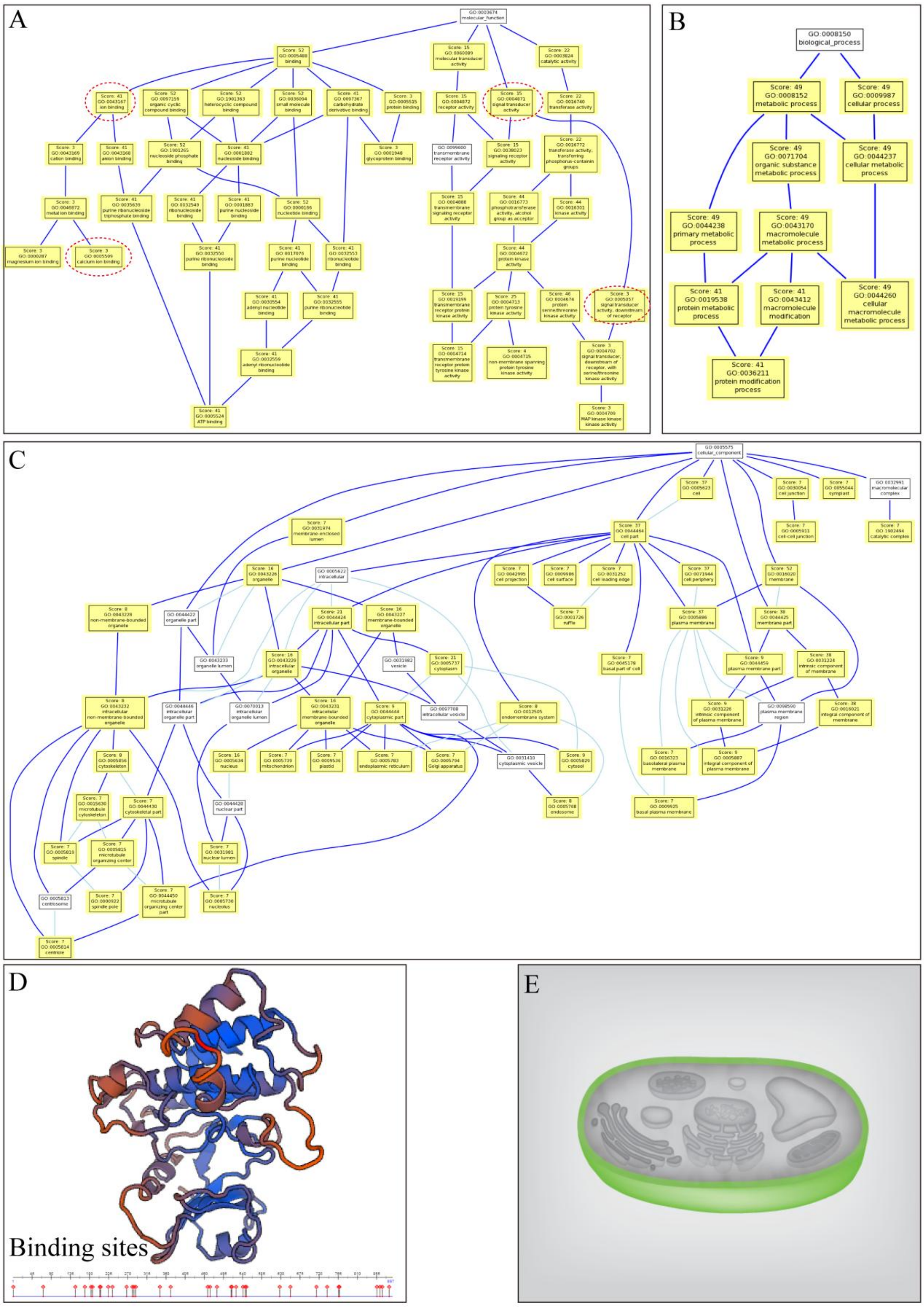

Figure S32 GO terms for nodule development homologous gene (XP_015963325.1(*A. duranensis*)), a downstream lecine-rich repeat receptor (LRR) kinase, from *A. duranensis* genome. A: Molecular function ontology. Red dashed circle represent the protein functions possible involving in nodule development signal reception and transmission. B: Biological process ontology. C: Cellular component ontology. D: Model of protein tertiary structure and binding sites. E: Protein subcellular location.

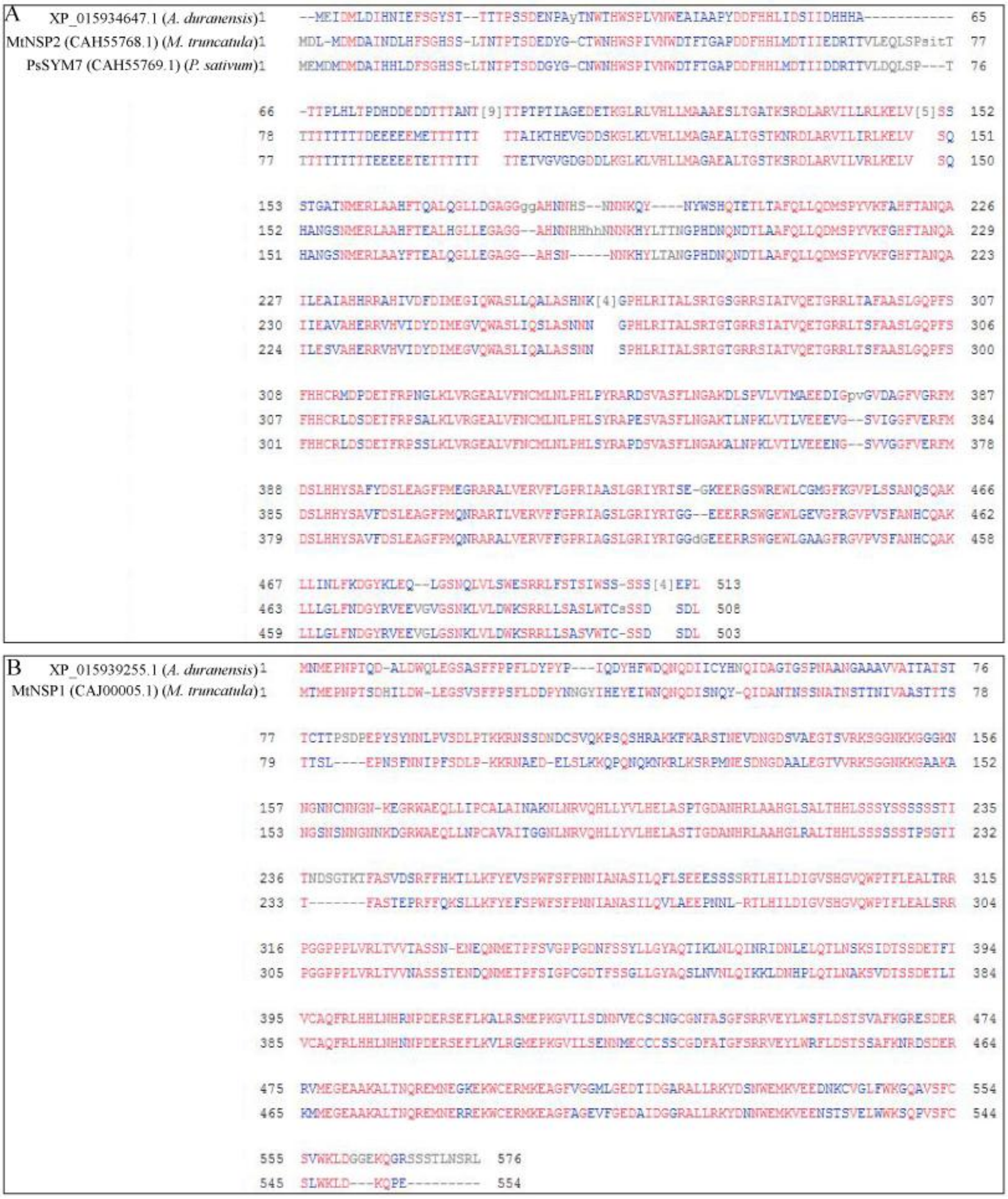

Figure S33 Amino acid alignment of transcription factor homologous genes related to nodule development in *A. duranensis* and other plant species

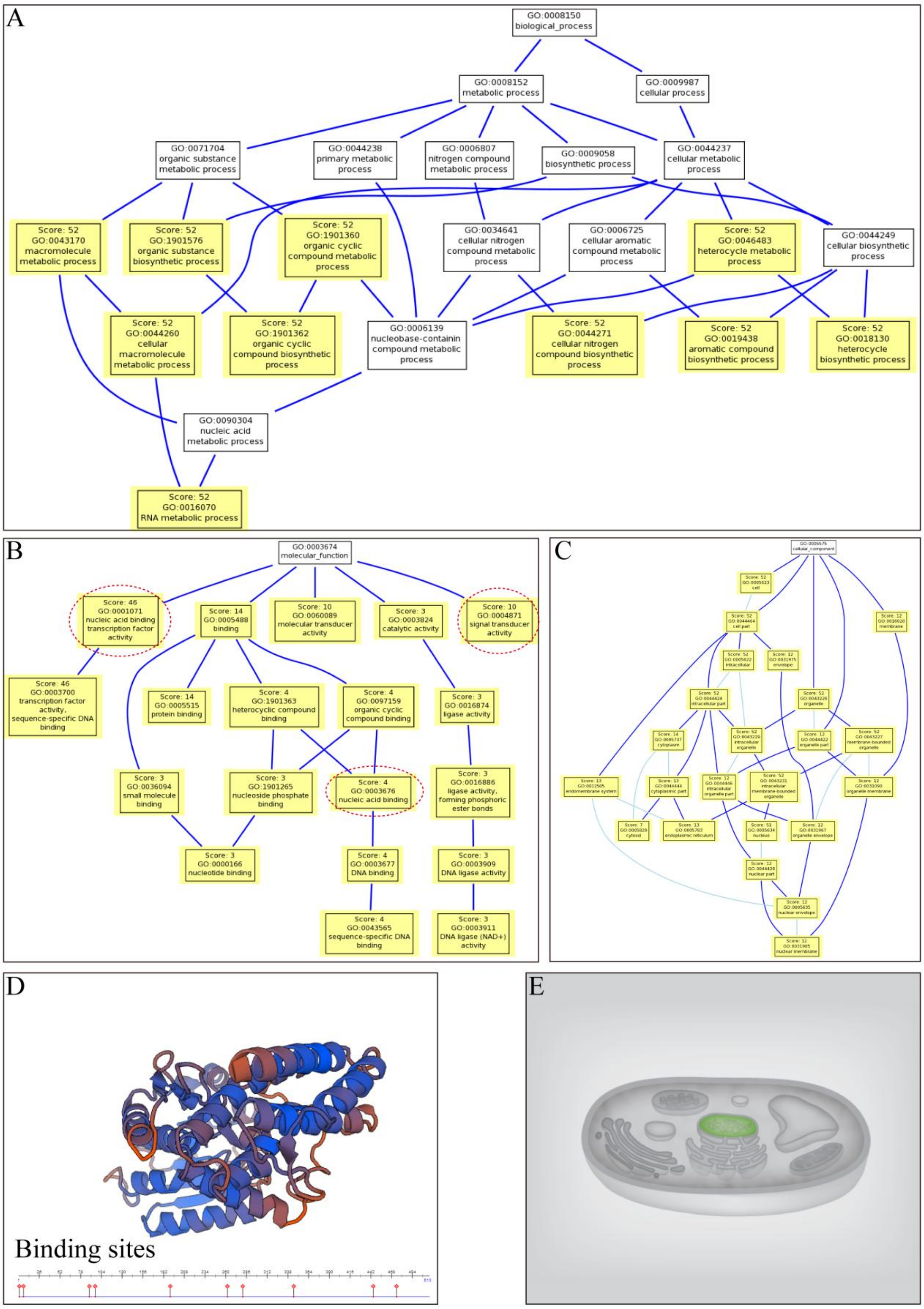

Figure S34 GO terms for nodule development homologous gene (XP_015934647.1(*A. duranensis*)), a transcription factor, from *A. duranensis* genome. A: Biological process ontology. B: Molecular function ontology. Red dashed circle represent the protein functions possible involving in nodule development signal reception and transmission. C: Cellular component ontology. D: Model of protein tertiary structure and binding sites. E: Protein subcellular location.

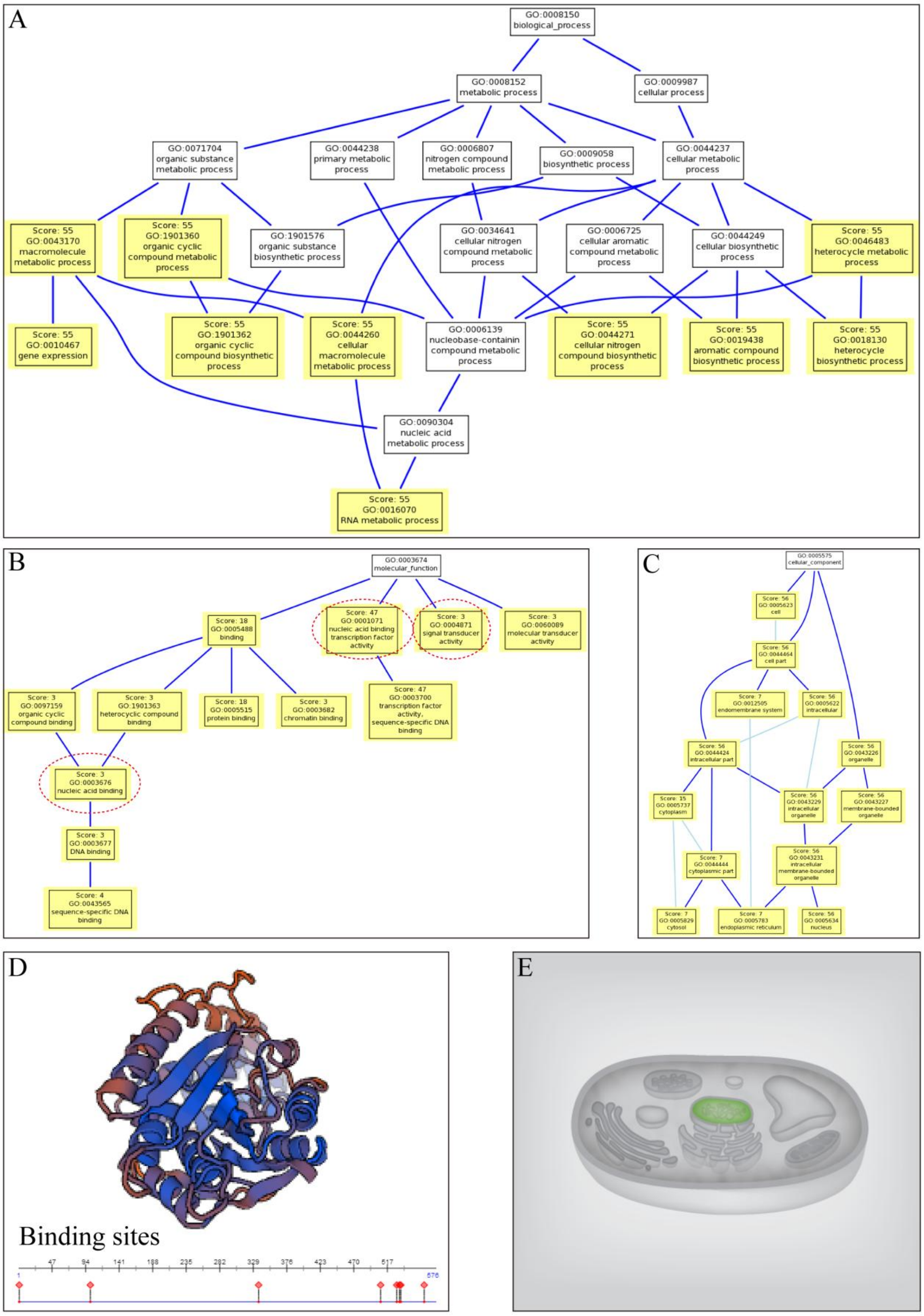

Figure S35 GO terms for nodule development homologous gene (XP_015939255.1(*A. duranensis*)), a transcription factor, from *A. duranensis* genome. A: Biological process ontology. B: Molecular function ontology. Red dashed circle represent the protein functions possible involving in nodule development signal reception and transmission. C: Cellular component ontology. D: Model of protein tertiary structure and binding sites. E: Protein subcellular location.

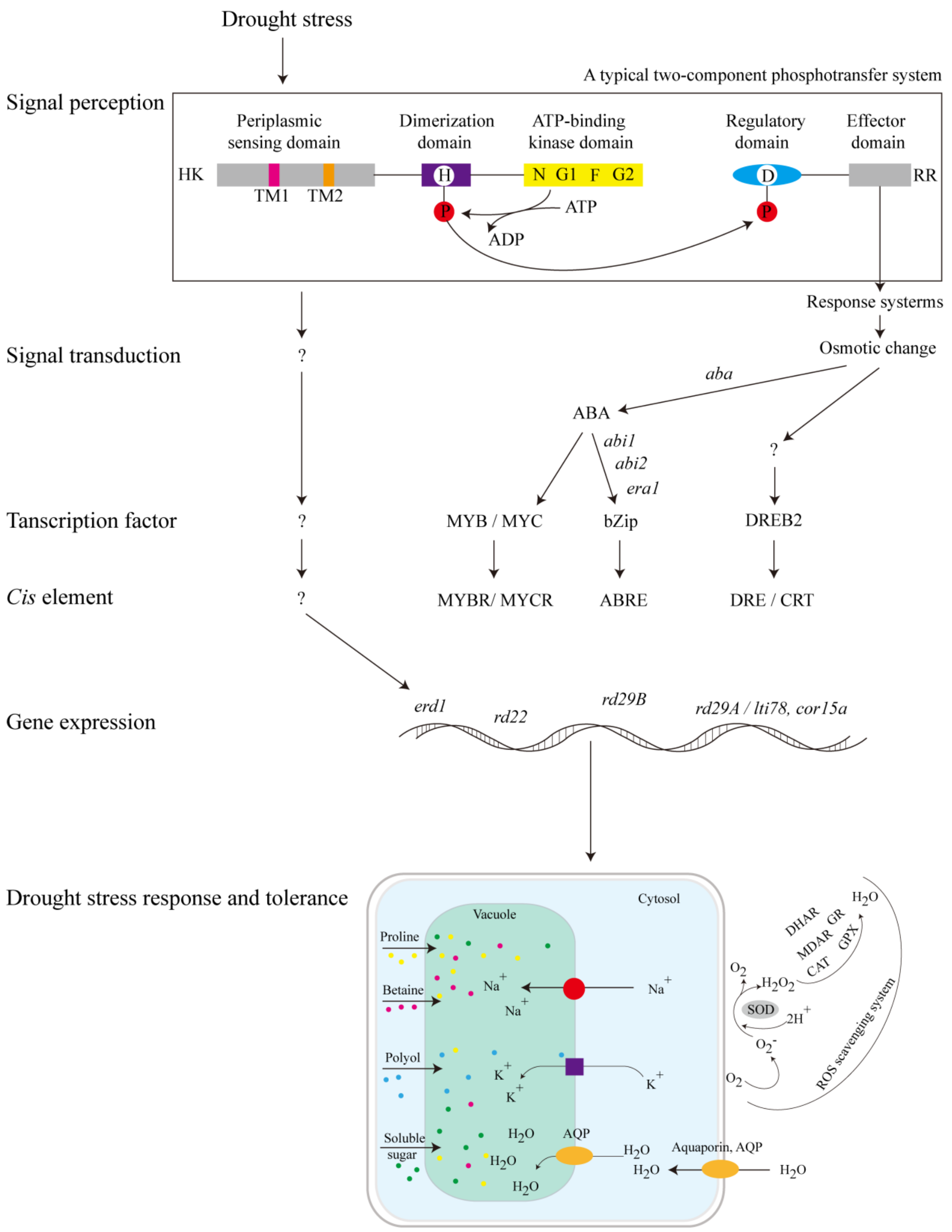

Figure S36 Transcription factors involved in drought stress regulation network in plants. A typical two-component phosphotransfer system of signal perception was adapted from West AH and Stock AM [1]. The signal pathway from transduction to gene expression was adapted from Shinozaki K, et al [2].

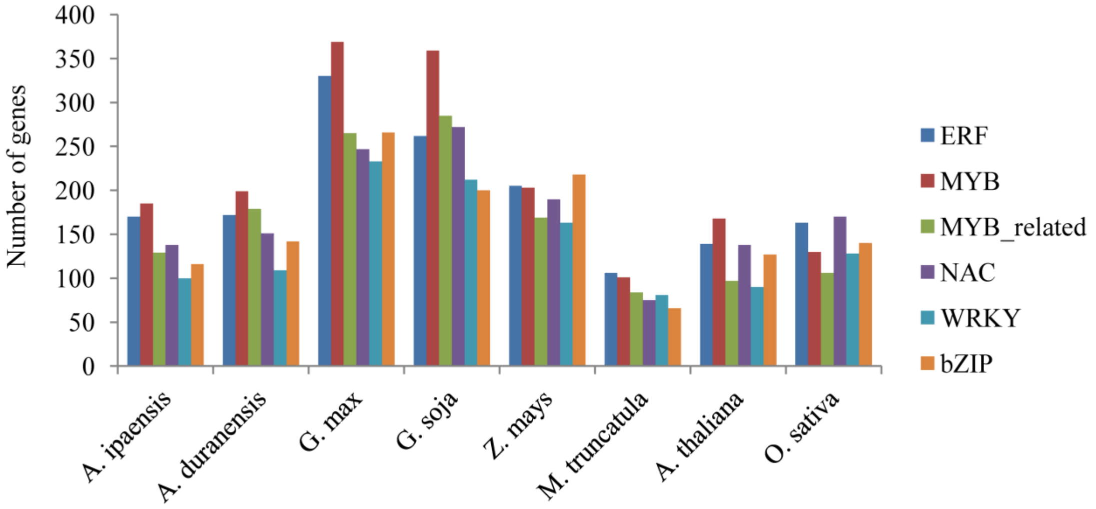

Figure S37 Summary of drought adaptation transcription factors in upland crops and hygrophilous plants

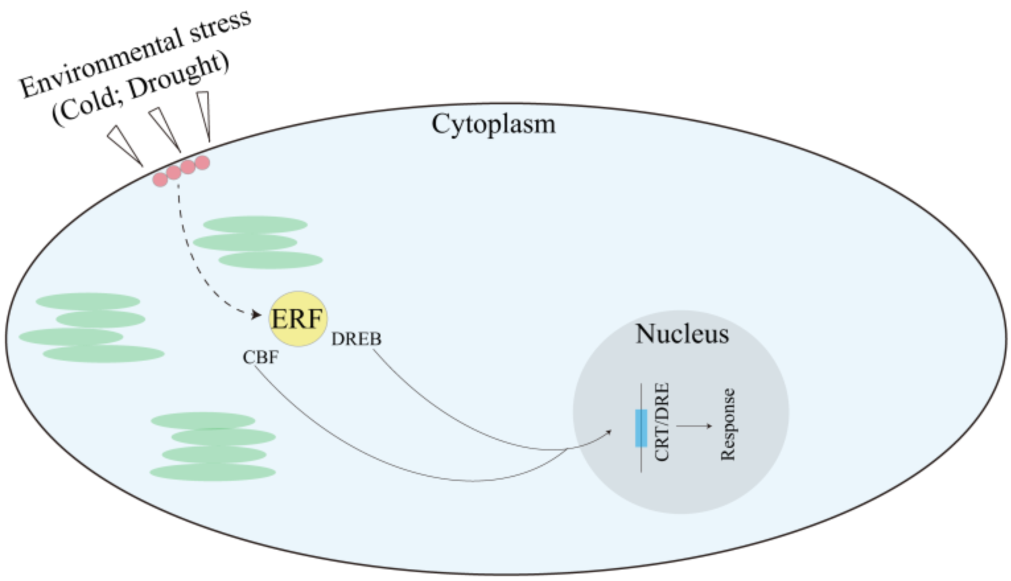

Figure S38 Mechanism of stress responses and signals linked to ERF transcription factors in plants. The outline of signals linked to ERF transcription factors was adapted from Singh KB, et al [3].

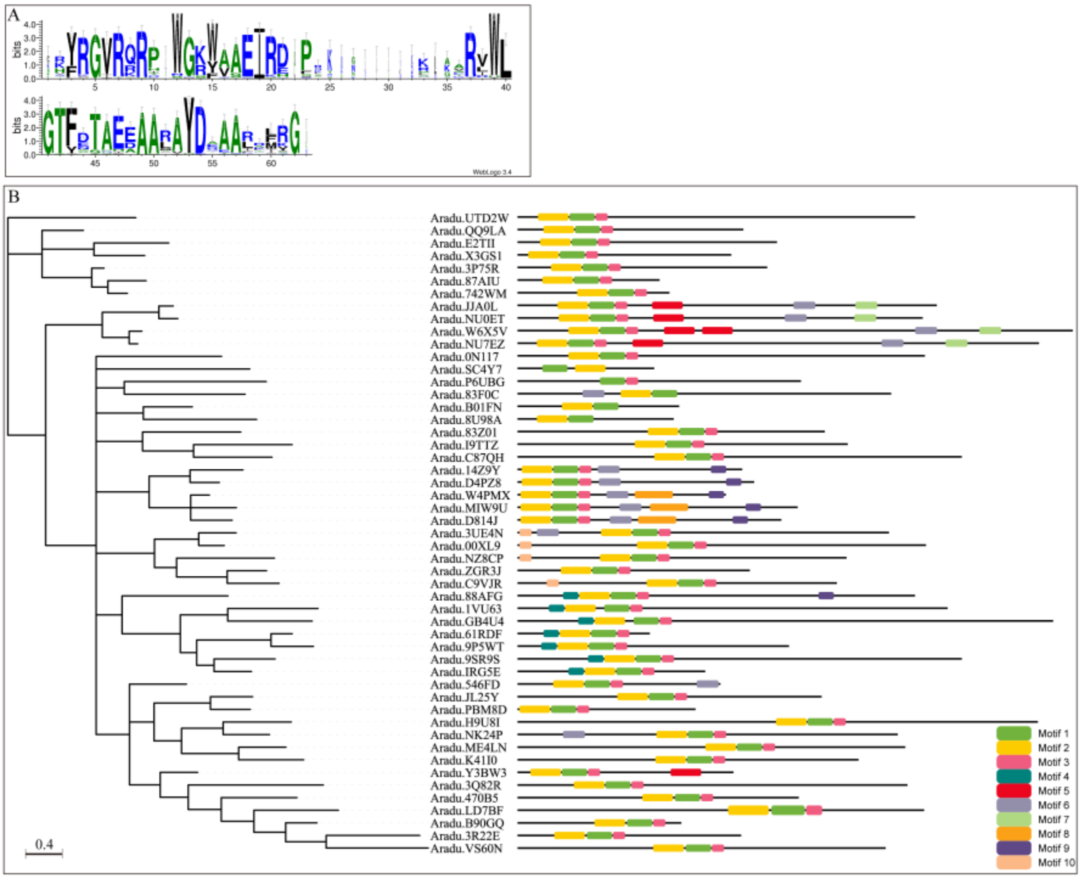

Figure S39 ERF family proteins of *A. duranensis* identified from the PlantTFDB. A, DNA binding domain; B, Phylogenetic tree and motif of ERF family proteins

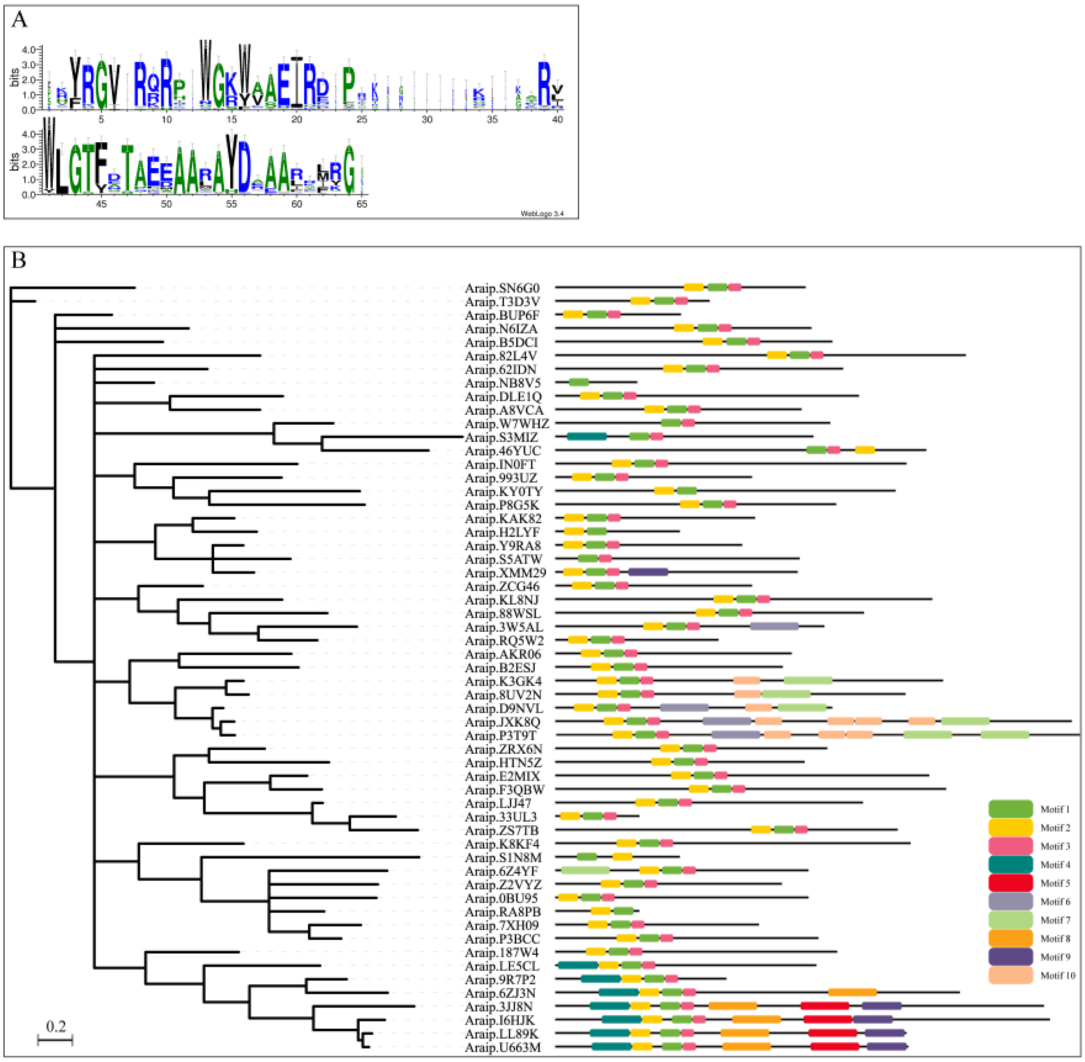

Figure S40 ERF family proteins of *A. ipaensis* identified from the PlantTFDB. A, DNA binding domain; B, Phylogenetic tree and motif of ERF family proteins

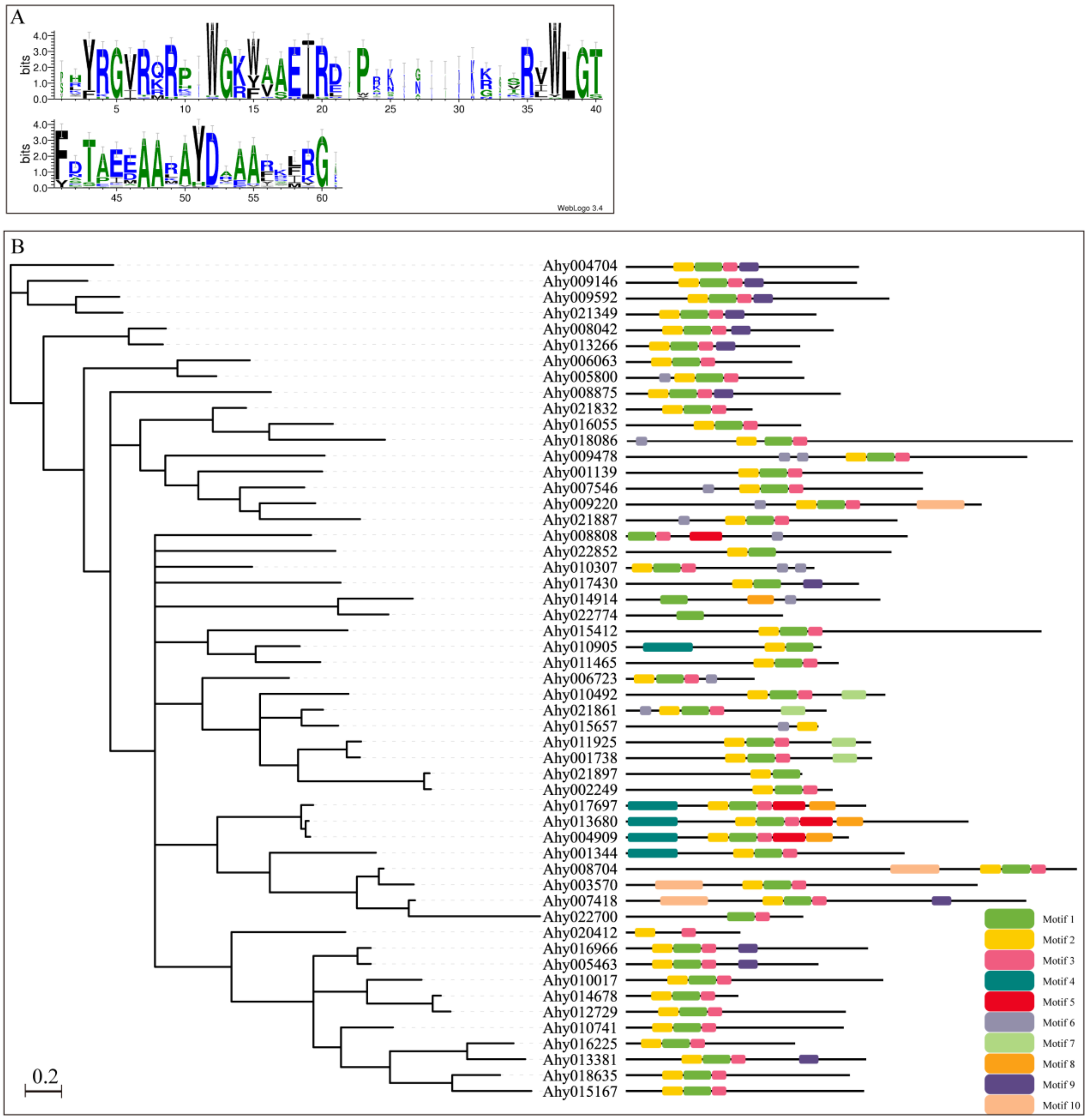

Figure S41 ERF family proteins of *A. hypogaea* identified from the PlantTFDB. A, DNA binding domain; B, Phylogenetic tree and motif of ERF family proteins

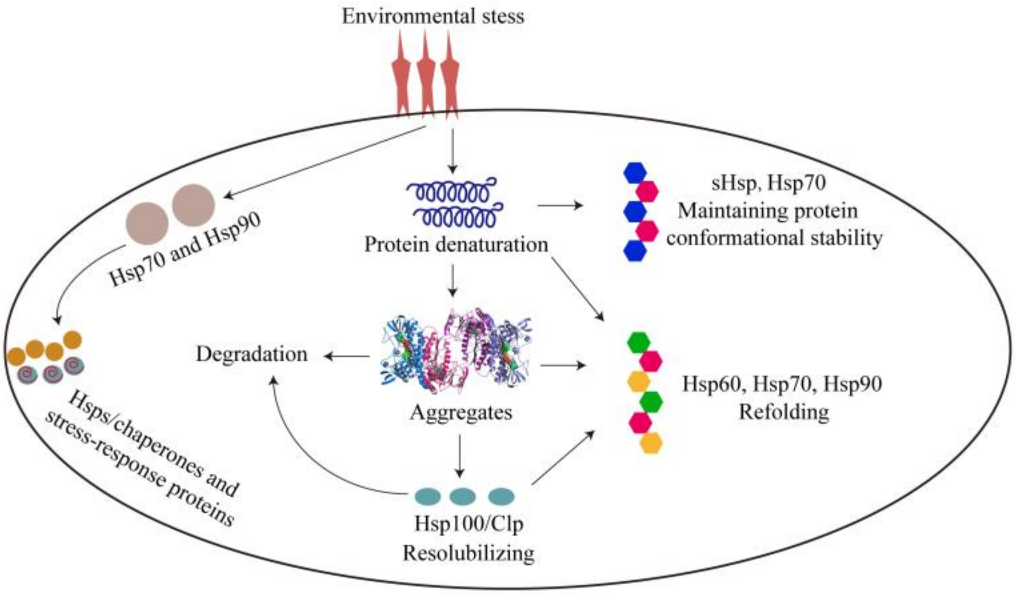

Figure S42 Network of heat-shock protein (Hsp)/chaperone in the environmental stress condition. The network was adapted from Wang W, et al [4].

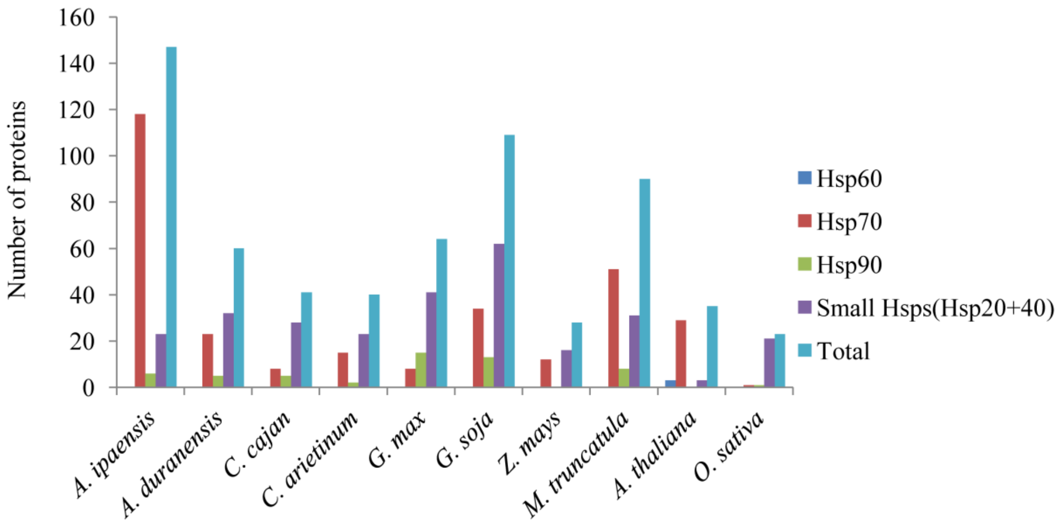

Figure S43 Summary of heat-shock proteins (Hsps)/chaperones in different upland crops and hygrophilous plants

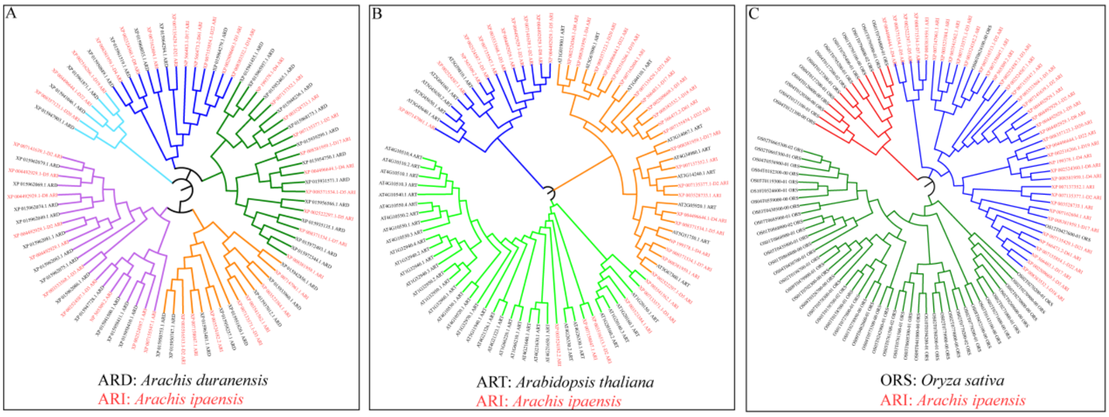

Figure S44 Phylogenetic trees of subtilisin-like protease (SDD1) genes in *A.ipaensis* and other plants

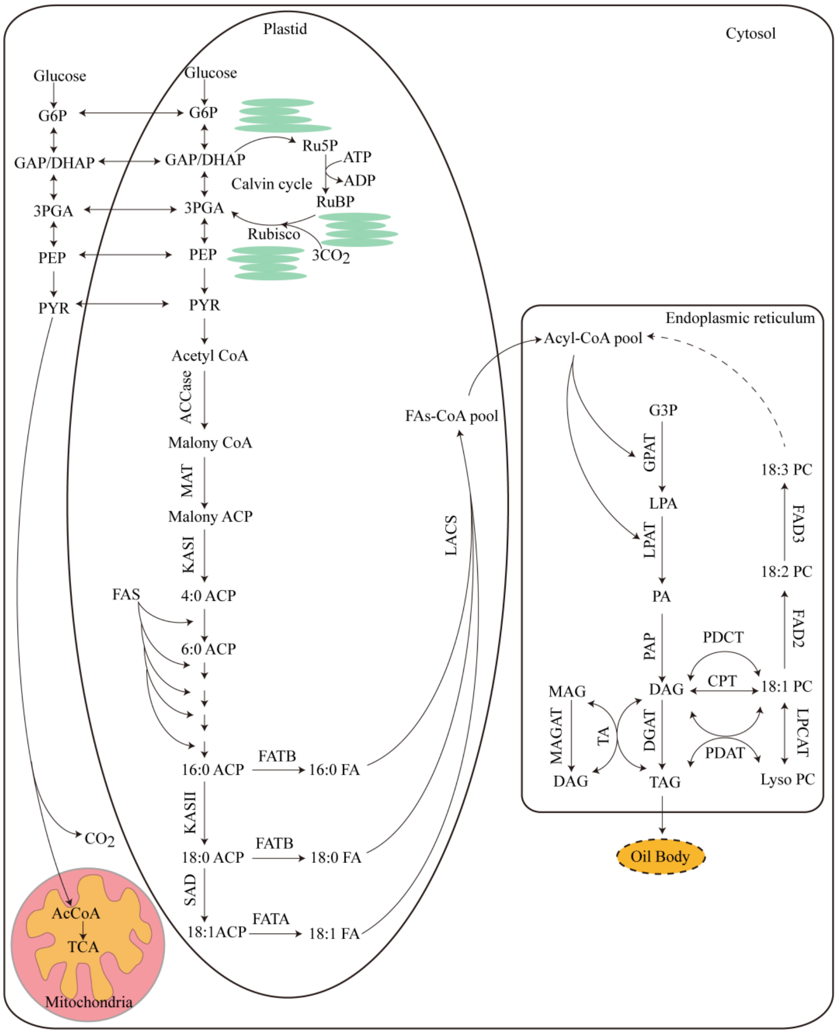

Figure S45 Metabolic pathways for triacylglycerol (TAG) biosynthesis in oil plant seeds. Main enzymes involved in the pathway were listed top to arrows in each sequential order: ACCase, Acetyl-CoA carboxylase; MAT, Plastidial malonyl-CoA; KASI, Ketoacy-ACP synthase I; FAS, Fatty acid synthase; KAS II, Ketoacy-ACP synthase II; SAD, Stearoyl-ACP desaturase; FATA, Acyl-ACP thioesterase; FATB, Acyl-ACP thioesterase; LACS, Long-chain acyl-CoA synthetase; GPAT, Glycerol-3-phosphate acyhransferase; LPAT, Lysophosphatidic acid acyhransferase; PAP, Phosphatidic acid phosphatas; DGAT, Diacylglycerol acyhransfera; CPT, Choline phosphotransferase; PDCT, Phosphatidylcholine diacylglycerol cholinephosphotransferase; PDAT, hospholipid diacylglycerol acyhransferase; LPCAT, Lysophosphatidylcholine acyltransferase; FAD2, ER oleate desaturase; FAD3, Endoplasmic Reticulum linoleate desaturase. Key metabolites involved in the pathway were listed under arrow ends: G6P, Glucose -6- phosphate; GAP, 3 phosphoric acid glycerol aldehyde; PEP, Phosphoenolpyruvate; PYR, Pyruvate; ACP, Acyl carrier protein; FA, Fatty acid; G3P, Glycerol 3 phosphate; LPA, Lysophosphatidic acid; PA, Phosphatidic acid; DAG, Diacylglycer; TAG, Triacylglycerol; PC, Phosphatidylcholine.

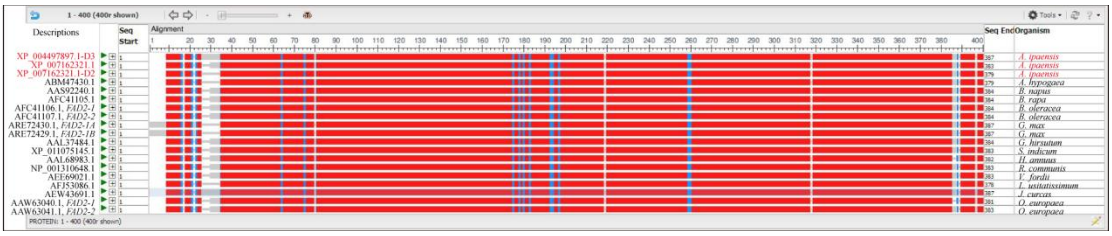

Figure S46 Multiple amino acid sequence alignment of *FAD2* homologous genes among *A. ipaensis* and oil seed plants. Red boxes represent completely matched protein sequences. Gray and blue boxes indicate unmatched protein sequences.

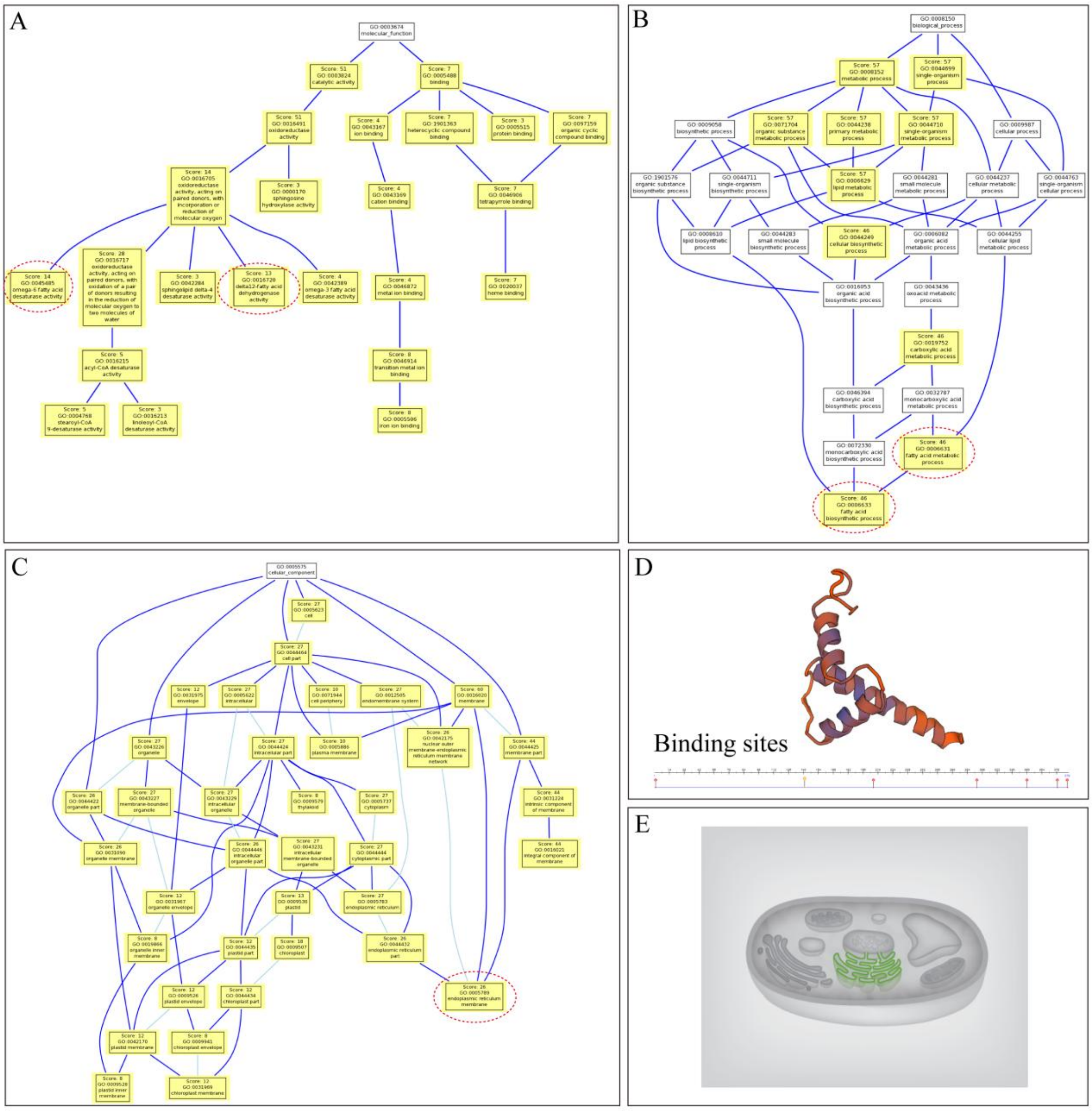

Figure S47 GO terms for *FAD2* homologous gene (XP_007162321.1-D2) in *A. ipaensis*. A: Molecular function ontology. Red dashed circle represent the protein functions possible involves in fatty acid desaturase activity. B: Biological process ontology. Red dashed circle represent the protein involves in fatty acid biosynthetic process. C: Cellular component ontology. Red dashed circle represent the protein is a component of the endoplasmic reticulum membrane D: Model of protein tertiary structure and binding sites. E: Protein subcellular location.

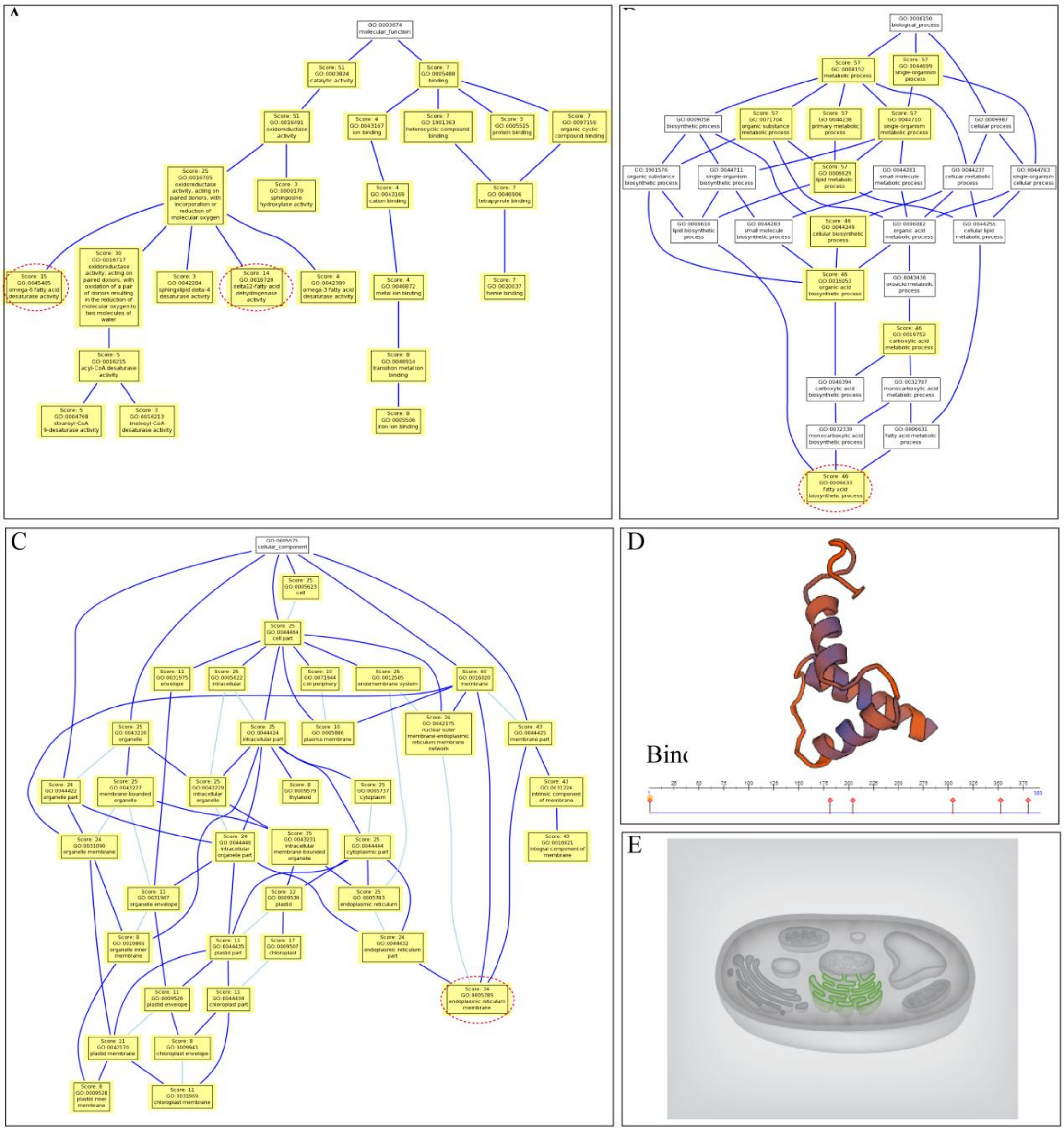

Figure S48 GO terms for *FAD2* homologous gene (XP_007162321.1) in *A. ipaensis*. A: Molecular function ontology. Red dashed circle represent the protein functions possible involves in fatty acid desaturase activity. B: Biological process ontology. Red dashed circle represent the protein involves in fatty acid biosynthetic process. C: Cellular component ontology. Red dashed circle represent the protein is a component of the endoplasmic reticulum membrane D: Model of protein tertiary structure and binding sites. E: Protein subcellular location.

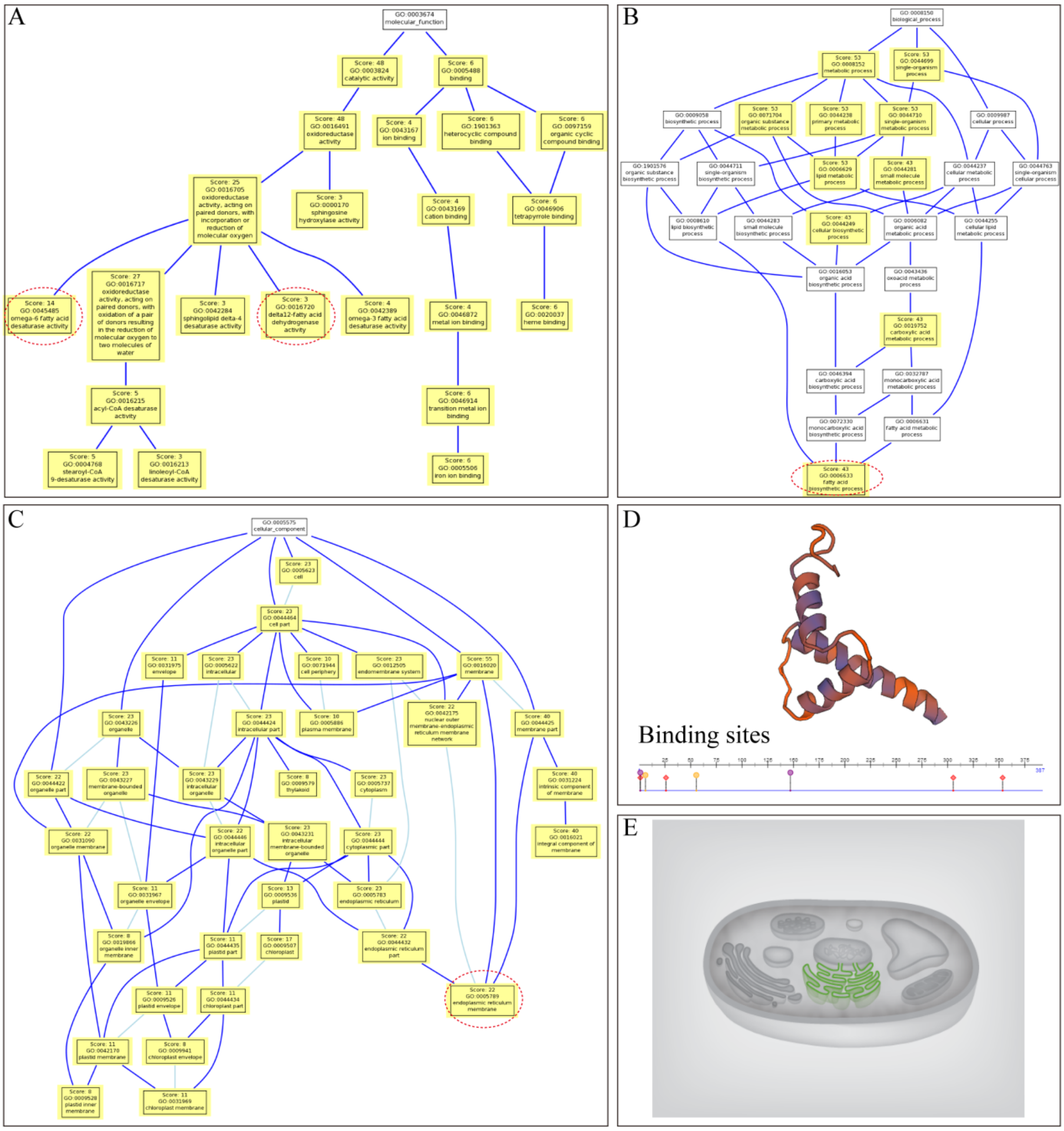

Figure S49 GO terms for *FAD2* homologous gene (XP_004497897.1-D3) in *A. ipaensis*. A: Molecular function ontology. Red dashed circle represent the protein functions possible involves in fatty acid desaturase activity. B: Biological process ontology. Red dashed circle represent the protein involves in fatty acid biosynthetic process. C: Cellular component ontology. Red dashed circle represent the protein is a component of the endoplasmic reticulum membrane D: Model of protein tertiary structure and binding sites. E: Protein subcellular location.

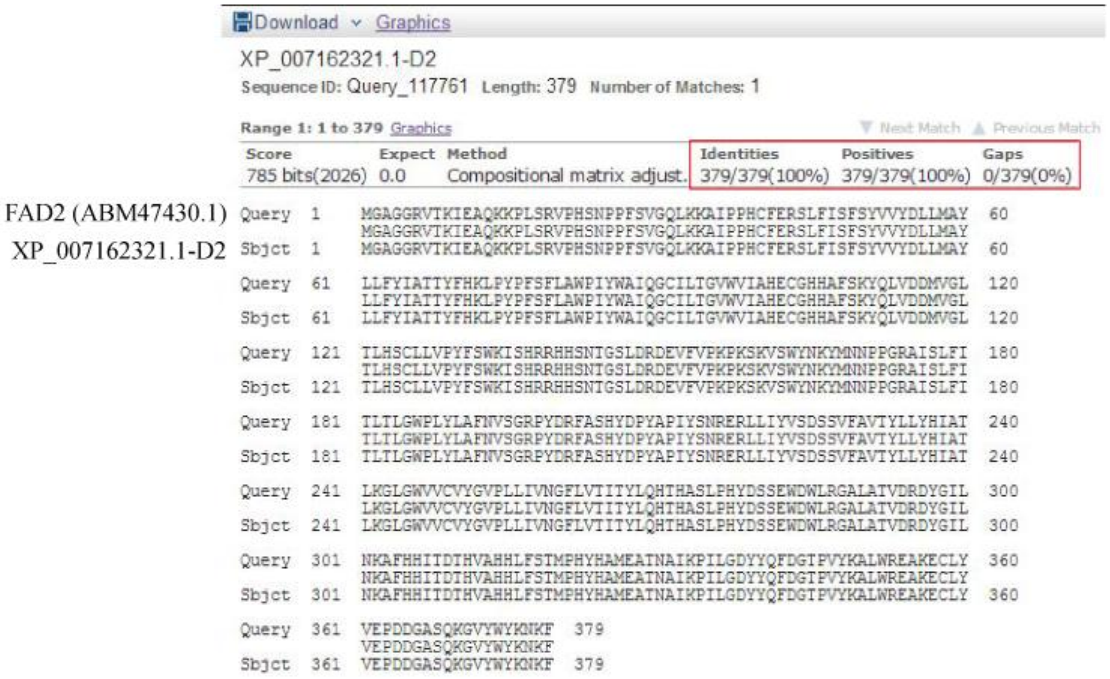

Figure S50 Protein sequence alignment between FAD2 in *A. hypogaea* and its homologous gene in *A. ipaensis*

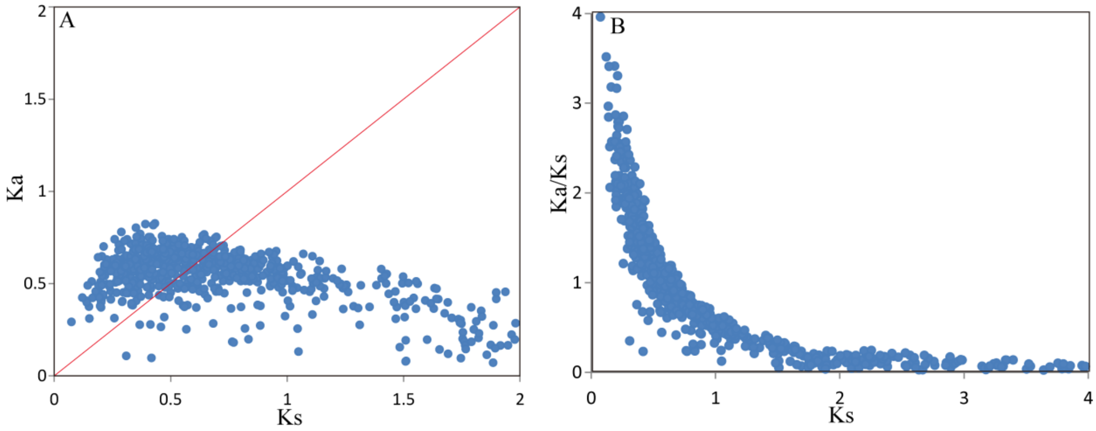

Figure S51 Scatterplot of *Ks* vs. *Ka* (A) and *Ks* vs. *Ka*/*Ks* (B) of oil synthesis homologous genes between *A. ipaensis* and *A. thaliana*

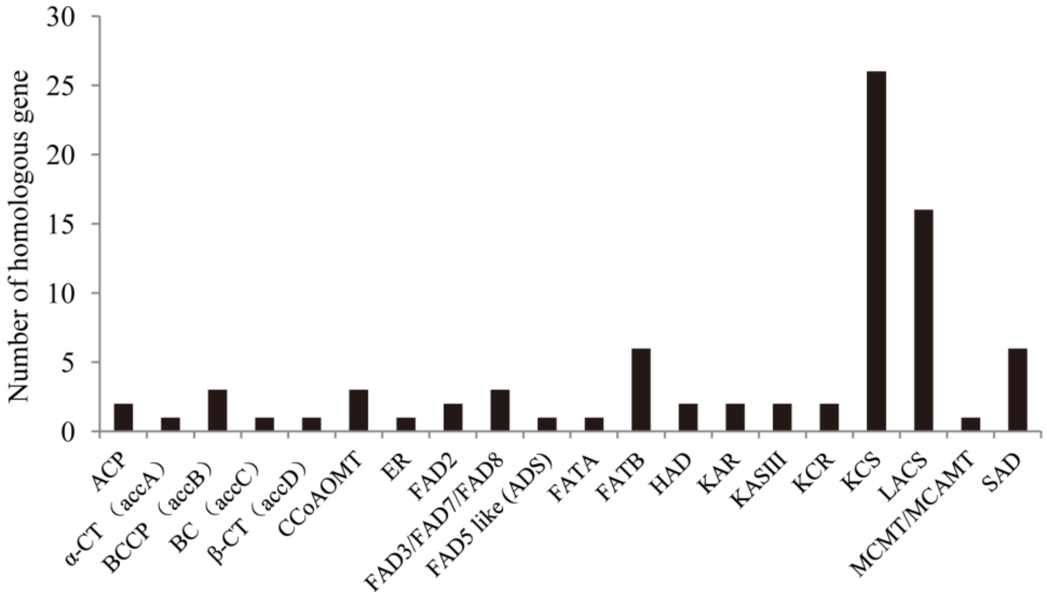

Figure S52 Number of oil synthesis homologous genes with high *Ka*/*Ks* values in *A. ipaensis*

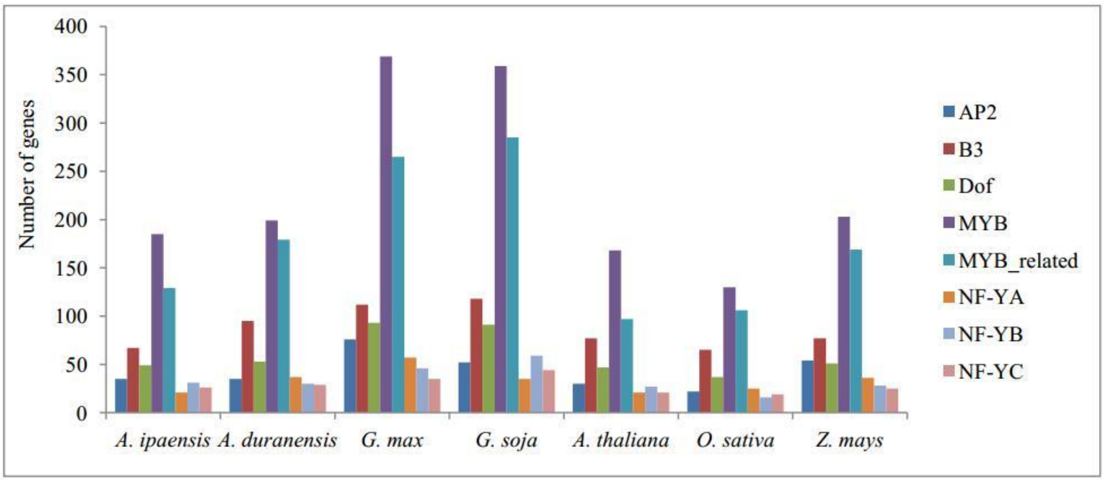

Figure S53 Summary of oil synthesis transcription factors in *A. ipaensis* and other plants

Supplementary references:

1. West, A. H., and Stock, A. M. (2001). Histidine kinases and response regulator proteins in two-component signaling systems. Trends Biochem Sci. 26:369-376.

2. Shinozaki, K., and Yamaguchi-Shinozaki, K. (2000). Molecular responses to dehydration and low temperature: differences and cross-talk between two stress signaling pathways. Curr Opin Plant Biol. 3:217-223.

3. Singh, K. B., Foley, R. C., Oñate-Sánchez, L. (2002). Transcription factors in plant defense and stress responses. Curr Opin Plant Biol. 5:430-436.

4. Wang, W., Vinocur, B., Shoseyov, O., Altman, A. (2004). Role of plant heat-shock proteins and molecular chaperones in the abiotic stress response. Trends Plant Sci. 9:244-252.
